# Photobattery Architectures to Suppress Charge Recombination

**DOI:** 10.1021/acs.nanolett.6c02373

**Published:** 2026-10-13

**Authors:** Kohei Shimokawa, Byung-Man Kim, Arvind Pujari, Hooman Abbasi, Tetsu Ichitsubo, Michael De Volder

**Affiliations:** † Institute for Manufacturing, Department of Engineering, 2152 University of Cambridge, Cambridge CB3 0FE, U.K.; ‡ Frontier Research Institute for Interdisciplinary Sciences, Tohoku University, Sendai 980-8578, Japan; § Institute for Materials Research, Tohoku University, Sendai 980-8577, Japan; ∥ Cavendish Laboratory, Department of Physics, 2152 University of Cambridge, Cambridge CB3 0HE, U.K.

**Keywords:** Photobatteries, Energy harvesting and storage, Photoelectrochemistry, Cathode materials

## Abstract

Integrating solar energy conversion and storage into single-unit photobatteries offers compact energy supplies. However, achieving sufficient photovoltage to charge batteries without external bias remains a critical bottleneck for these devices. Here, we demonstrate that the photovoltage in Zn-ion photobatteries can be modulated and enhanced by selecting and structuring the photoactive materials strategically. Randomly dispersed electrode configurations are easy to manufacture but they can suffer from charge recombination. In our experiments, these yielded a negligible photovoltage of ∼0.01 V. In comparison, our proposed layer-by-layer design, integrating a solid-state dye-sensitized solar cell (DSSC) with an optimized spinel Zn–Mn oxide cathode, increased the photovoltage output to ∼1 V under 1-sun illumination. This dramatic performance enhancement is achieved by better control over interfaces and charge transport and establishes a strategy for improving the architecture of future photobatteries.

## Graphical abstract

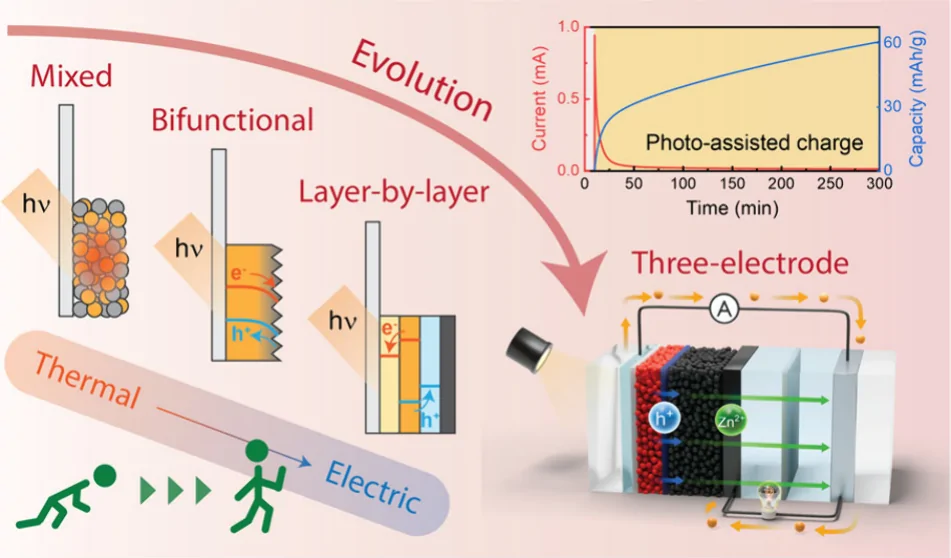

Photobatteries, which combine solar energy conversion and electrochemical storage within a single unit, represent an integrated approach toward autonomous and compact energy harvesting.^1–3^ By simplifying the external wiring and eliminating intermediary components such as power converters often employed in conventional photovoltaic–battery coupled systems, this integrated design offers potential advantages in terms of a minimized device footprint, reduced overall cost, and lower system complexity. Following the pioneering demonstrations of the photobattery concept,^4,5^ research in this area remained relatively dormant for several decades. However, a series of seminal reports^6–8^ has since reignited interest in this field, driven by the growing demand for standalone Internet of Things (IoT) devices and self-powered energy storage systems.^9^ Despite this progress, significant fundamental challenges remain, most notably the substantial photovoltage deficit and associated interfacial losses relative to the theoretical limit defined by the photoabsorber’s energy gap (typically ∼2 eV). As a result, in many devices, it is unclear if photothermal effects or true photocharging is taking place.^10^

As reported recently,^11^ numerous systems rely on randomly dispersed architectures, where bifunctional or separate pairs of photoactive and storage materials are physically blended with carbon to form a composite electrode. While such randomly mixed photoelectrodes can provide photoenhanced capacity or a gradual increase in open-circuit voltage (OCV),^12–14^ they generally suffer from excessive photovoltage losses due to nondirectional charge transport and intensive interfacial recombination across poorly defined boundaries. Although several layered designs have been demonstrated to spatially separate these functionalities,^15–20^ achieving the targeted properties of the integrated photobattery system remains elusive. A systematic design principle that bridges the gap between theoretical prospects and experimental implementation is urgently required.

To correlate the geometric structural order of photoelectrode components with photoelectrochemical output, we compared three distinct photobattery architectures (Figure 1a). An aqueous Zn-ion battery system was chosen as a model system for its inherent stability and safety.^21^ The conventional mixed photocathodes, comprising a random blend of dye-sensitized TiO_2_, spinel-type Zn–Mn oxide (ZMO), and carbon black, exhibit a negligible photovoltage (∼0.01 V), which might stem primarily from a photothermal response.^22^ The lack of a defined potential gradient in the mixture results in random-walk diffusion of charge carriers and rapid nonradiative recombination, dissipating input energy as heat.^10^ In contrast, the bifunctional film architecture shown in Figure 1a utilizes p-type Cu_2_O that introduces band bending and guides charge carriers, increasing the photovoltage to the order of 0.1 V. Another order of magnitude increase in voltage output is observed in layer-by-layer architectures based on solid-state dye-sensitized solar cell (DSSC) designs. By integrating discrete electron- and hole-transport layers (ETL/HTL) with well-defined interfaces, these architectures minimize carrier entropy and photovoltaic losses, yielding a photovoltage of approximately 1 V under 1-sun illumination. The effect of this geometric arrangement on charge-transfer is insufficiently taken into account in photobatteries, and in this paper, we will denote these three architectures as 0D-random mixed, 1D-gradient bifunctional, and 2D-interfacial layer-by-layer systems (Figure 1a).

**Figure 1:**
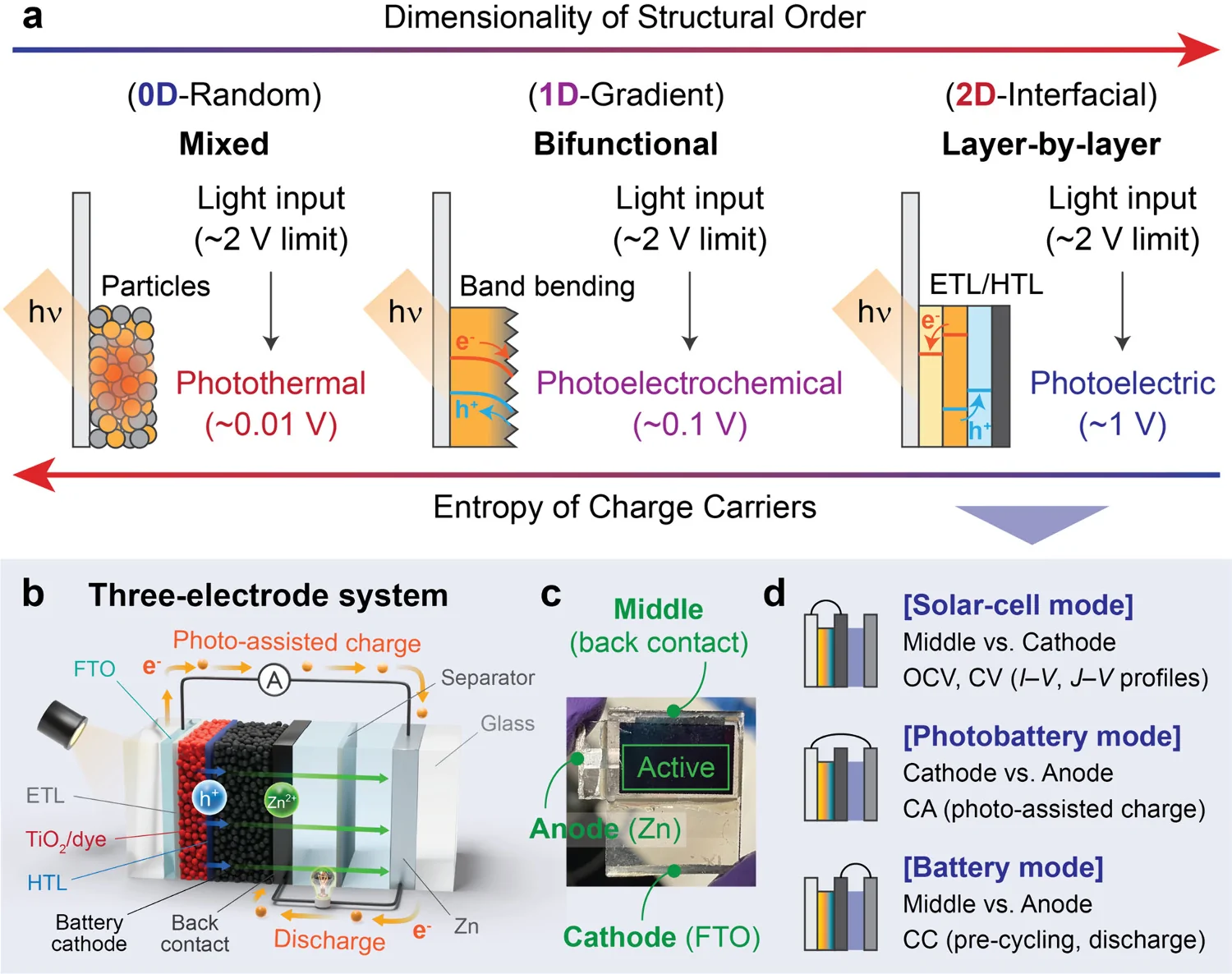
| Conceptual framework of structural evolution in photoelectrodes. (a) Structural evolution from 0D-random (mixed particles) through 1D-gradient (bifunctional film) to 2D-interfacial (layer-by-layer) architectures results in an order-of-magnitude stepwise increase in photovoltage (∼0.01, ∼0.1, and ∼1 V, respectively). This enhancement is associated with the systematic reduction in the entropy of charge carriers. While 0D-random particle contacts suffer from intensive recombination (represented as photothermal dissipation), the 2D-interfacial order with electron and hole transport layers (ETL/HTL) facilitates effective charge separation and potential confinement, approaching the energetic limit defined by the photoabsorber’s energy gap (∼2 eV). (b) Schematic of the three-electrode layer-by-layer structure (see Methods for details) and charge-transfer mechanism. Photoexcited electrons are collected via the TiO_2_ ETL (represented in white), while holes migrate through the PEDOT HTL (blue) to oxidize the ZMO cathode (black) during photoassisted charging. (c) Digital photograph of the photobattery device featuring three terminals: (photo)­cathode (FTO), anode (Zn), and middle (back contact). The total active area (green rectangle) is approximately 1.0 cm^2^. (d) Operational modes of the three-electrode configuration: solar-cell mode (middle vs cathode) for photovoltaic characterization; photobattery mode (cathode vs anode) for photoassisted charge; battery mode (middle vs anode) for dark battery precycling and discharge.

For measurements, the layer-by-layer photoelectrodes were integrated into a three-electrode system^23,24^ (Figure 1b). In this configuration, the photoactive interface and the electrochemical storage component are spatially separated yet electrically coupled, allowing for chronoamperometric photoassisted charge and subsequent galvanostatic discharge in the dark. In this study, “photoassisted” specifically denotes the utilization of photovoltage to supplement the electrochemical potential under an applied bias, consistent with the previously defined terminology.^11^ As illustrated in the device schematic and photograph (Figure 1c), three terminals comprising a (photo)­cathode (FTO), an anode (Zn), and a middle back-contact were established to regulate the (photo)­electrochemical dynamics across each functional layer. This platform enables the device to function in three distinct operational modes (Figure 1d): (i) solar-cell mode, which characterizes the photovoltaic output between the middle back-contact and the cathode; (ii) photobattery mode, governing photoassisted charge between the cathode and the Zn anode; (iii) battery mode, which manages dark discharge and electrochemical precycling between the middle back-contact and the anode. Crucially, this three-electrode configuration establishes a rigorous framework to independently evaluate and standardize the performance metrics of integrated photobatteries. While such a complex three-electrode system is required for the layer-by-layer photoelectrode due to the insulating nature of the ETL in the dark, a conventional two-electrode system is sufficient for the 0D and 1D configurations shown in Figure 1a. To ensure a consistent comparison, all configurations were evaluated using an identical 3 M zinc trifluoromethanesulfonate [Zn­(OTf)_2_] aqueous electrolyte and solar simulator (Figure S1). Importantly, the effect of structural architecture on the photovoltage output can be isolated because the 0D and 2D configurations utilize identical active materials and dyes with similar optical bandgaps (∼2 eV), confirming that directional carrier guiding dictated by structural ordering, rather than material chemistry, is the primary driver for achieving a 1 V class photovoltage. Detailed characterizations and electrochemical mechanisms for each photoelectrode configuration are discussed below.

To provide a rigorous basis for analyzing these mechanisms, we first characterized the synthesized cathode materials. Although many Zn-ion battery cathodes undergo complex proton intercalation and activation,^25^ establishing a clear structural baseline is essential for this study. Here we prepared nanocrystalline ZMO with various Mn/Zn molar ratios (2.0, 1.9, and 1.8, denoted as ZM20O, ZM19O, and ZM18O, respectively) whose structures were verified by X-ray diffraction (XRD) profiles (Figure 2a), exhibiting characteristic peaks of a tetragonal spinel-type structure (space group *I*4_1_/*amd*). Scanning electron microscopy (SEM) and energy-dispersive X-ray spectroscopy (EDS) confirmed spherical pristine ZMO particles (Figure S2) with homogeneously distributed Zn and Mn (Figure S3). Inductively coupled plasma optical emission spectroscopy (ICP-OES) confirmed that the compositions matched the targets (Table S1). Rietveld refinement and occupancy analysis based on synchrotron XRD data (Figure S4a–f) demonstrated that all pristine samples maintain a comparable crystallite size of approximately 5 nm and a lattice constant ratio (*c*/*a*) of ∼1.58, confirming the absence of macroscopic phase segregation or uncontrolled structural distortion. These analyses further reveal that Zn and Mn cations primarily occupied the tetrahedral 4*a* and octahedral 8*d* sites, respectively (Tables S2–S6), consistent with the strong preference of Zn cations for the tetrahedral environment in spinel oxide structures.^26,27^ Galvanostatic charge/discharge measurements (Figure 2b) using conventional coin cells with drop-cast composite electrodes showed capacities of 60–80 mAh/g at a current density of 20 mA/g within electrochemically stable potentials;^28^ notably, ZM18O exhibited the lowest initial capacity but the highest cycling stability, consistent with previous studies.^29,30^ While cation deficiencies at the 4*a* site were indicated even for the ZM20O nanoparticles, decreasing the Mn/Zn molar ratio increased vacancies at both sites (Figure 2c). In contrast to stoichiometric ZM20O without 8*d* vacancies, these cation deficiencies in ZM18O activate Zn insertion into both the tetrahedral 4*a* and octahedral 8*d* sites, enabling cooperative migration between these sites and facilitating solid-state diffusion kinetics.^29,31^ Although ZM18O undergoes a slight reduction in crystallite size to approximately 3 nm and *c*/*a* ratio to 1.57 upon initial charging, these structural parameters remain stable during subsequent discharge (Figure S4g–f), supporting its cycling durability and stable charge transfer kinetics.^30^

**Figure 2:**
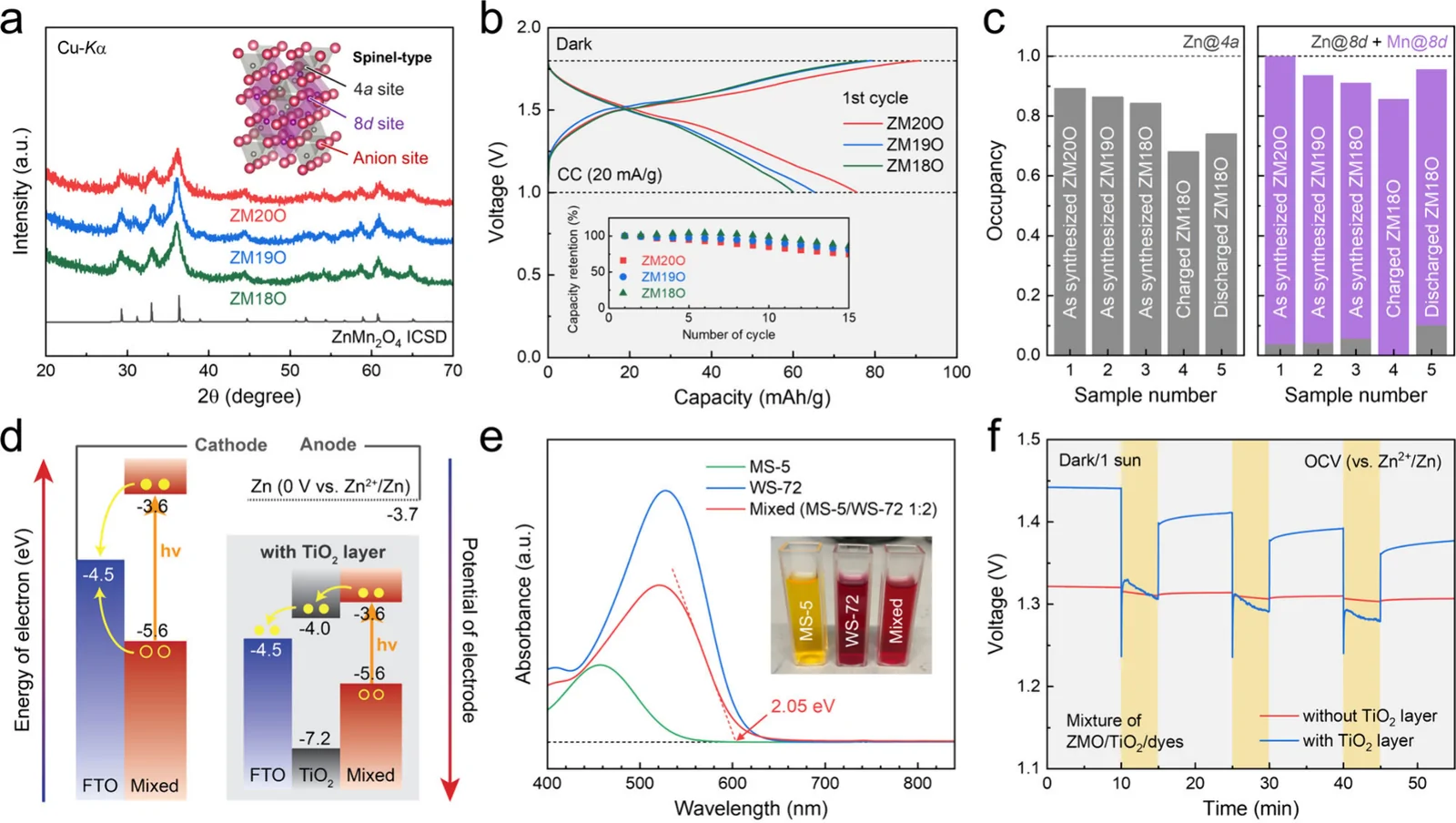
Characterization and limitations of 0D-random mixed photoelectrodes. (a) XRD profiles of the synthesized ZMO with various Mn/Zn molar ratios (2.0, 1.9, and 1.8), denoted as ZM20O, ZM19O, and ZM18O, respectively. The inset illustrates the tetragonal spinel structure (space group *I*4_1_/*amd*) of ZMO, where Zn and Mn cations primarily occupy the tetrahedral 4*a* and octahedral 8*d* sites, respectively. (b) Initial galvanostatic charge/discharge profiles of the synthesized ZMO cathodes in 3 M Zn­(OTf)_2_ aqueous electrolyte. The inset shows the capacity retention over the first 15 cycles. (c) Estimated site occupancy of Zn (gray) and Mn (purple) cations in the as-synthesized materials, as well as ZM18O cathodes after the initial charge/discharge processes. The results were determined via Rietveld refinement of synchrotron XRD data, revealing the cation-deficient nature of the spinel framework. (d) Energy-level diagrams of the mixed photoelectrode without (main) and with (inset) a TiO_2_ blocking layer. The numbers in the figure indicate the electron energy levels relative to the vacuum level. (e) UV–vis absorption spectra of the sensitizing dyes (MS-5, WS-72, and their 1:2 mixture). The inset displays the corresponding dye solutions. (f) OCV responses under dark and 1-sun illumination for the mixed photoelectrodes with and without a spray-coated TiO_2_ blocking layer, demonstrating that the TiO_2_ layer effectively suppresses interfacial charge recombination.

Next, the energy-level diagram (Figure 2d) illustrates the fundamental challenge in the 0D-random mixed configuration: without a TiO_2_ blocking layer, the lack of a selective energy barrier at the FTO interface, alongside carbon-induced trapping and limited optical penetration, promotes carrier back-transfer and significant recombination. The optical properties of the dyes utilized in this work were characterized via UV–vis absorption spectra (Figure 2e). The mixed dyes (MS-5/WS-72 1:2) showed a broad absorption profile spanning the visible spectrum with an energy gap of 2.05 eV, validating the light-harvesting capability of the dye-sensitized mixed composite (see *Mixed photoelectrode* in the Methods section). To evaluate the photoelectrochemical response, OCV profiles were recorded under chopped light illumination (Figure 2f). The dye-sensitized composite (i.e., mixed nanoparticles of ZM20O, TiO_2_, and carbon black) deposited directly on FTO without a discrete TiO_2_ blocking layer exhibited a negligible photovoltage (∼0.01 V), which is within the range of thermal voltage fluctuations (*k*_B_ *T*)/*e* ≈ 0.026 V at room temperature, where *k*_B_, *T*, and *e* denote the Boltzmann constant, absolute temperature, and elementary charge, respectively. This suggests that the observed response reflects the photothermal dissipation as discussed in previous literature.^10,32^ In contrast, the integration of a spray-coated TiO_2_ blocking layer acted as a decisive charge-selective barrier, inducing a sharp photovoltage response. The negative voltage transient immediately after light illumination is presumably associated with short-lived redox reactions, involving species such as water from the electrolyte.^33^ Alternatively, taking advantage of the photothermal effect to accelerate electrochemical kinetics, we evaluated the drop-cast composite electrode within a windowed coin cell.^22^ Under continuous illumination, this “photoenhanced” protocol^11^ resulted in a capacity gain of approximately 20 mAh/g (Figure S5). This photothermal enhancement aligns with the recovery of capacity lost by kinetic limitations observed at higher current densities (Figure S6) or larger particle sizes (Figure S7). Indeed, thermal imaging in the optical cell setup confirmed a temperature rise to ∼36 °C within 10 min under 1-sun illumination (Figure S8), which moderately boosts reaction kinetics;^10^ this is supported by the CV profiles recorded in the same setup (Figure S9). However, intense light illumination can also accelerate parasitic reactions, including electrolyte decomposition or active-material degradation.^34,35^ These results underscore that while photothermal heat can kinetically increase the capacity, a well-defined structured interface is indispensable for converting harvested photons into electrochemical potentials.

To address the recombination issues identified in the 0D mixed configuration, we next investigate 1D type configurations (see Figure 1a) using a p-type Cu_2_O layer to establish a more directed charge-transfer pathway. The energy-level diagram (Figure 3a) suggests that the Cu_2_O layer induces a potential gradient due to band bending,^36^ facilitating the transport of photoexcited holes toward the FTO current collector. In addition, Cu_2_O has been reported as a redox-active conversion-type material involving the formation of metallic Cu in aqueous Zn-ion battery systems,^37,38^ thereby serving as a bifunctional component that both creates photogenerated charges and stores energy. The Gibbs free energy change (ΔG) is estimated to be approximately – 170 kJ/mol for the primary conversion reaction (Cu_2_O + Zn^2+^ + 2e^–^ → ZnO + 2Cu) at ambient temperature, corresponding to a theoretical equilibrium potential of ∼0.9 V vs Zn^2+^/Zn.^37,38^

**Figure 3:**
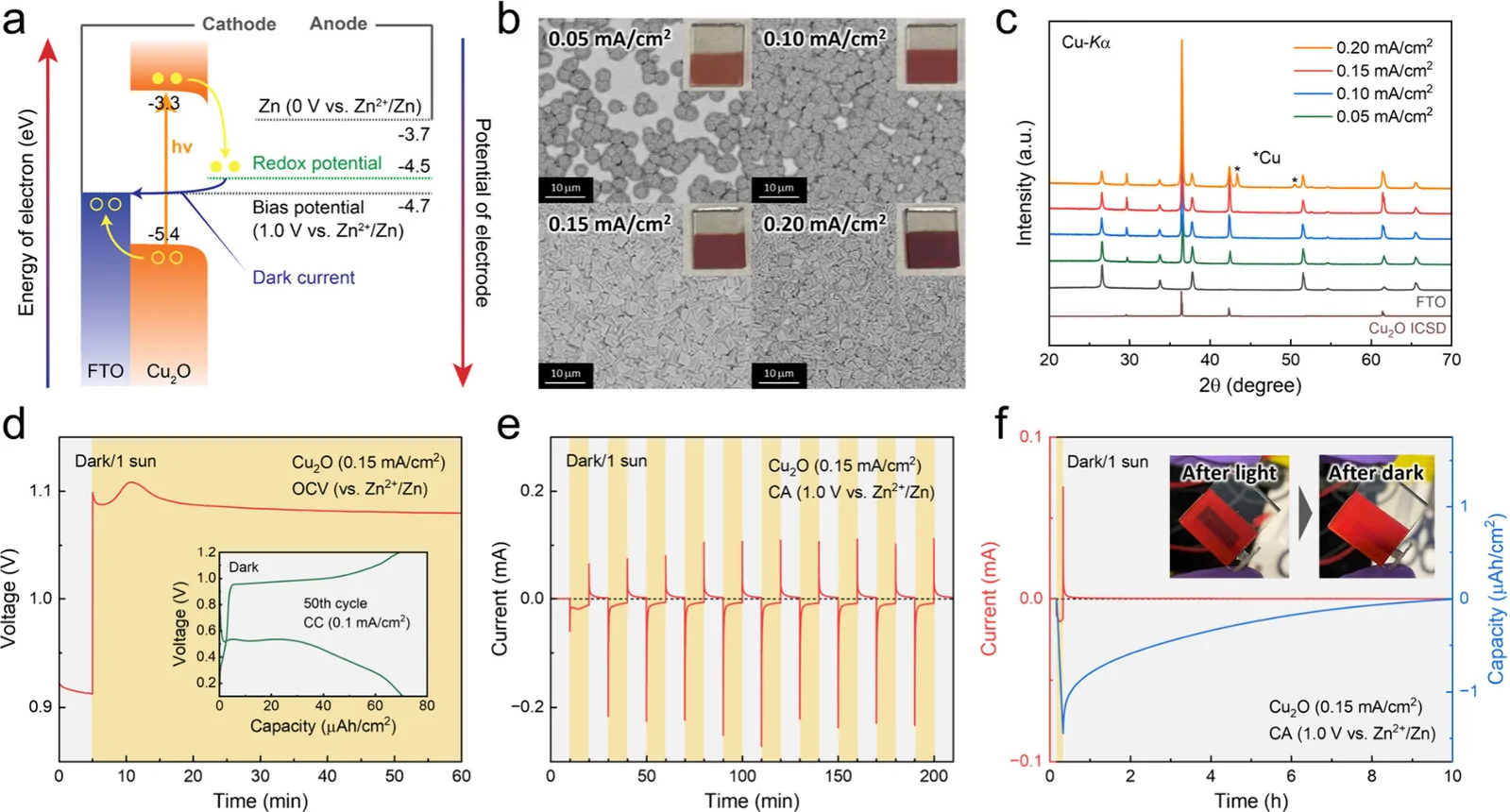
Light-switchable redox operation in 1D-gradient bifunctional film photoelectrodes. (a) Energy-level diagram of the bifunctional Cu_2_O film photoelectrode in a Zn-ion battery system. At a constant bias of 1.0 V vs Zn^2+^/Zn, the redox reaction of Cu_2_O is energetically switchable between photoassisted reduction and dark reoxidation. The numbers in the figure indicate the electron energy levels relative to the vacuum level. (b) SEM images and the corresponding digital photographs of Cu_2_O electrodeposited on FTO at various current densities for 180 min. (c) XRD profiles of the prepared Cu_2_O/FTO substrates. The asterisks indicate an impurity phase of metallic Cu formed at the highest electrodeposition current density (0.20 mA/cm^2^). (d) Photovoltage response of the Cu_2_O/FTO (0.15 mA/cm^2^) under 1-sun illumination in a two-electrode Zn-ion photobattery system with 3 M Zn­(OTf)_2_ electrolyte. The inset shows the dark galvanostatic charge/discharge profiles at the 50th cycle. (e) Reversible switching of photoassisted reduction current and dark oxidation current under intermittent 1-sun illumination at a constant bias of 1.0 V vs Zn^2+^/Zn. (f) Chronoamperometric profiles demonstrating the charge reversibility of the 10 min photoassisted reduction and subsequent dark oxidation at 1.0 V vs Zn^2+^/Zn. The inset pictures show the reversible color change of the Cu_2_O photoelectrode after 10 min illumination and subsequent 10-h dark process.

For these experiments, we first optimized the Cu_2_O films via electrodeposition,^39,40^ varying the deposition current density from 0.05 to 0.20 mA/cm^2^ (see *Bifunctional photoelectrode* in the Methods section). As shown in the SEM images (Figures 3b and S10), higher current densities promoted the growth of dense, cubic crystals.^41^ The thickness of the Cu_2_O film (0.15 mA/cm^2^) was estimated to be approximately 2 μm based on cross-sectional SEM (Figure S11) and profilometry (Figure S12). XRD analysis (Figure 3c) revealed that single-phase Cu_2_O was obtained except at 0.20 mA/cm^2^, where a trace amount of metallic Cu was incorporated, likely due to potential fluctuations during the electrodeposition process (Figure S13). Such an impurity phase was also indicated by the blackish color of the as-deposited film (see the inset of Figure 3b). The bandgap of the prepared single-phase Cu_2_O was evaluated to be approximately 2.1 eV (Figure S14), which is consistent with literature,^42^ and is comparable to those of the dyes used in the 0D and 2D configurations (∼2 eV), making this 1D architecture a direct comparison for their photovoltage outputs. Based on the above observations, the Cu_2_O films electrodeposited at 0.15 mA/cm^2^ were selected for the following experiments.

The photoelectrochemical impact of this 1D-gradient architecture is evidenced by the OCV response under illumination. In contrast to the negligible response of the 0D-random mixed composite, the p-type Cu_2_O electrode exhibited a distinct photovoltage (Figure 3d), representing a net increase of 0.1–0.2 V from the dark state. This behavior is attributed to the shift of the valence band quasi-Fermi level coupled with the flattening of band bending under illumination.^43^ Repetitive OCV tests revealed that the pristine electrode initially delivers a modest photovoltage of approximately 0.05 V (Figure S15), which increases and stabilizes above 0.1 V upon (photo)­electrochemical reduction–oxidation conditioning. In addition, the broad oxidation peak observed a few minutes after illumination was found to be nonreproducible over multiple measurements and might be due to device specific defects (Figure S16). Meanwhile, cyclic voltammetry (Figure S17) and galvanostatic cycling (Figure S18) in the dark showed a stable redox reaction, as also shown in the inset of Figure 3d, with the reduction onset agreeing well with the theoretical value (∼0.9 V) despite polarization-induced delay. To isolate the photothermal effect, black tape was attached to the optical window to absorb the incident light and convert it into heat.^10,11^ This yielded a negligible photovoltage (∼0.01 V), accounting for less than 10% of the total photoresponse (Figure S19). The photoenhanced protocol (Figure S20) further confirmed a photoelectrochemical origin, where the instantaneous potential shift under direct illumination contrasted with the gradual change observed with black tape.^44^ Chronoamperometry (CA) at a bias of 1.0 V (Figures 3e and S21), intermediate between the OCV values in the dark and light conditions, revealed consistent cycles consisting of photoassisted reduction and dark oxidation. The sharp, reversible current spikes observed upon switching the light on and off may arise from transient carrier dynamics mismatch with interfacial reaction kinetics.^45^ Light-driven formation of amorphous zinc oxide and crystalline Cu metal on the Cu_2_O surface was verified by EDS mapping (Figure S22), XRD analysis (Figure S23), and Raman spectroscopy (Figure S24), where subtle peak variations can be attributed to the presence of trace CuO species on the film surface.^46,47^ This demonstrates that conversion-type battery reactions are taking place under illumination. A similar reaction was observed when Zn metal was thermally deposited on Cu_2_O; specifically, the OCV of the fabricated cell gradually increased under the illumination (Figure S25), accompanied by identical structural change in XRD (Figure S26). A long-term CA test starting with a 10 min illumination period followed by a discharge in dark conditions (Figure 3f) demonstrated high charge reversibility, where the higher current during photoassisted charging, relative to the discharge current, stems from the enhanced conductivity via photogenerated carriers. Importantly, the reversibility of this conversion process was visually confirmed by the optical color transition, where the blackish appearance developed upon a 10 min illumination completely restored to the original orange hue after 10 h of dark reoxidation (Figure 3f, insets), quantitatively agreeing with the extracted charge.

To investigate the influence of film morphology, we further prepared thinner and flatter Cu_2_O films electrodeposited on a 3 nm Au layer (Figure S27), which is thin enough to be transparent (Figure S28).^42^ Despite their preferred orientation in XRD (Figure S29), these films exhibited a lower photovoltage than the Au-free electrodes (Figure S30), suggesting that the relatively rougher surface was more advantageous for effective light-harvesting, while reduced light penetration, optical reflection, and parasitic absorption by the Au layer also contribute to the lower photovoltage. Although forming a p–n junction by introducing ETLs such as Ga_2_O_3_ and TiO_2_ could further enhance the photovoltage,^48,49^ such an interlayer would inevitably impede the direct electrochemical interaction between Cu_2_O and the electrolyte, thereby sacrificing its bifunctionality.

While the 1D-gradient bifunctional film architecture significantly improves the photovoltage compared to the 0D-random mixed configuration, the absolute potential remains limited by the intrinsic band alignment of the active material and the electrolyte interface.^43^ This limitation can be avoided by a 2D-interfacial layer-by-layer architecture (Figure 1a), which integrates the ZMO cathode into a DSSC framework to maximize potential confinement.^50^ The detailed fabrication process for each layer is described (see *Layer-by-layer photoelectrode* in the Methods section), where defective ZM18O was employed to prioritize cycling stability over initial capacity. Cross-sectional SEM and EDS elemental mapping confirmed the stacked layer-by-layer structure consisting of FTO, a TiO_2_ blocking layer, mesoporous TiO_2_ with dye, the ZMO composite, and a carbon back contact (Figures 4a,b and S31). Sharp interfacial boundaries without obvious intermixing were observed between adjacent layers. As illustrated in the simplified energy-level diagram (Figure 4c), this configuration establishes a unidirectional charge-transport pathway: photoexcited electrons are extracted via the TiO_2_ ETL, while holes are injected into the ZMO storage layer through the poly­(3,4-ethylenedioxythiophene) (PEDOT) HTL.^51^ The work function of PEDOT^52^ aligns with the ZMO potential, ensuring efficient hole injection and suppressed charge recombination. Furthermore, the dye mixture includes WS-72, which possesses a sufficiently low HOMO level^53^ to energetically enable the oxidation of the ZMO cathode.

**Figure 4:**
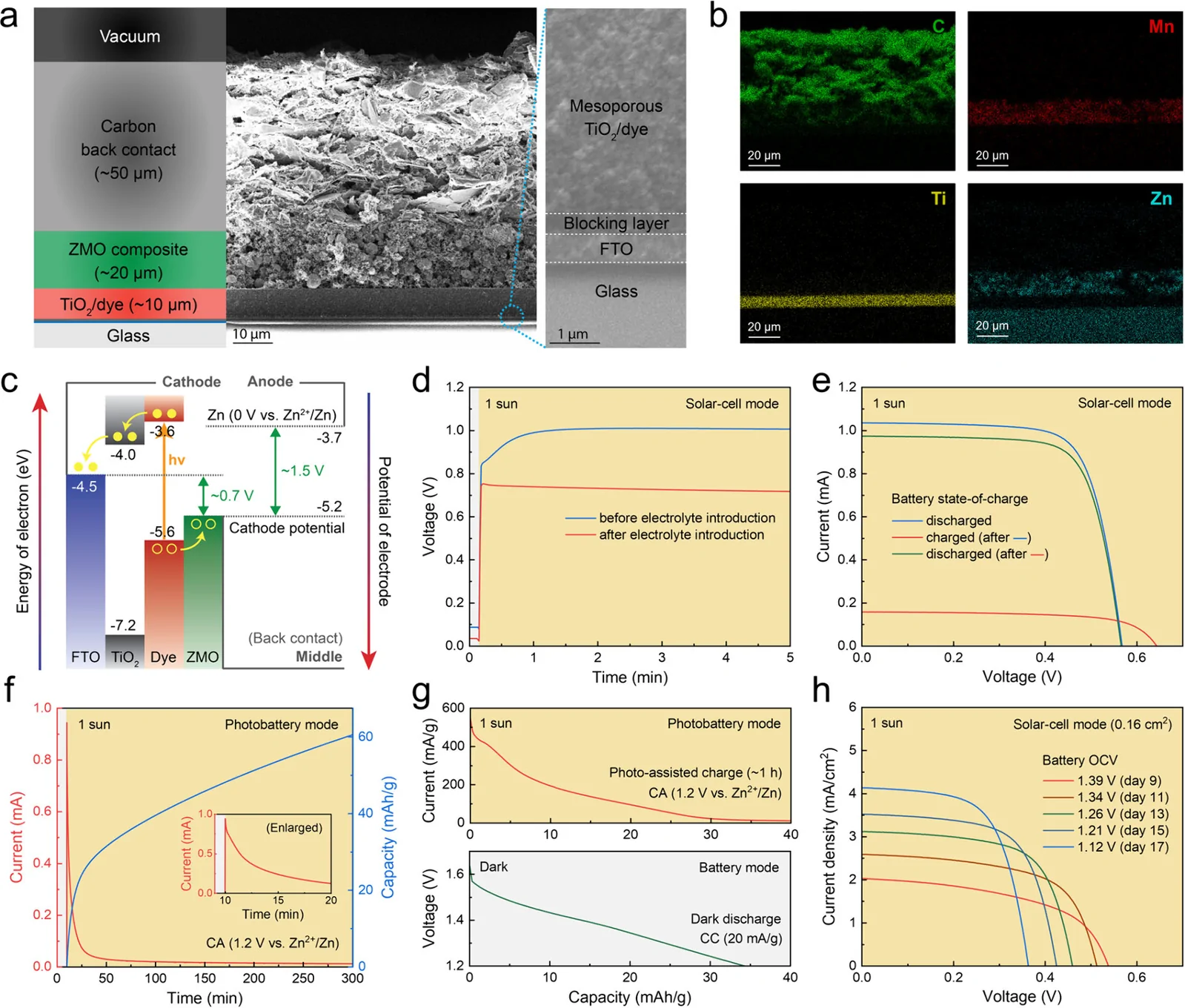
Three-electrode operation of 2D-interfacial layer-by-layer photoelectrodes. (a) Cross-sectional SEM image of the fabricated layer-by-layer photoelectrode with an enlarged view (right). The glass substrate, FTO, TiO_2_ blocking layer, mesoporous TiO_2_/dye, ZMO composite, and carbon back contact are sequentially stacked from bottom to top. (b) Cross-sectional EDS elemental mappings (C, Ti, Mn, and Zn) for the layer-by-layer photoelectrode, clearly resolving the spatial distribution of each functional layer. (c) Simplified energy-level diagram of the three-electrode layer-by-layer Zn-ion photobattery system. Photoexcited electrons are injected into the conduction band of TiO_2_ and collected by FTO, while generated holes travel through the PEDOT layer to drive the oxidative charging of the defective ZMO cathode. The numbers in the figure indicate the electron energy levels relative to the vacuum level. (d) Photovoltage profiles under 1-sun illumination before and after the introduction of 3 M Zn­(OTf)_2_ aqueous electrolyte. The stabilized photovoltage of ∼0.7 V observed thereafter aligns with the potential difference between the FTO/TiO_2_ contact and the ZMO cathode. (e) *I*–*V* characteristics in solar-cell mode under 1-sun illumination (using the total active area of approximately 1 cm^2^) at different SOC of the ZMO cathode. The reversible change in photocurrent indicates that the driving force for charge separation is modulated by the cathode potential. (f) Photoassisted charging behavior of the ZMO cathode at a constant bias of 1.2 V vs Zn^2+^/Zn in photobattery mode. The inset shows the enlarged current response immediately after illumination. (g) Chronoamperometric curve during the photoassisted charge (∼1 h) at 1.2 V vs Zn^2+^/Zn in photobattery mode (upper) and the subsequent galvanostatic (20 mA/g) dark discharge profile in battery mode (lower). (h) Stability test showing the correlation between *J*–*V* curves (measured using a defined active area of 0.16 cm^2^) and battery OCV monitored over 17 days after the electrolyte introduction.

In solar-cell mode, the device achieved an OCV of ∼1.0 V under 1-sun illumination (Figure 4d), with an active area of ∼1.0 cm^2^. While this photovoltage remained stable for 1 week (Figure S32), the electrolyte introduction led to a concomitant reduction in the solar-cell OCV to ∼0.7 V (Figure S33). This value aligns well with the energy offset between the ZMO cathode (∼1.5 V vs Zn^2+^/Zn, equivalent to ca. −5.2 eV vs vacuum) and the FTO/TiO_2_ contact potential, with the work function of FTO set as a reference point (typically ca. −4.5 eV vs vacuum). This indicates that the ZMO/back-contact potential was pinned to the redox reaction in the electrolyte, as shown in Figure 4c. Notably, while the 0D and 1D systems measure photovoltage in a two-electrode setup directly against the metallic Zn counter electrode, the insulating nature of the 2D layer-by-layer system requires a middle back contact. Nevertheless, operating the 2D system in two-electrode photobattery mode (see Figure 1d) yields a comparable photovoltage (Figure S34), justifying the direct comparison of photovoltage scales across the different architectures. Dark charge/discharge precycling was performed prior to the following experiments (Figure S35), which slightly reduced the photovoltage to ∼0.6 V. The subsequent *I*–*V* characteristics (Figure 4e) further demonstrate the functional synergy between the solar-cell performance and battery state-of-charge (SOC). A dynamic decrease in photocurrent was observed as the ZMO cathode reached a higher SOC at a more positive potential. This diminishes the driving force for hole injection from the dye molecules to the cathode via the HTL due to the narrowed energy offset, as reported in related fields.^54,55^ Notably, the photocurrent response was restored upon discharging the ZMO cathode, confirming the electrochemical reversibility of the integrated system.

The photoassisted charging performance of the three-electrode layer-by-layer system (Figure 4f) was evaluated in photobattery mode. Under a constant bias of 1.2 V vs Zn^2+^/Zn, the device exhibited a substantial photoassisted charging current, yielding a capacity of ∼60 mAh/g within 5 h. The photocurrent immediately following illumination was around 1.0 mA, subsequently decreasing to half within several minutes, as shown in the inset. Such a decrease in photocurrent can be attributed to the cathode potential shift (Figure S36); as photoassisted charging proceeds and the SOC increases, the cathode potential progressively shifts upward to approach the energy level of the HTL, which diminishes the energetic driving force as stated before (Figure S37). The obtained capacity is comparable to the charge/discharge capacity observed in the standard cycling experiments in dark conditions (Figure 2b), which is reasonable because the sum of the photovoltage and the external bias (1.2 V) approximates the upper cutoff potential (1.8 V) used in the galvanostatic cycling. The sequence of chronoamperometric photoassisted charging (∼1 h) and constant-current dark discharging (Figure 4g) confirms the charge reversibility of the layer-by-layer photoelectrode under operational conditions. Following photoassisted charging of ∼40 mAh/g (upper panel), the subsequent dark discharge (20 mA/g down to 1.2 V vs Zn^2+^/Zn) yielded a discharge capacity of ∼34 mAh/g (lower panel), corresponding to a Coulombic efficiency of ∼85%. The initial discharge potential of ∼1.6 V vs Zn^2+^/Zn, which is markedly higher than the external bias voltage, provides clear evidence of the photovoltaic effect under illumination. Based on the energy output stored above the applied bias voltage, the overall solar-to-chemical energy conversion efficiency was evaluated to be ∼0.015% (see Note S1 for detailed calculations).^11^ This modest efficiency mainly originates from the photocurrent decay during photocharging. Similar photoassisted charging and discharging behavior was also observed when using ZM20O as the cathode material (Figure S38). To evaluate the intrinsic static interfacial compatibility of the monolithic integrated architecture without secondary failure modes such as dendrite-induced short circuits during battery cycling (Table S7), the long-term stability of the device was monitored over several weeks through minimal nondestructive solar-cell *J*–*V* sweeps and battery OCV tracking (Figure 4h) using a defined illumination area (0.16 cm^2^). Notably, the battery OCV was well preserved before and after each *J*–*V* measurement, demonstrating that the photovoltaic and energy storage components maintain high integrity over long-term contact. A gradual decrease in battery OCV from day 9 onward (see the legends in Figures 4h and S39) suggests mild self-discharge, a common issue in aqueous battery systems.^56^ The *J*–*V* curves evolved in correlation with the battery OCV, consistently supporting the discussion above.

In summary, we have demonstrated that the structure of photoelectrodes is a decisive factor in achieving light-assisted charging of photobatteries. Randomly dispersed 0D electrode configurations are easy to manufacture, but in our experiments these mixtures of dyes, extraction and battery materials suffer from charge recombination, yielding a negligible photovoltage of ∼0.01 V. In a single layer 1D configuration using a Cu_2_O bifunctional film, charge carriers are successfully separated to create an output voltage in the order of 0.1 V. Final 2D layer-by-layer design, integrating a solid-state DSSC for electron–hole pair generation along with charge transport layers and an optimized spinel ZMO cathode, increased the photovoltage output by an order of magnitude to approximately 1 V under 1-sun illumination. This dramatic performance enhancement is attributed to the establishment of rectified charge-transfer pathways and effective potential confinement at well-defined interfaces. This systematic evolution establishes foundational design guidelines for tailoring photobattery performance to specific functional requirements.

## Supplementary Material



## Notes

Experimental details including materials synthesis, cell assembly, photoelectrode fabrication, a solar simulator spectrum, SEM/EDS images, ICP-OES results, XRD and Rietveld refinement profiles, OCV measurements, galvanostatic charge/discharge curves, a thermal camera image, cross-sectional SEM/EDS mapping, profilometry, voltage profiles during electrodeposition, Tauc plots, CA and CV curves under dark/light conditions, Raman spectra, and *I*–*V* curves (PDF)
