# Supplementary material for: Photobattery Architectures to Suppress Charge Recombination
Source: Nano Lett. 2026 Oct 13;26(42):14520–8. doi: 10.1021/acs.nanolett.6c02373 (PMC13721156; doi:10.1021/acs.nanolett.6c02373)
Supplement: Supplementary file 1 [file nl6c02373_si_001.pdf]

Supporting Information for

Photobattery Architectures to Suppress Charge Recombination

Kohei Shimokawa,[1,2,3,*] Byung-Man Kim,[1] Arvind Pujari,[1,4] Hooman Abbasi,[1] Tetsu Ichitsubo,[3] Michael De Volder[1,*]

[1]Institute for Manufacturing, Department of Engineering, University of Cambridge, Cambridge CB3 0FE, UK
[2]Frontier Research Institute for Interdisciplinary Sciences, Tohoku University, Sendai, 980-8578, Japan
[3]Institute for Materials Research, Tohoku University, Sendai, 980-8577, Japan
[4]Cavendish Laboratory, Department of Physics, University of Cambridge, Cambridge CB3 0HE, UK

[*]Correspondence: kohei.shimokawa.b7@tohoku.ac.jp (K.S.); mfld2@cam.ac.uk (M.D.V.)

Methods

Synthesis

Zn–Mn oxide (ZMO) nanoparticles were synthesized via inverse coprecipitation [1]. In a typical procedure, a 0.35 M Na_2CO_3 (Fisher Chemical) aqueous solution (200 mL) was prepared as the precipitant. Separately, a mixed nitrate precursor solution (100 mL) was prepared by dissolving $Zn(NO_3)_2 \cdot 6H_2O$ (Alfa Aesar) and $Mn(NO_3)_2 \cdot 4H_2O$ (Merck) in deionized (DI) water. The concentration of $Zn(NO_3)_2$ was fixed at 0.08 M, while the concentration of $Mn(NO_3)_2$ was adjusted (*e.g.*, 0.16 M for ZM20O) according to the target stoichiometric ratio. Both solutions were stirred at approximately 500 rpm and heated to ~70 °C. Once the temperatures stabilized, the mixed nitrate solution was added dropwise into the Na_2CO_3 solution over a period of approximately 15 min. During this process, the stirring rate was gradually increased to 750 rpm to maintain a homogeneous suspension. The mixture was then aged at 70 °C for an additional 30 min under continuous stirring. The resulting precipitate was collected by vacuum filtration and washed thoroughly with 300 mL of hot DI water (~70 °C) to remove residual sodium salts. The obtained powder was dried in a vacuum oven at 60 °C overnight. The dried precursor (typically ~2.5 g) was ground for 15 min using a mortar and pestle, followed by calcination at 350 °C for 4 h in a muffle furnace under ambient air. Finally, the calcined product was ground again for 15 min to yield the ZMO nanoparticles.

Drop-casting

Composite electrodes were prepared by a drop-casting method onto carbon paper (CP) substrates. Prior to use, the CP (TGP-H-60, Toray) was punched into disks with a diameter of 10 mm. These CP disks were subjected to UV-O_3 treatment for 20 min to remove organic contaminants and to improve their surface hydrophilicity. The slurry was prepared by mixing the as-synthesized ZMO nanoparticles, Super-P, and polyvinylidene fluoride (PVdF) dissolved in N-methyl-2-pyrrolidone (NMP) in a weight ratio of 8:1:1. The mixture was homogenized using a planetary mixer (ARM-310, THINKY) for 20 min. Subsequently, 20 μL of the resulting slurry was drop-cast onto the prepared CP disk. The electrodes were initially dried on a hot plate at ~120 °C for several hours, followed by vacuum drying at 120 °C overnight to ensure complete removal of the solvent.

Coin cells

The galvanostatic charge/discharge was performed using 2032-type coin cells. The average mass loading of the cathode active material was approximately 2 mg/cm². Polished Zn foil and a glass fiber filter (GF/A, Whatman) served as the anode and separator, respectively, with 3 M $Zn(OTf)_2$ (Sigma-Aldrich) aqueous electrolyte. For the photo-enhanced experiments, windowed coin cells were employed, in which the center of the positive can was perforated and sealed with a glass window to allow for light illumination onto the cathode. Cells were then allowed to rest for several hours to ensure electrode wetting.

Mixed photoelectrode

The mixed photoelectrodes were fabricated on FTO substrates (see the Layer-by-layer photoelectrode section for details including the spray coating of the TiO_2 layer). The slurry was prepared by mixing the ZMO nanoparticles, anatase TiO_2 (< 25 nm, Sigma-Aldrich), Super-P, and PVdF (dissolved in NMP) in a weight ratio of 4:4:1:1. The slurry was coated onto the FTO substrate and dried on a hot plate at ~120 °C. For dye sensitization, a mixed dye solution was prepared utilizing WS-72 and MS-5 dyes (Dyenamo) with chenodeoxycholic acid (CDCA, Sigma-Aldrich) as an anti-aggregation agent. Specifically, WS-72 and CDCA were dissolved in a toluene/ethanol (1:4 v/v) mixture, while MS-5 and CDCA were dissolved in a tert-butanol/acetonitrile (1:1 v/v) mixture. These solutions were mixed in a 2:1 volume ratio. The electrodes were immersed in the mixed dye solution for 16 h in the dark. Cells were constructed by sealing the sensitized photoelectrode and a polished Zn foil anode using Surlyn spacers (Solaronix) at ~120 °C. Then, a 3 M $Zn(OTf)_2$ aqueous electrolyte was injected into the cell, and the injection holes were sealed with a cover glass.

Bifunctional photoelectrode

The Cu_2O films were prepared via galvanostatic electrodeposition [2]. The deposition bath was prepared by dissolving 0.2 M $CuSO_4$ (Fisher Chemical), 0.5 M K_2HPO_4 (Sigma-Aldrich), and 3 M lactic acid (Sigma-Aldrich) in DI water. The pH was adjusted to 12 by the dropwise addition of an aqueous 2 M KOH solution. FTO substrates were cleaned via ultrasonication in acetone, ethanol, and DI water, followed by UV-O_3 treatment for 30 min. A Ti foil was used as the counter electrode. The active area was defined to be 3.0 cm^2 using PTFE masking tape. Electrodeposition was performed in a water bath at 30 °C using an Autolab (PGSTAT302N) potentiostat. Various cathodic current densities (0.05, 0.10, 0.15, and 0.20 mA/cm^2) were investigated for a constant deposition time of 180 min, and the Cu_2O films prepared at 0.15 mA/cm^2 were selected for detailed characterization.

Layer-by-layer photoelectrode

<u>(1) Substrate preparation and spray coating</u>

The FTO glass substrates (TCO22-7/LI, Solaronix) were partially etched using Zn powder and 12% HCl, followed by sequential ultrasonication in acetone, ethanol, and DI water. A TiO_2 blocking layer (approximately 200 nm thick) was deposited via spray pyrolysis. A precursor solution containing titanium diisopropoxide bis(acetylacetonate) and isopropanol (1:10 v/v) was sprayed onto the substrates maintained at 500 °C. The electrodes were then annealed at 500 °C for 1 h. After cooling to room temperature, the films were treated with a 40 mM $TiCl_4$ aqueous solution at 75 °C for 35 min using an oven (LHT 6/60, Carbolite) to minimize pinholes. The obtained substrates were rinsed with DI water and dried under a stream of compressed air.

(2) Mesoporous layer and dye sensitization

A mesoporous TiO_2 layer was formed by screen-printing a TiO_2 paste (30NR-D, Greatcell Solar Materials), followed by sintering at 500 °C for 1 h in a tube furnace (TS3 12/60/600, Carbolite) under a continuous air flow (~100 sccm). The aforementioned $TiCl_4$ treatment and the subsequent sintering process were repeated to optimize the film quality. The resulting electrodes were immersed in the mixed dye solution (MS-5/WS-72 1:2) for 16 h in the dark.

(3) Photo-polymerization and post-treatment

The hole-transporting PEDOT layer was prepared via *in-situ* photo-electrochemical polymerization. The monomer solution consisted of 10 mM bis-EDOT (ChemShuttle), 100 mM LiTFSI (Sigma-Aldrich), and 100 mM BMIM-TFSI (Sigma-Aldrich) in acetonitrile. Photo-polymerization was performed by applying a constant potential (0.40 V vs. SHE) under white LED illumination until a total charge of 0.054 C was reached. Post-treatment was conducted by spin-coating a solution of 0.02 M LiTFSI (Sigma-Aldrich) and 0.2 M 4-tert-butylpyridine (Sigma-Aldrich) in acetonitrile at 2000 rpm for 30 s, followed by drying at 75 °C.

(4) Blade coating and device assembly

For the battery cathode, a slurry containing the active material (ZMO), Super-P, CMC, and SBR (in a weight ratio of 80:12:4:4) was blade-coated onto the prepared photoelectrode. A commercial carbon paste (DM-CAP-4703S, Dycotec Materials) was then applied as a back contact and dried at 75 °C. The cell was completed by sealing the photoelectrode with a Zn foil anode (attached to a glass substrate) and a PTFE membrane separator (Millipore) using Surlyn spacers (Solaronix) at ~120 °C. Finally, a 3 M $Zn(OTf)_2$ aqueous electrolyte was injected through the pre-drilled holes, which were subsequently sealed with a cover glass.

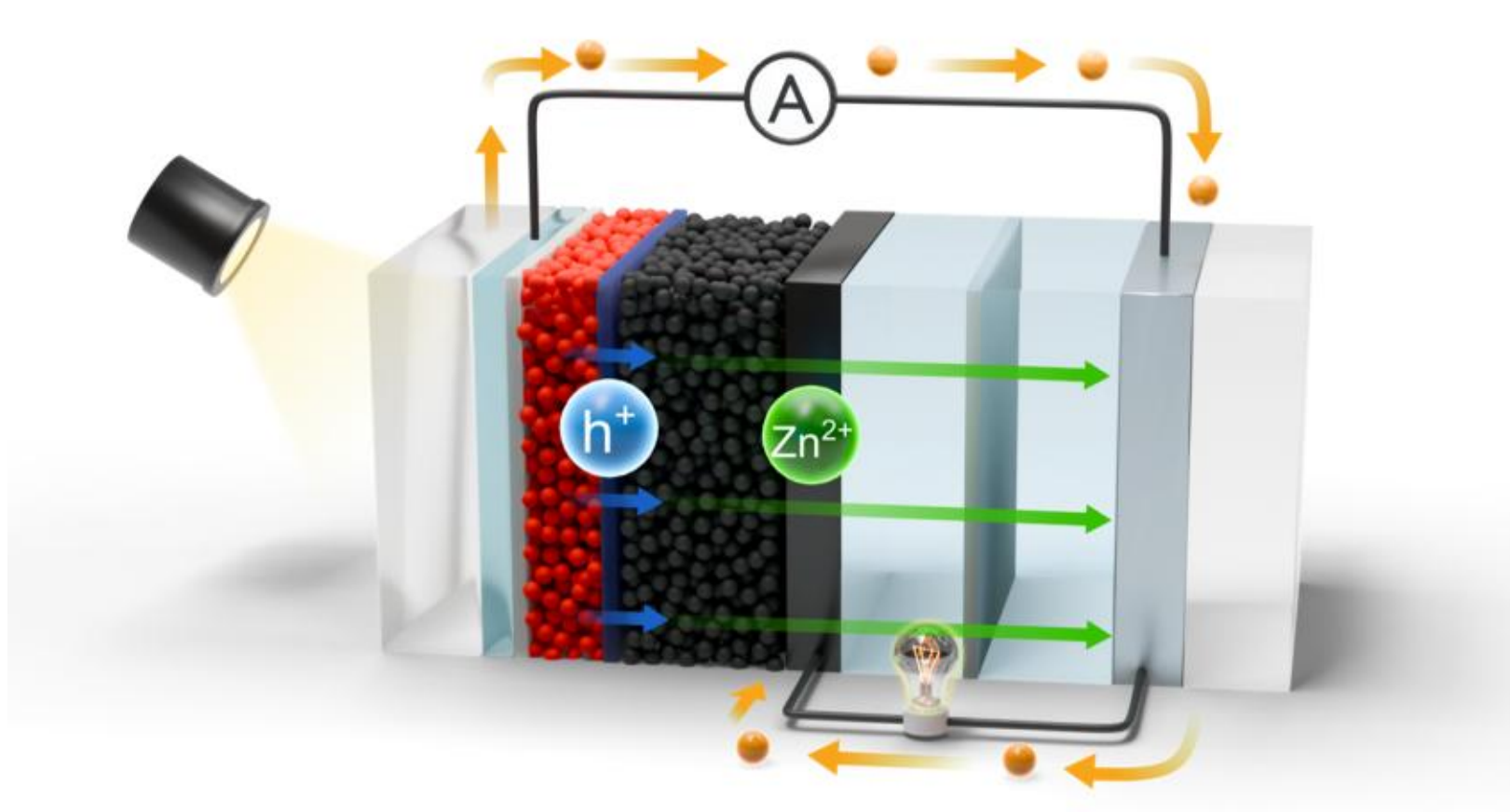

From left:
- Glass (~1 mm)
- FTO (~500 nm)
- TiO_2 blocking layer (~200 nm)
- Mesoporous TiO_2/dye (~10 µm)
- Hole transport layer (~100 nm)
- Battery composite (~20 µm)
- Carbon back contact (~50 µm)
- PTFE separator (~50 µm)
- Zn foil (~10 µm)
- Glass (~1 mm)

(Photo)electrochemical measurements

Electrochemical characterizations, including constant current (CC) charge/discharge cycling, chronoamperometry (CA), cyclic voltammetry (CV), and open-circuit voltage (OCV) measurements, were performed using multichannel potentiostat/galvanostat instruments (VMP3 and BCS-805, BioLogic). 1-sun illumination was provided by a solar simulator (LSH-7320, Newport). For the three-electrode configuration, photo-assisted charging and subsequent dark discharging were conducted independently by reconfiguring the terminal connections for each process. The light-active area of the photoelectrode was defined as either ~1 cm^2 or 0.16 cm^2, depending on the experiment, using 3D-printed aperture fixtures that ensure consistent illumination geometry across all measurements. Thermal imaging under 1-sun illumination was performed using a thermal camera (FLIR C3-X) within the same 3D-printed aperture setup.

Characterization

Crystal structures were investigated by XRD using a D8 DISCOVER (Bruker) at the Maxwell Centre, University of Cambridge, with a Cu *K*α source. Synchrotron XRD data were collected at the Diamond Light Source from powder samples sealed in capillary tubes. Rietveld refinement was performed using the RIETAN-FP program [3]. The Mn/Zn ratio was fixed to the ideal composition based on the elemental analysis results. For the samples after charge/discharge, the Mn occupancy was fixed, while the Zn occupancy was optimized. Visualization of the crystal structure was carried out using the VESTA software [4]. Elemental analysis was conducted via ICP-OES at the Analytical Research Core for Advanced Materials (ARCAM), Institute for Materials Research, Tohoku University. Optical absorption spectra were recorded using a Lambda 750 (PerkinElmer) UV–vis spectrophotometer. Surface and cross-sectional morphologies were characterized by SEM/EDS using Phenom Pro (Phenom), Helios NanoLab 650 (FEI), and LEO 1530 VP (ZEISS) instruments. Film thicknesses were measured using a Dektak XT (Bruker) profilometer at the Maxwell Centre, University of Cambridge. Raman spectra were obtained at the Maxwell Centre using an inVia confocal Raman microscope (Renishaw) with a 633 nm excitation laser. Thermal evaporation of Zn and thin Au films was performed using an AUTO 306 (Edwards) evaporator. Thermodynamic calculations of Gibbs free energy changes were carried out using HSC Chemistry 5.0 software.

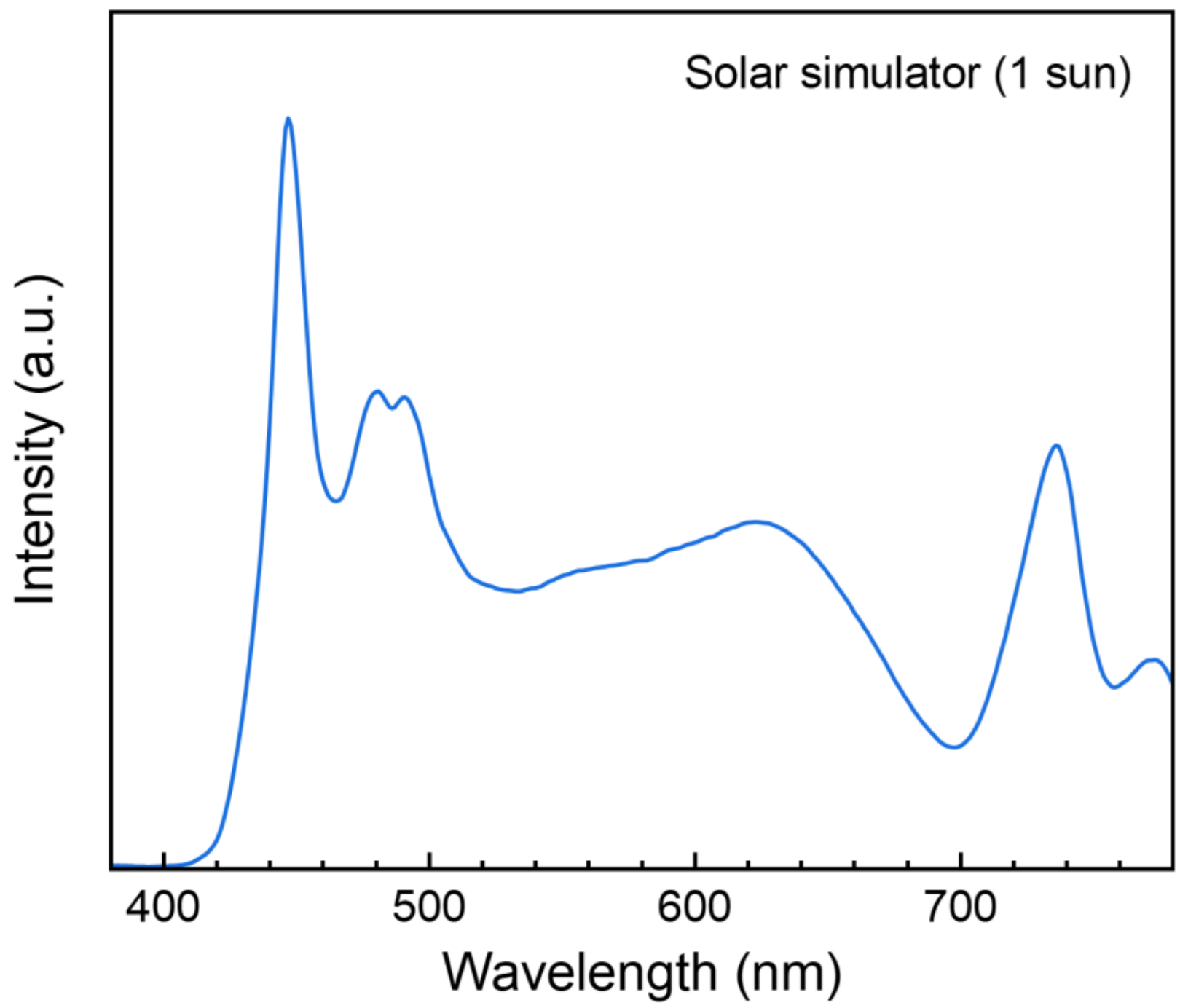

Fig. S1 | Emission spectrum of the solar simulator. The relative light intensity as a function of wavelength was measured using a photometer operating within the visible region. The same light source was employed throughout this work unless otherwise noted.

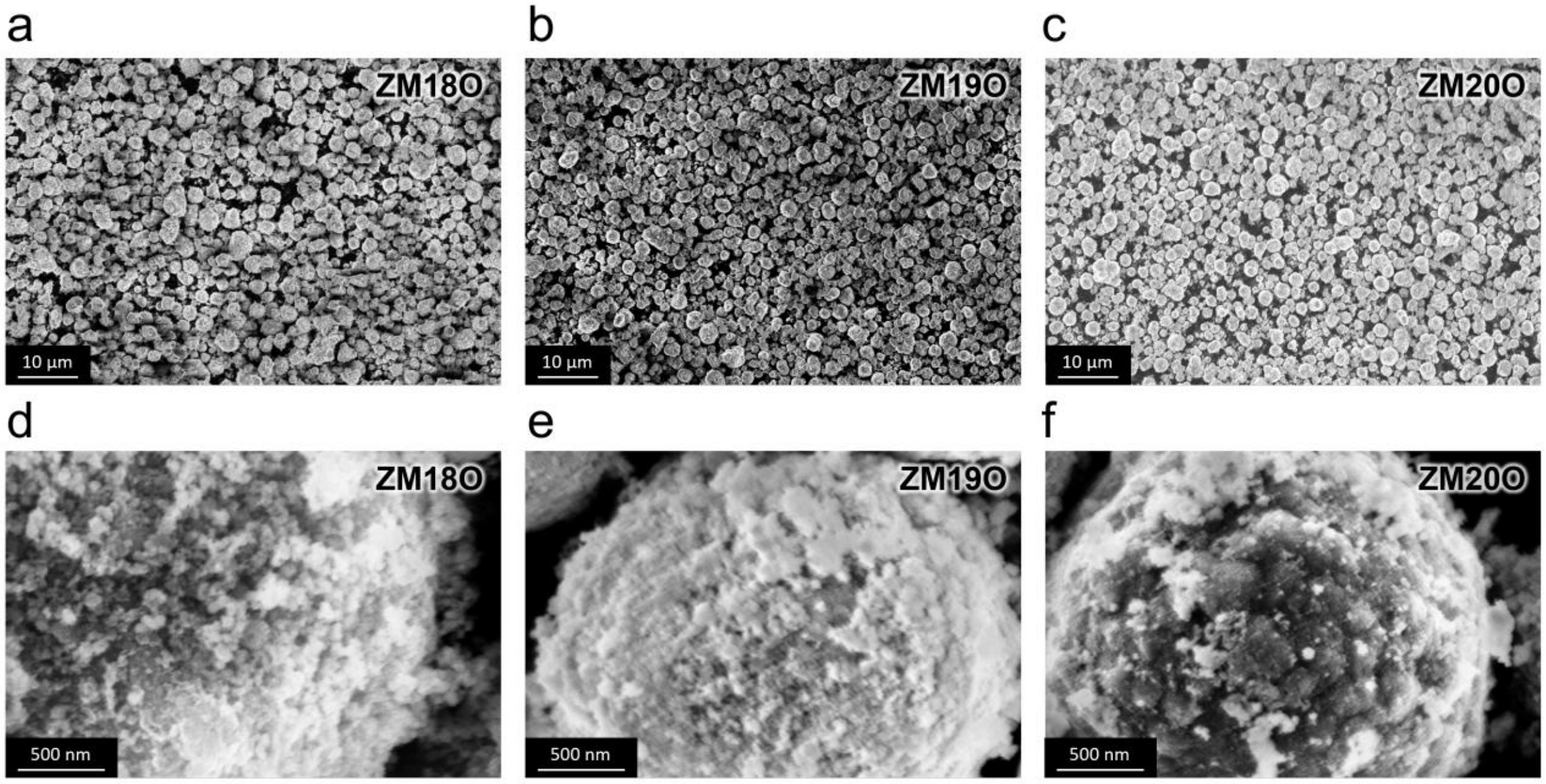

Fig. S2 | SEM images of the synthesized ZMO samples. Low-magnification overview images of (a) ZM18O, (b) ZM19O, and (c) ZM20O powders exhibit a homogeneous, spherical shape. High-magnification images show the surface morphology of individual secondary particles for (d) ZM18O, (e) ZM19O, and (f) ZM20O.

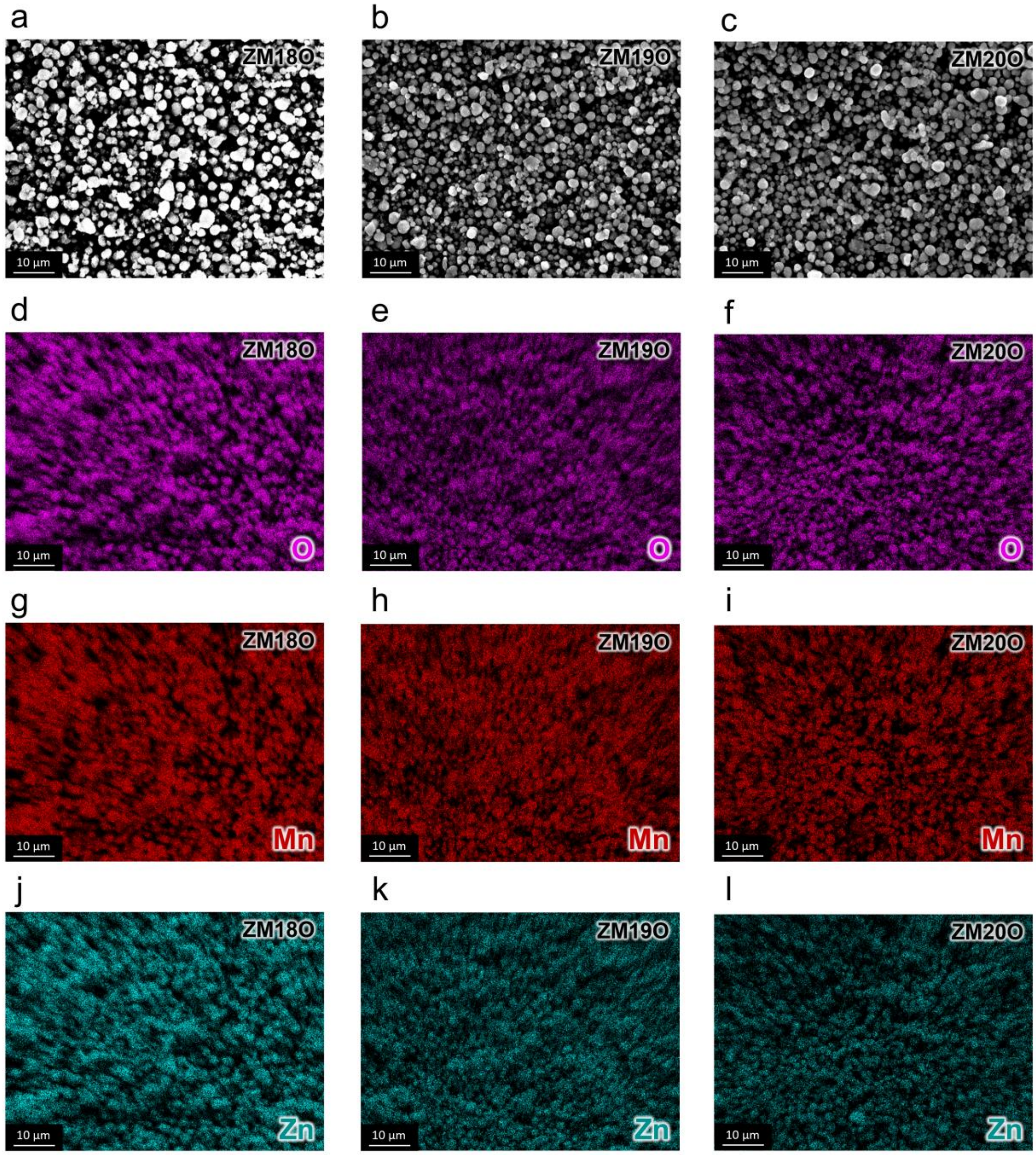

Fig. S3 | SEM and corresponding EDS elemental mapping of the synthesized ZMO samples. (a–c) SEM images and EDS mapping of (d–f) O, (g–i) Mn, and (j–l) Zn were carried out for the synthesized ZM18O (left), ZM19O (middle), and ZM20O (right) powders, respectively.

Table S1 | ICP-OES analysis for synthesized ZMO. Molar ratios of Mn/Zn for the three different ZMO samples were evaluated by ICP-OES analysis. The results are in good agreement with the target compositions.

	ZM20O	ZM19O	ZM18O
Target (Mn/Zn)	2.00	1.90	1.80
ICP-OES (Mn/Zn)	2.03	1.91	1.80

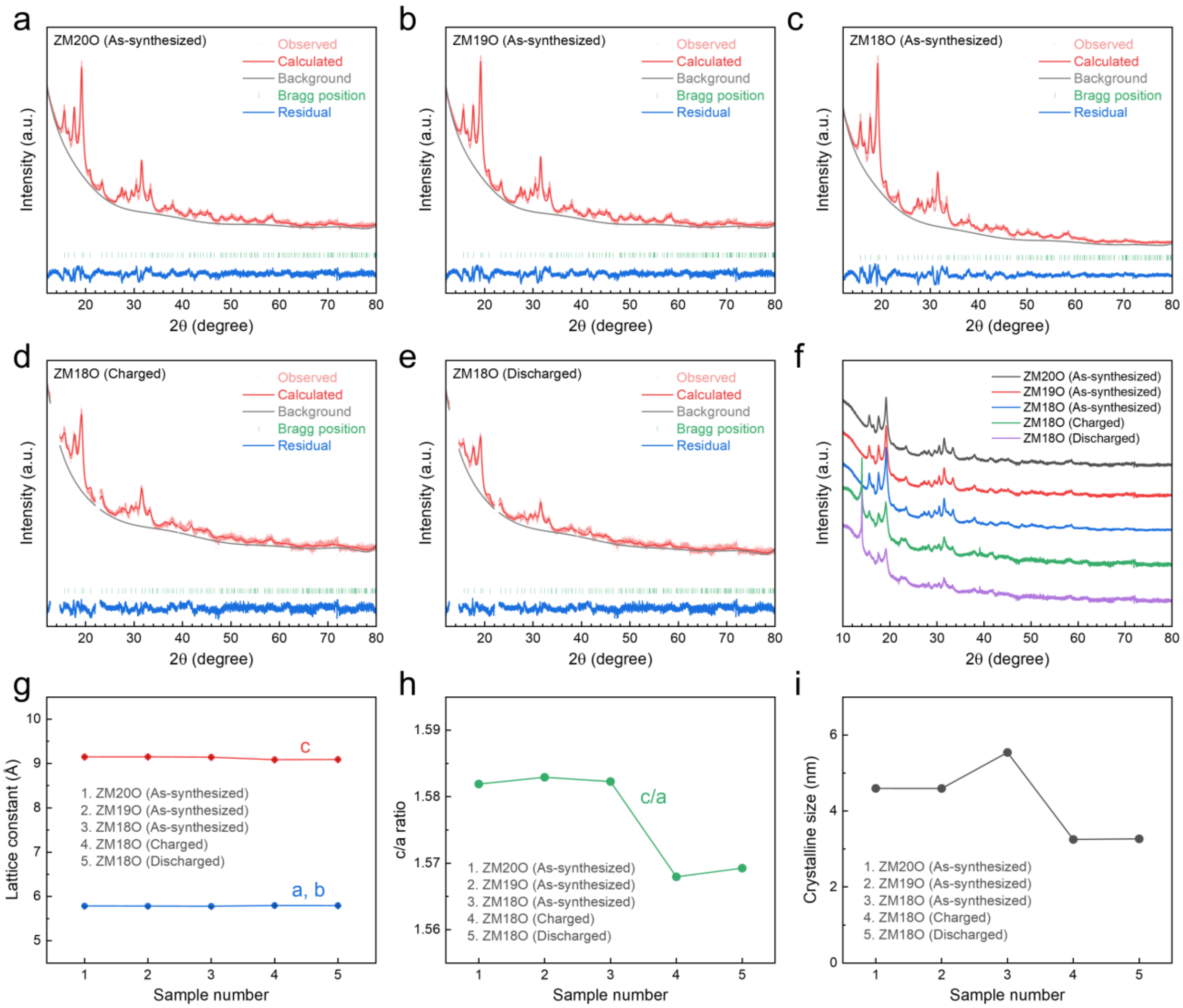

Fig. S4 | Rietveld refinement of ZMO samples. Rietveld refinement was carried out for synchrotron XRD data of the as-synthesized (a) ZM20O, (b) ZM19O, (c) ZM18O, and ZM18O after first (d) charge and (e) discharge, where the diffraction peaks from subsidiary battery components were excluded from the fitting. (f) XRD patterns for all samples are shown for comparison. Structural parameters were obtained from the refinement: (g) lattice constants *a* and *c*, (h) *c*/*a* ratio, and (i) crystallite size. In the fitting, the following constraints were applied: (i) the Mn/Zn molar ratio was fixed to the ideal value, consistent with the elemental analysis; (ii) the oxygen occupancy at the 16*h* site was fixed to 1; (iii) for the charged and discharged samples, only the Zn occupancy was optimized based on the as-synthesized ZM18O results. The estimated charge and discharge capacities from the Rietveld refinement were 66 and 63 mAh/g, respectively, which are consistent with the galvanostatic cycling profiles shown in the manuscript.

Table S2 | Crystal structure of as-synthesized ZM20O. Crystal structure parameters determined by the Rietveld refinement for the as-synthesized ZM20O sample, where *g* and *B* indicate the occupancy and the Debye–Waller factor, respectively. A number in bracket indicates the standard deviation in the last digit.

ZM20O	Element	Site	*g*	*B*
As synthesized	Zn	4*a*	0.892(7)	0.365 [c]
	Zn	8*d*	0.036 [a]	0.365 [c]
	Mn	8*d*	0.964(8)	0.365 [c]
	O	16*h*	1 [b]	1.205 [c]

[a] A constraint was applied to fix the total molar ratio of Mn/Zn to 2.0, considering the ICP result for the material.
[b] The occupancy of the 16*h* site was fixed to 1 to decrease the number of fitting parameters.
[c] The Debye–Waller factor was determined assuming that Zn and Mn have the same value and was fixed at the final refinement.

Table S3 | Crystal structure of as-synthesized ZM19O. Crystal structure parameters determined by the Rietveld refinement for the as-synthesized ZM19O sample, where *g* and *B* indicate the occupancy and the Debye–Waller factor, respectively. A number in bracket indicates the standard deviation in the last digit.

ZM19O	Element	Site	*g*	*B*
As synthesized	Zn	4*a*	0.863(7)	0.365 [c]
	Zn	8*d*	0.040 [a]	0.365 [c]
	Mn	8*d*	0.896(8)	0.365 [c]
	O	16*h*	1 [b]	1.205 [c]

[a] A constraint was applied to fix the total molar ratio of Mn/Zn to 1.9, considering the ICP result for the material.
[b] The occupancy of the 16*h* site was fixed to 1 to decrease the number of fitting parameters.
[c] The Debye–Waller factor was fixed to the same values as those for the other samples.

Table S4 | Crystal structure of as-synthesized ZM18O. Crystal structure parameters determined by the Rietveld refinement for the as-synthesized ZM18O sample, where *g* and *B* indicate the occupancy and the Debye–Waller factor, respectively. A number in bracket indicates the standard deviation in the last digit.

ZM18O	Element	Site	*g*	*B*
As synthesized	Zn	4*a*	0.842(7)	0.365 [c]
	Zn	8*d*	0.055 [a]	0.365 [c]
	Mn	8*d*	0.856(7)	0.365 [c]
	O	16*h*	1 [b]	1.205 [c]

[a] A constraint was applied to fix the total molar ratio of Mn/Zn to 1.8, considering the ICP result for the material.
[b] The occupancy of the 16*h* site was fixed to 1 to decrease the number of fitting parameters.
[c] The Debye–Waller factor was fixed to the same values as those for the other samples.

Table S5 | Crystal structure of charged ZM18O. Crystal structure parameters determined by the Rietveld refinement for the charged ZM18O sample, where *g* and *B* indicate the occupancy and the Debye–Waller factor, respectively. A number in bracket indicates the standard deviation in the last digit.

ZM18O	Element	Site	*g*	*B*
Charged	Zn	4*a*	0.680(6)	0.365 [d]
	Zn	8*d*	0 [a]	0.365 [d]
	Mn	8*d*	0.856 [b]	0.365 [d]
	O	16*h*	1 [c]	1.205 [d]

[a] The value was slightly negative in the refinement, so it was fixed at 0.
[b] No Mn dissolution was assumed to make the calculation easier.
[c] The occupancy of the 16*h* site was fixed to 1 to decrease the number of fitting parameters.
[d] The Debye–Waller factor was fixed to the same values as those for the other samples.

Table S6 | Crystal structure of discharged ZM18O. Crystal structure parameters determined by the Rietveld refinement for the discharged ZM18O sample, where *g* and *B* indicate the occupancy and the Debye–Waller factor, respectively. A number in bracket indicates the standard deviation in the last digit.

ZM18O	Element	Site	*g*	*B*
Discharged	Zn	4*a*	0.74(11)	0.365 [c]
	Zn	8*d*	0.10(14)	0.365 [c]
	Mn	8*d*	0.856 [a]	0.365 [c]
	O	16*h*	1 [b]	1.205 [c]

[a] No Mn dissolution was assumed to make the calculation easier.
[b] The occupancy of the 16*h* site was fixed to 1 to decrease the number of fitting parameters.
[c] The Debye–Waller factor was fixed to the same values as those for the other samples.

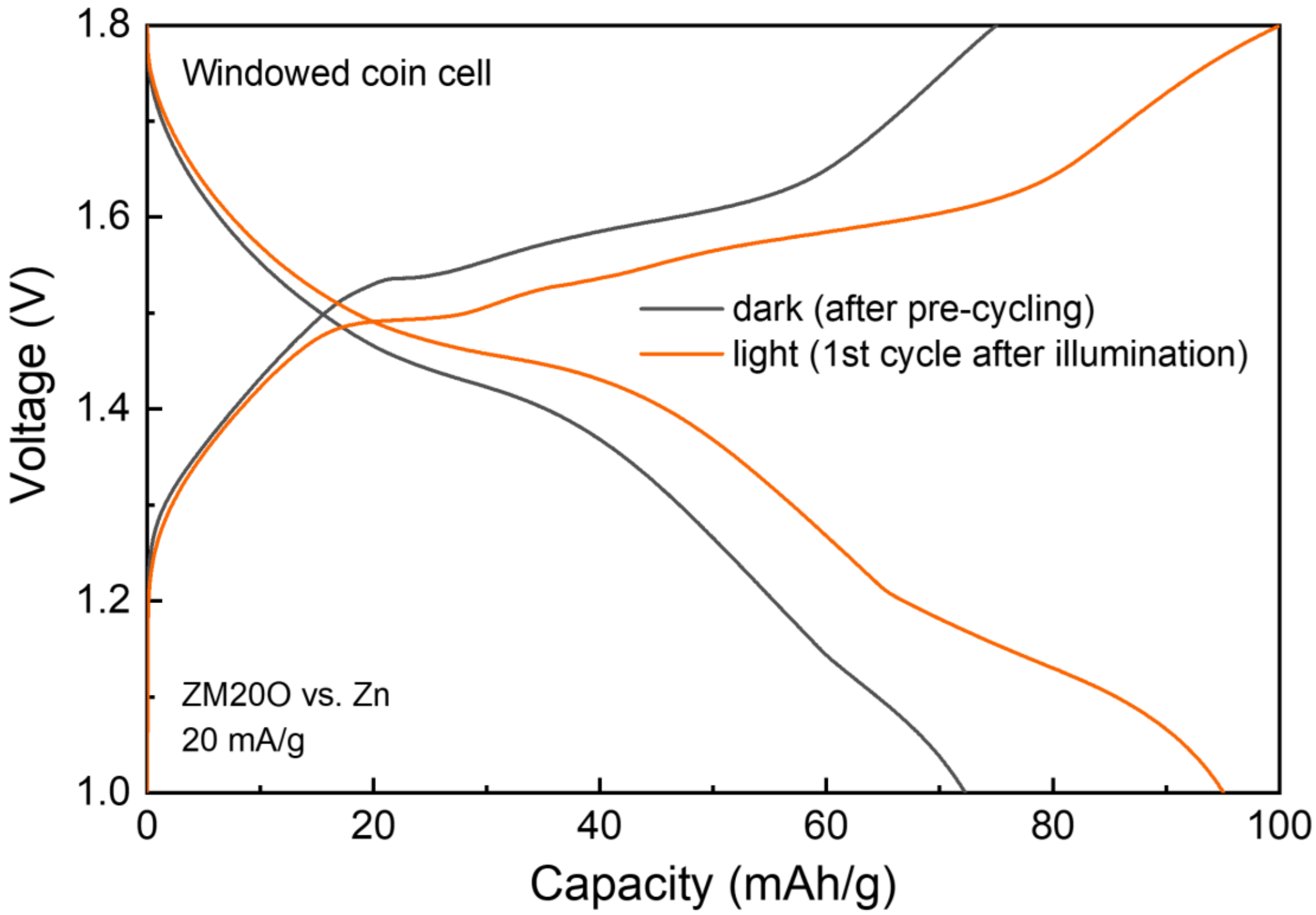

Fig. S5 | Photo-enhanced operation of the ZMO cathode. After the dark pre-cycling process, the specific capacity was 70–80 mAh/g; however, the capacity increased by approximately 20 mAh/g under the illumination of the windowed coin cell. Note that an LED was used here exceptionally for consistency with the setup in previous works [5]. The obtained behavior demonstrates the operation of photo-enhanced battery systems, where light-induced heating can kinetically enhance the capacity, as discussed in previous literature [6].

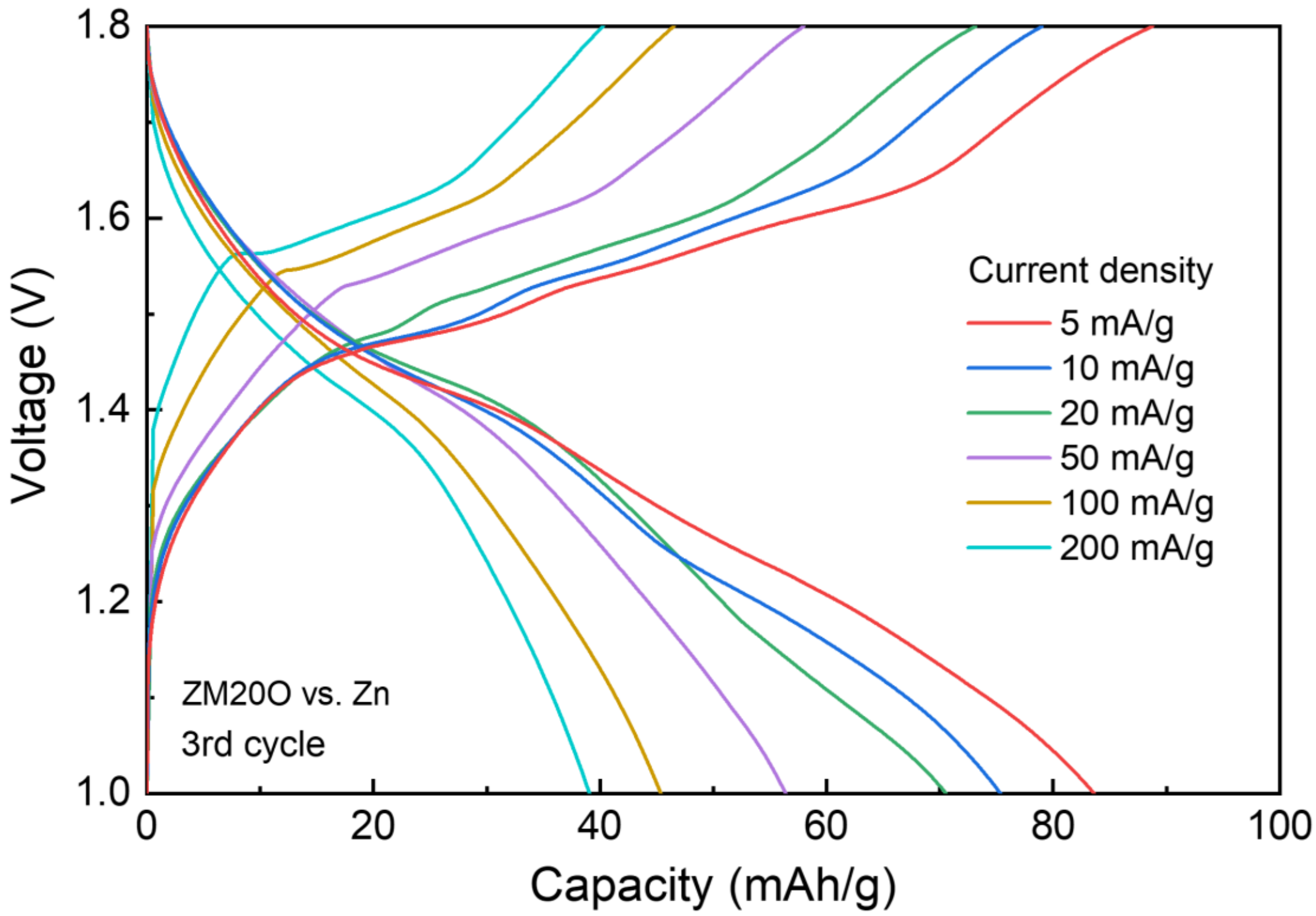

Fig. S6 | Rate capability of the ZMO cathode. The specific capacity decreased from approximately 80 to 40 mAh/g as the current density increased from 5 to 200 mA/g. This reduction indicates the capacity is kinetically limited, attributed to the sluggish diffusion of divalent cations.

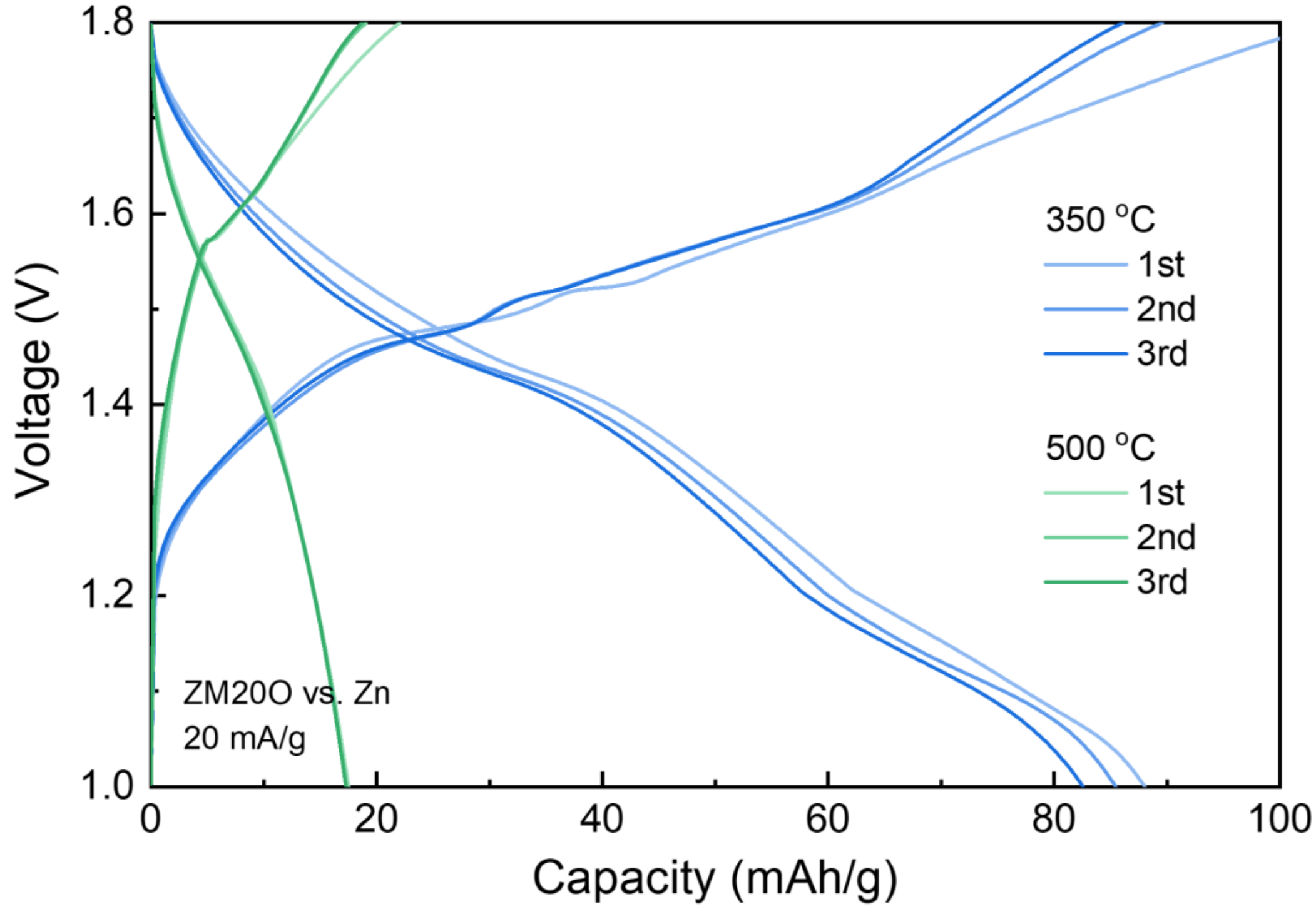

Fig. S7 | Calcination-temperature dependence of the ZMO cathode. The specific capacity decreased from approximately 80 to 20 mAh/g, as the calcination temperature increased from 350 to 500 °C. Given that the particle size increases at higher temperatures, the reduction in capacity is consistent with the kinetic limitation arising from solid-state diffusion.

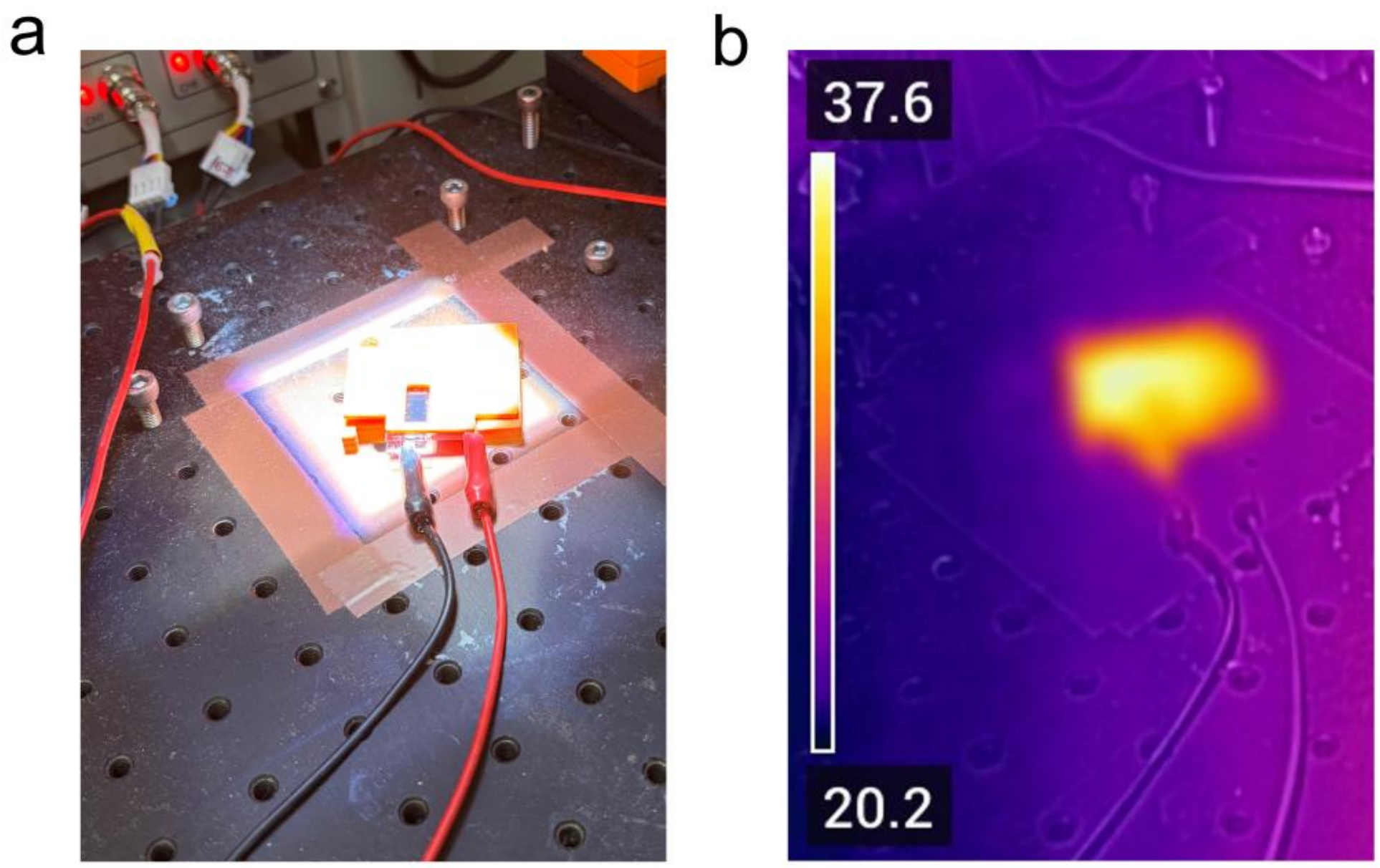

Fig. S8 | Temperature increase under 1-sun illumination. (a) The optical cell was illuminated using a solar simulator during CV measurements of the ZMO cathode. (b) The corresponding thermal camera image clearly shows the temperature distribution under 1-sun illumination. The cell temperature increased to approximately 36 ℃ in 10 min, demonstrating the thermal effects of the illumination.

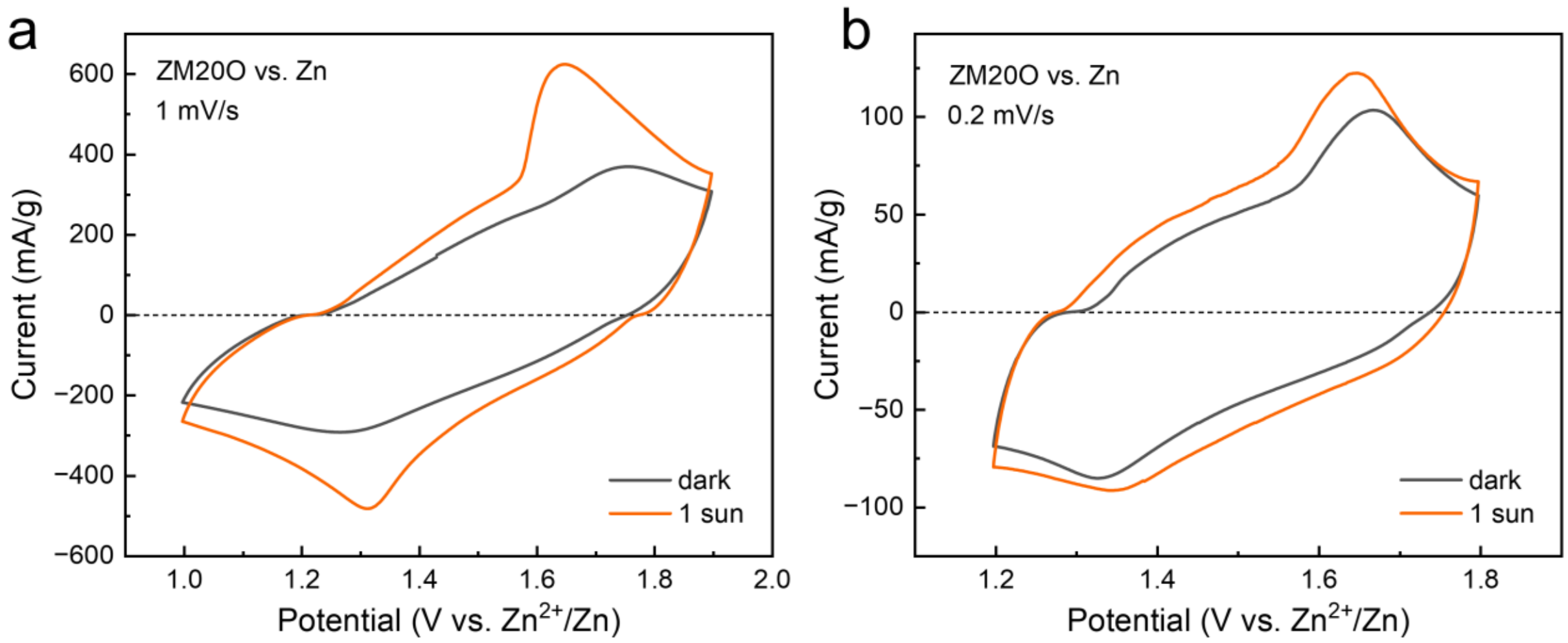

Fig. S9 | Photo-enhanced behavior of the ZMO cathode. CV curves in the dark and under 1-sun illumination at scan rates of (a) 1 mV/s and (b) 0.2 mV/s. For this measurement, an electrode composed of ZM20O, Super-P, CMC, and SBR in a weight ratio of 80:12:4:4 was coated onto an FTO substrate to construct an optical cell. The enhancement in redox current indicates the kinetic increase in battery reactions.

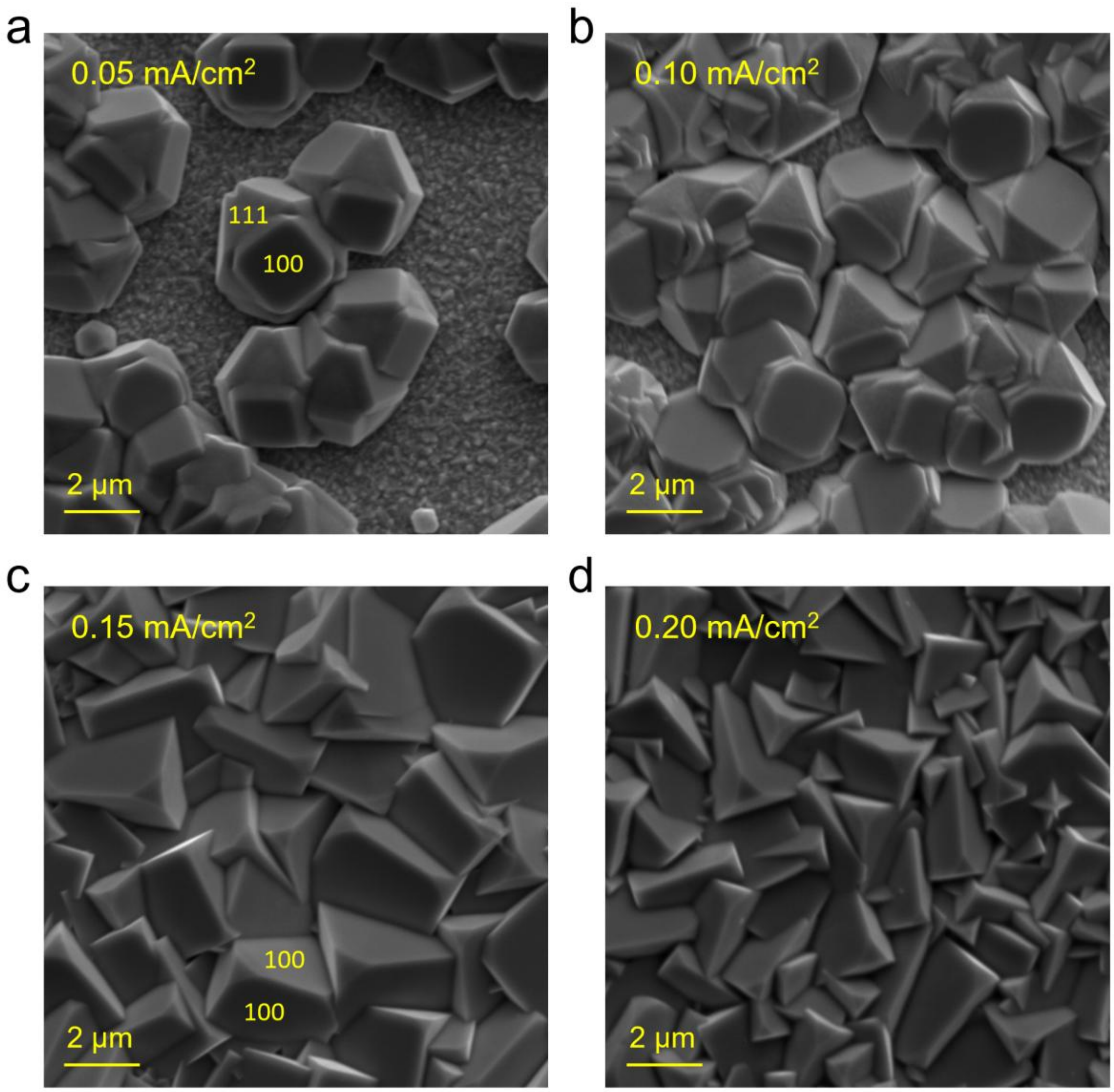

Fig. S10 | SEM images of electrodeposited Cu_2O. The morphology of the electrodeposited Cu_2O films prepared at (a) 0.05, (b) 0.10, (c) 0.15, and (d) 0.20 mA/cm^2 (deposition time fixed at 180 min) was characterized by SEM using a secondary electron detector. According to previous literature [7], the triangular surfaces correspond to (111) planes, while the side facets are (100) planes; the latter become dominant at 0.15 mA/cm^2.

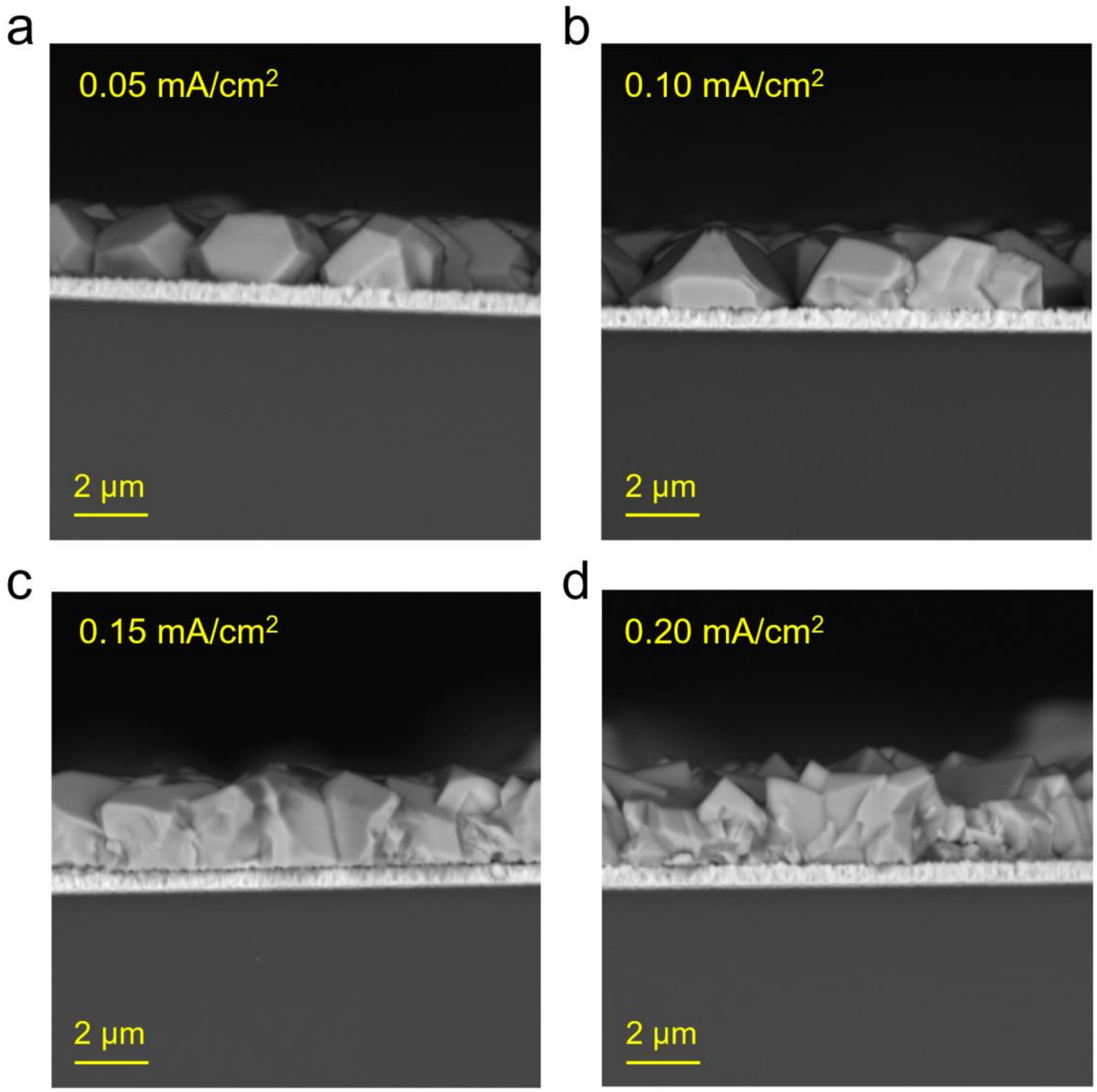

Fig. S11 | Cross-sectional SEM images of electrodeposited Cu_2O. Cross-sections of Cu_2O/FTO substrates, prepared by electrodeposition at (a) 0.05, (b) 0.10, (c) 0.15, and (d) 0.20 mA/cm^2 (deposition time fixed at 180 min), were characterized by SEM. While the thickness remained approximately 2 μm across all samples, the morphology changed drastically: relatively isolated, large crystals were observed at lower current densities (0.05 and 0.10 mA/cm^2), whereas the films became more compact and continuous at 0.15 mA/cm^2; finally, the surface roughness increased again at 0.20 mA/cm^2.

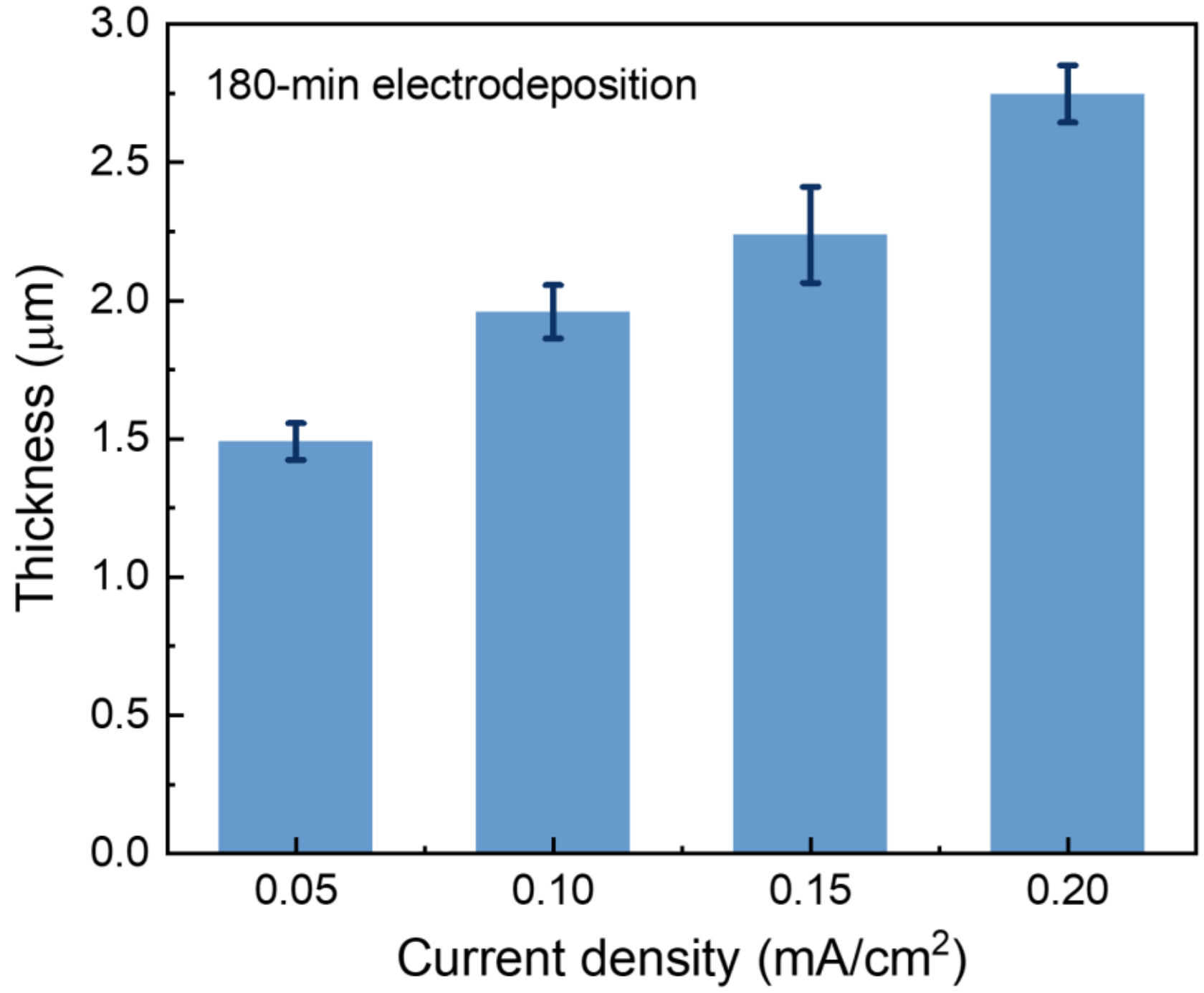

Fig. S12 | Profilometry of electrodeposited Cu_2O. Thickness of the electrodeposited Cu_2O on FTO at different current densities (0.05, 0.10, 0.15, and 0.20 mA/cm^2) was determined by profilometry. Since the electrodeposition time was fixed at 180 min, the average thickness increased with increasing current density. The representative thickness was approximately 2 μm, which is consistent with the cross-sectional SEM observations.

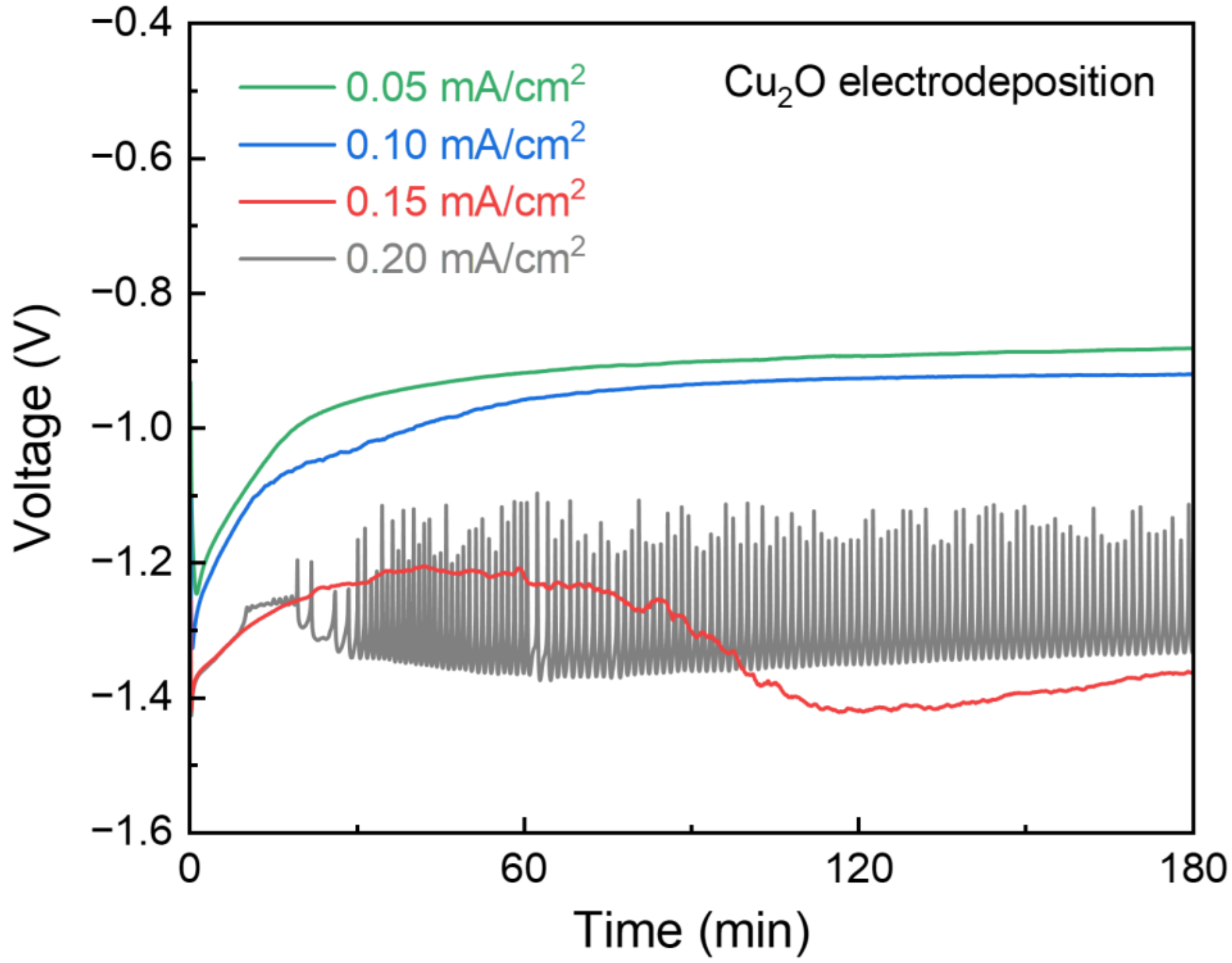

Fig. S13 | Voltage profiles during Cu_2O electrodeposition. The voltage between the FTO working electrode and the Ti counter electrode was monitored during the galvanostatic electrodeposition of Cu_2O at various current densities (0.05, 0.10, 0.15, and 0.20 mA/cm^2). Voltage fluctuation was observed at 0.20 mA/cm^2, suggesting the occurrence of side reactions that lead to the incorporation of metallic Cu, as indicated by the XRD results.

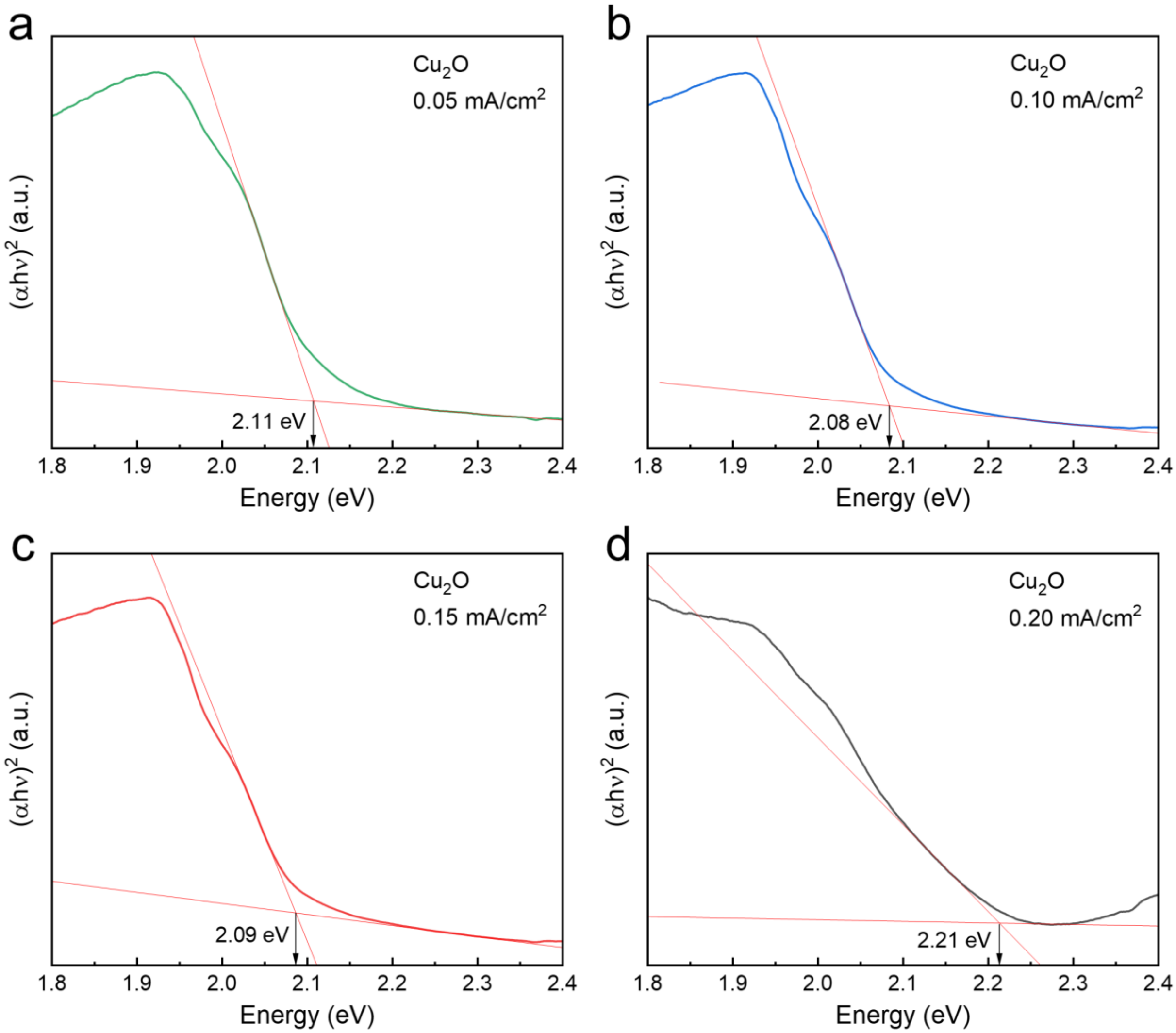

Fig. S14 | Bandgap of electrodeposited Cu_2O. The optical bandgaps of Cu_2O films prepared at various current densities, (a) 0.05, (b) 0.10, (c) 0.15, and (d) 0.20 mA/cm^2, were estimated using Tauc plots [8]. A bandgap of approximately 2.1 eV, consistent with previous literature [9], was obtained for most samples. An exception was observed for the 0.20 mA/cm^2 sample, which can be attributed to the incorporation of metallic Cu as indicated in the XRD results.

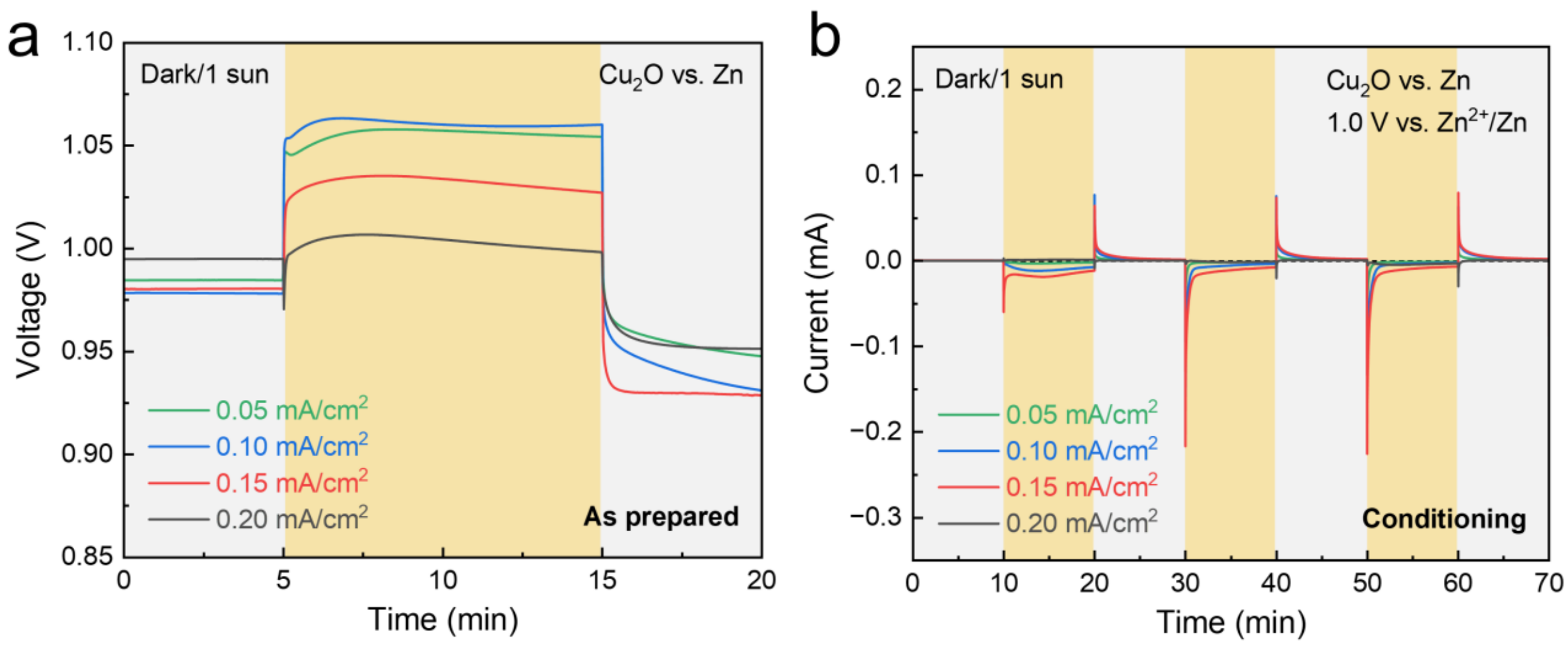

Fig. S15 | Photoresponse of electrodeposited Cu_2O films. The electrochemical behavior of the cells coupling Cu_2O and Zn was evaluated under chopped light illumination using (a) OCV and (b) CA at a bias potential of 1.0 V vs. Zn^{2+}/Zn. In contrast to the distinct photoresponse for most of the electrodes, the sample prepared at 0.20 mA/cm^2 exhibited negligible photovoltage and photocurrent, which is consistent with the incorporation of Cu confirmed by XRD. Additionally, a conditioning process was observed during chronoamperometric cycling, and the photovoltage eventually increased to above 0.1 V, as shown below and in the manuscript.

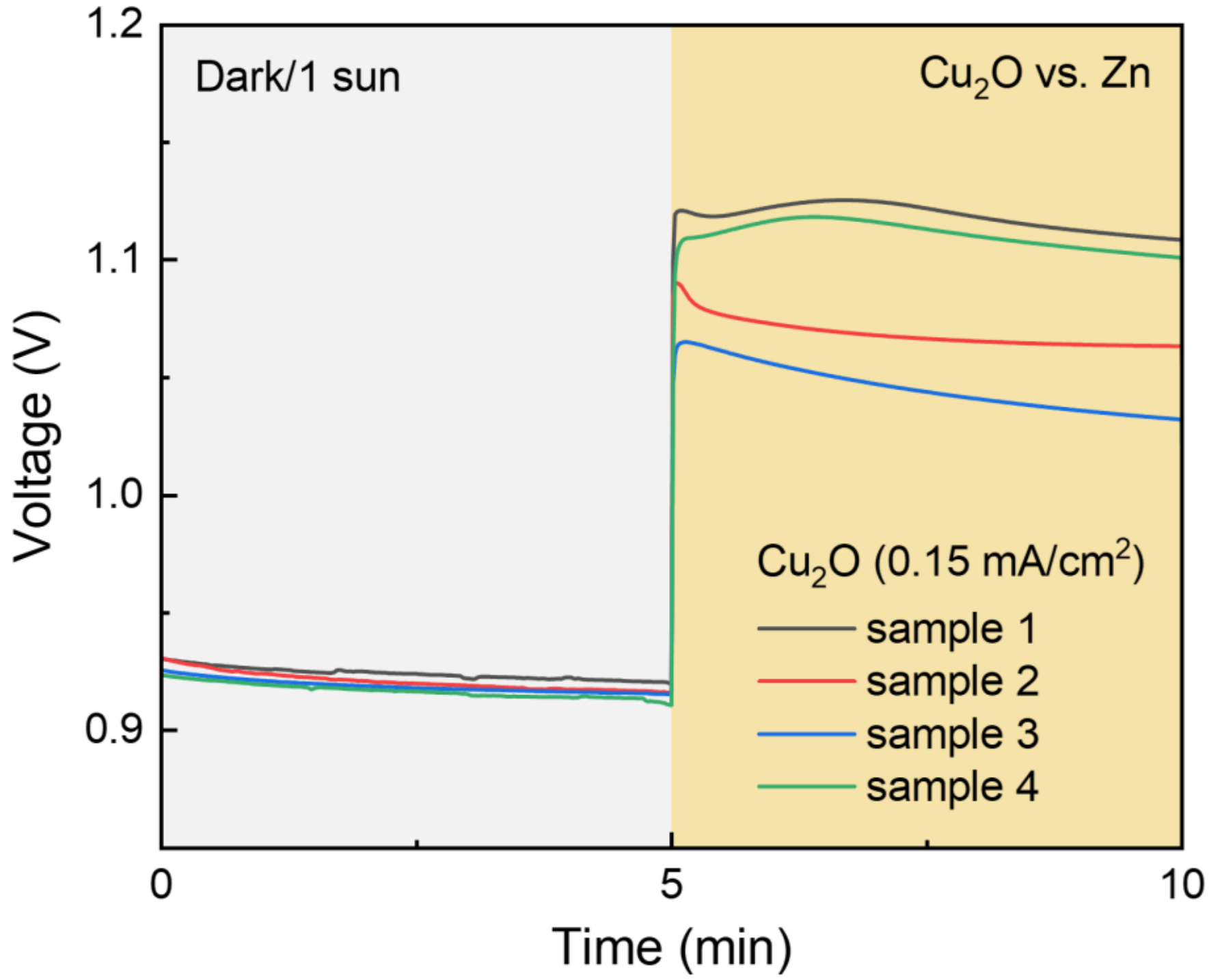

Fig. S16 | Repetitive OCV tests of Cu_2O photoresponse. Cu_2O films were electrodeposited at 0.15 mA/cm^2, and their OCV profiles were measured in the dark and under 1-sun illumination after the conditioning process. Every cell showed over 0.1 V photovoltage, but the broad oxidation peak after several minutes of illumination was non-reproducible, presumably stemming from a thermally activated side reaction.

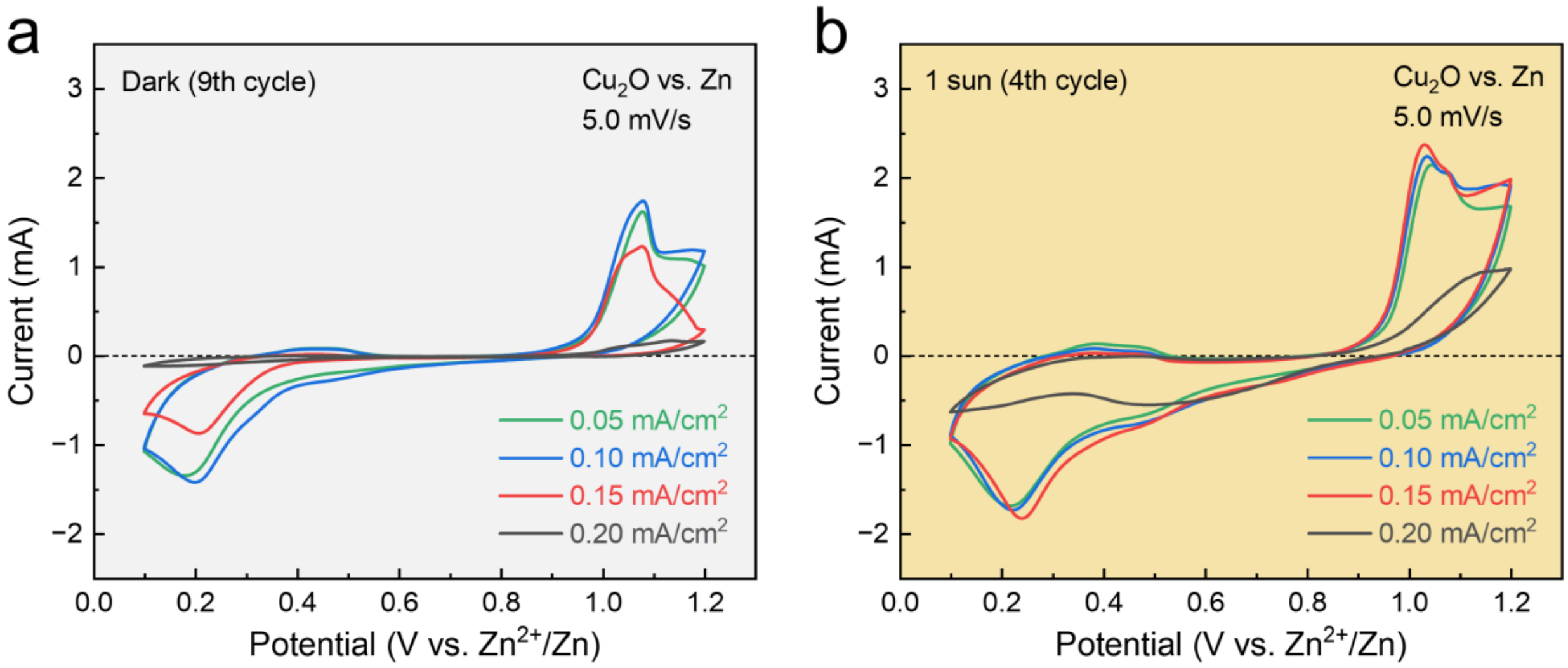

Fig. S17 | Dark and light CV curves of Cu_2O. CV was performed on Cu_2O films prepared at various current densities (0.05, 0.10, 0.15, and 0.20 mA/cm^2) at a scan rate of 5.0 mV/s under (a) dark and (b) light conditions. The electrochemical behavior was similar across the samples, except for the one prepared at 0.20 mA/cm^2, which is consistent with other characterizations. The enhanced capacity under 1-sun illumination can be attributed to photothermal effects as well as increased conductivity.

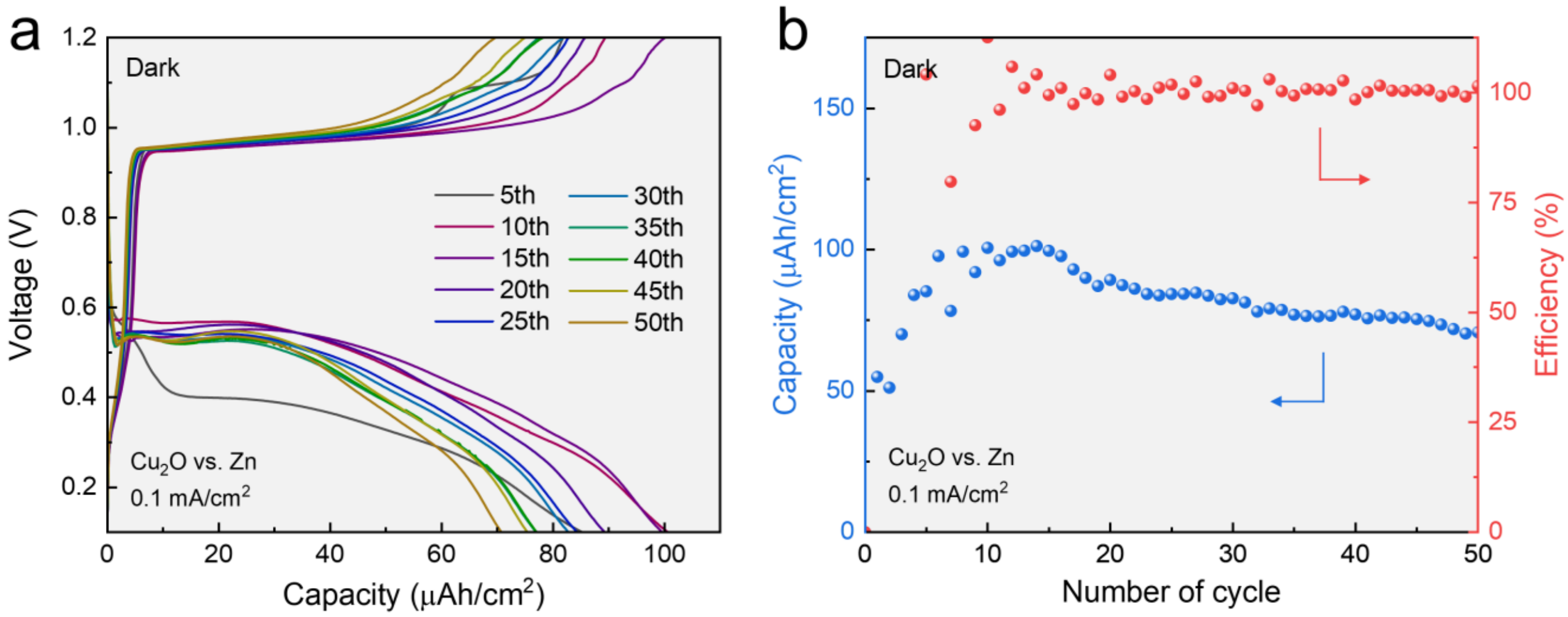

Fig. S18 | Dark galvanostatic charge/discharge of Cu_2O vs. Zn. The cyclic stability of the Cu_2O redox reaction in the Zn-ion battery system under dark conditions was evaluated by (a) galvanostatic charge/discharge profiles and (b) the corresponding capacity and Coulombic efficiency plots. After the initial activation process over the first 10 cycles, the Coulombic efficiency stabilized at nearly 100%. A gradual capacity fading was observed over 50 cycles, which can be attributed to the inherent nature of conversion-type reactions.

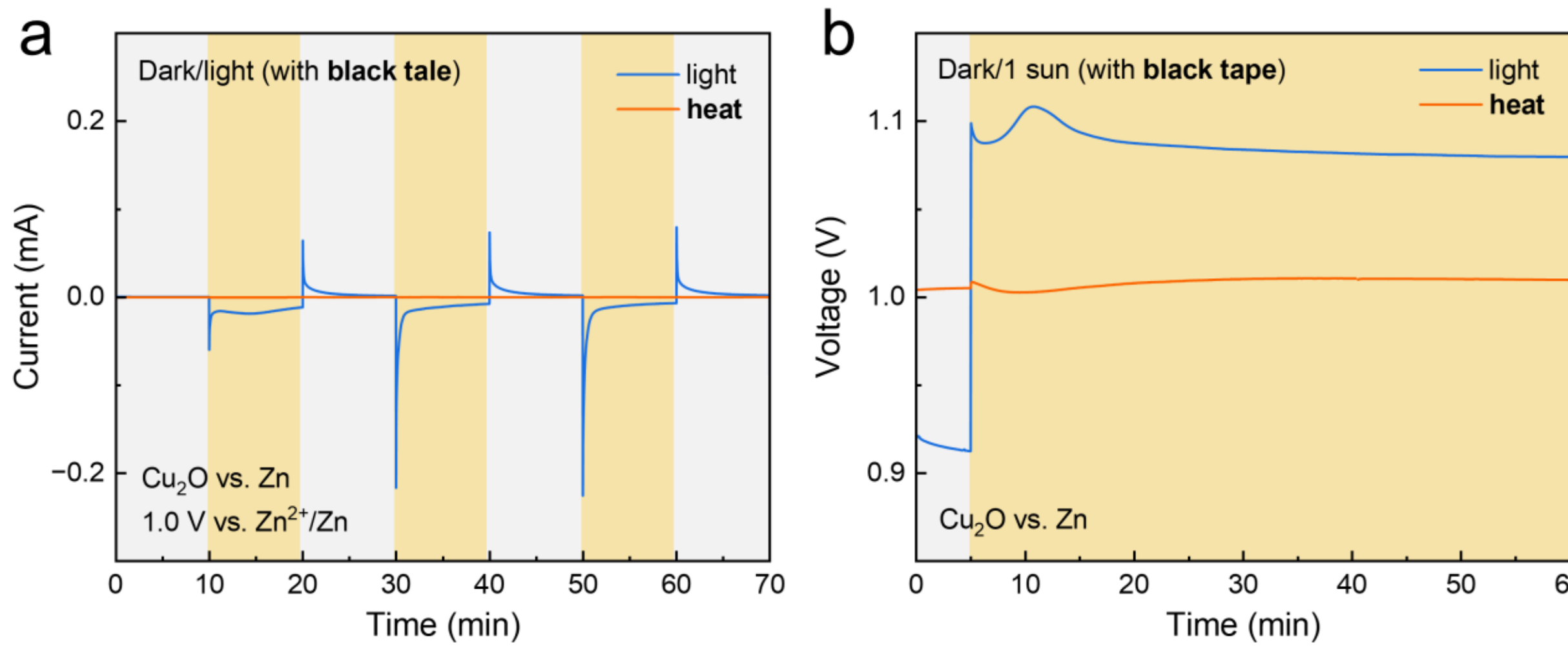

Fig. S19 | Thermal effects on the photovoltage of Cu_2O. The difference in photovoltaic behavior was compared between the cells with a transparent optical window (light) and those covered with black tape (heat) using (a) CA and (b) OCV measurements. The negligible response to illumination in the black-tape covered cells indicates that the contribution of thermal effects to electrode potential is of little significance.

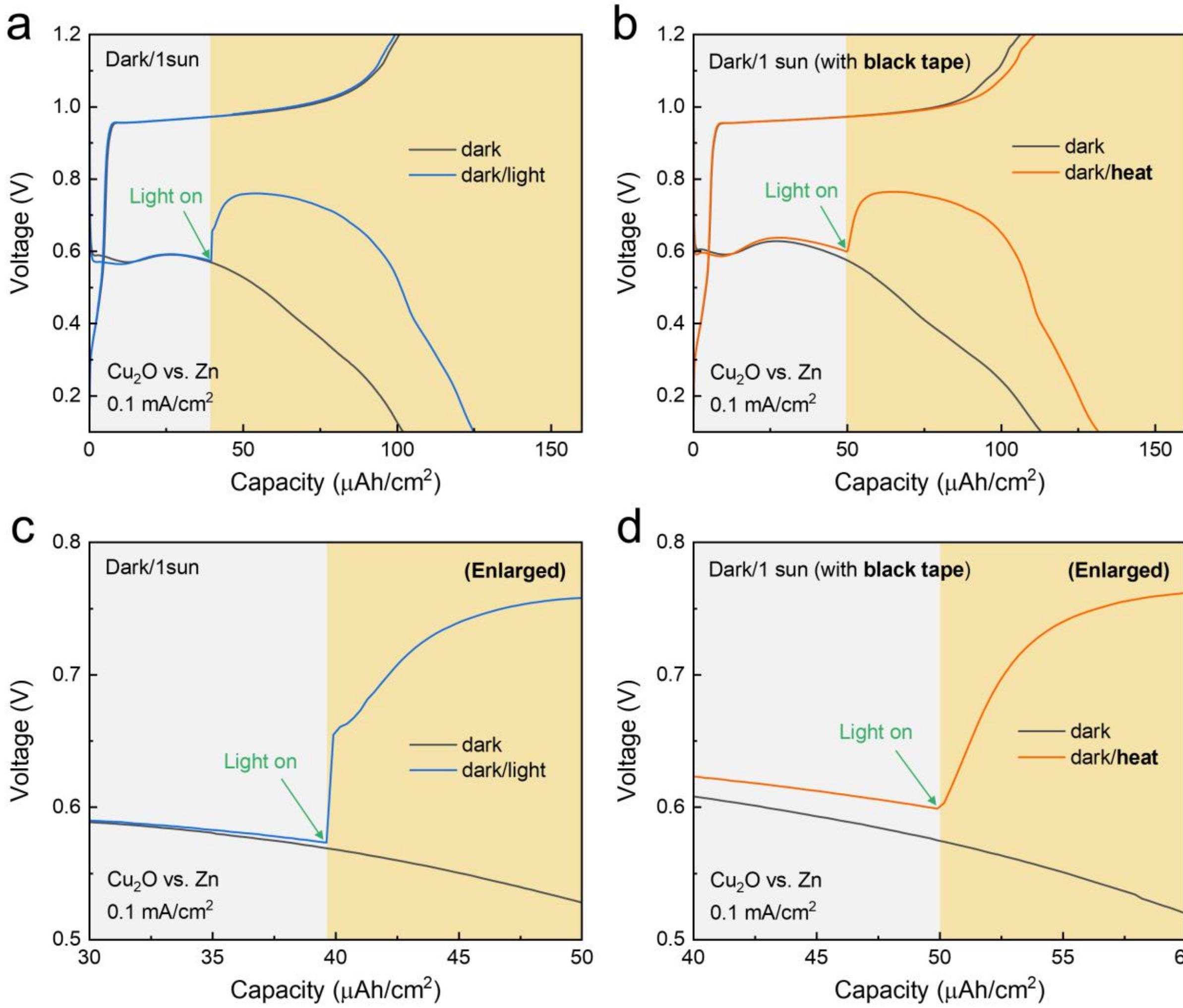

Fig. S20 | Thermal effects in the galvanostatic Cu_2O photoelectrode operation. The electrochemical behavior in the photo-enhanced battery protocol was investigated by applying 1-sun illumination during galvanostatic discharge. The optical window was either (a) kept transparent (light) or (b) covered with black tape (heat) to eliminate the optical effects. Panels (c) and (d) show enlarged views of (a) and (b), respectively. The similarity in discharge capacity suggests that photothermal effects were dominant in determining the photo-enhanced capacity [6], while the sharp voltage response in the absence of black tape upon illumination indicates a concurrent photoelectrochemical response.

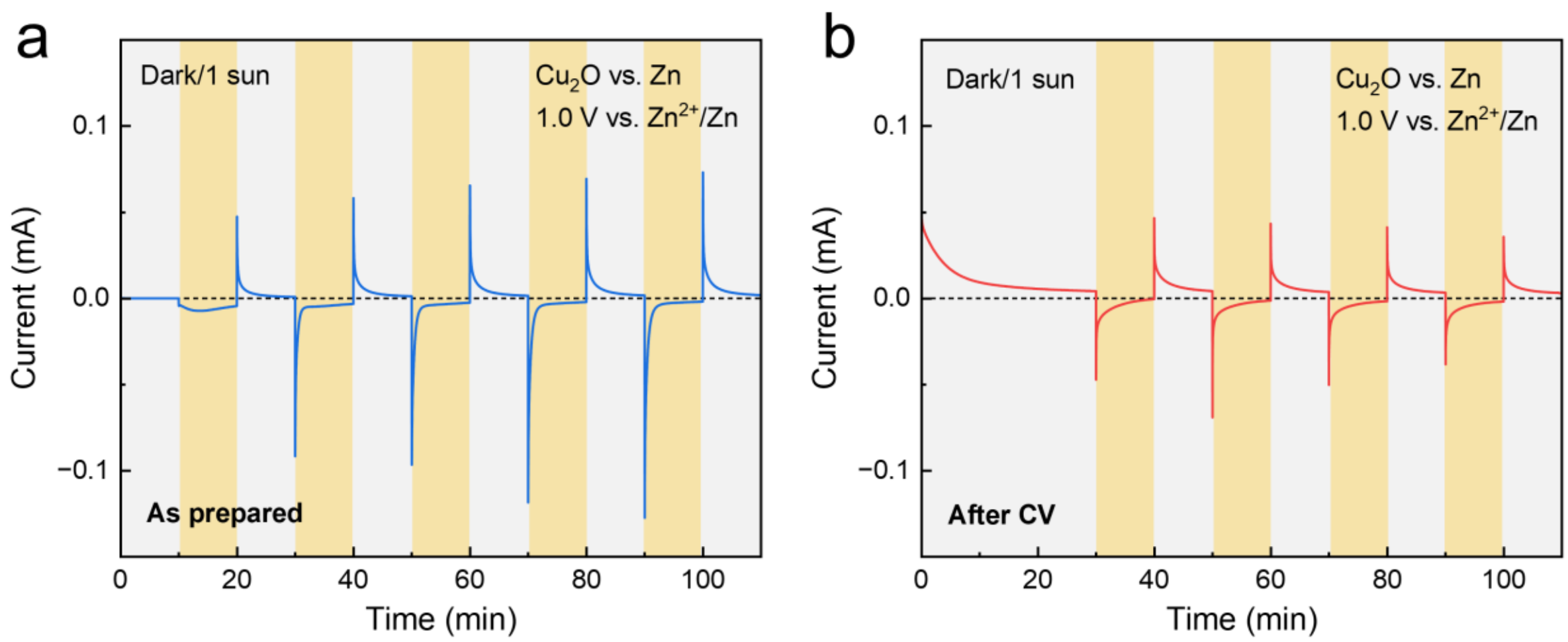

Fig. S21 | Photoresponse of Cu_2O before and after CV cycling. Chronoamperometry was performed on the Cu_2O film (0.15 mA/cm^2) in its (a) as-prepared state and (b) after CV cycling. Even after activating the battery reactions through CV cycles, the photoresponse under chopped light illumination remained essentially identical to that of the as-prepared Cu_2O film. This suggests that the photoactivity is well-preserved during bifunctional battery operation, owing to the robust film architecture

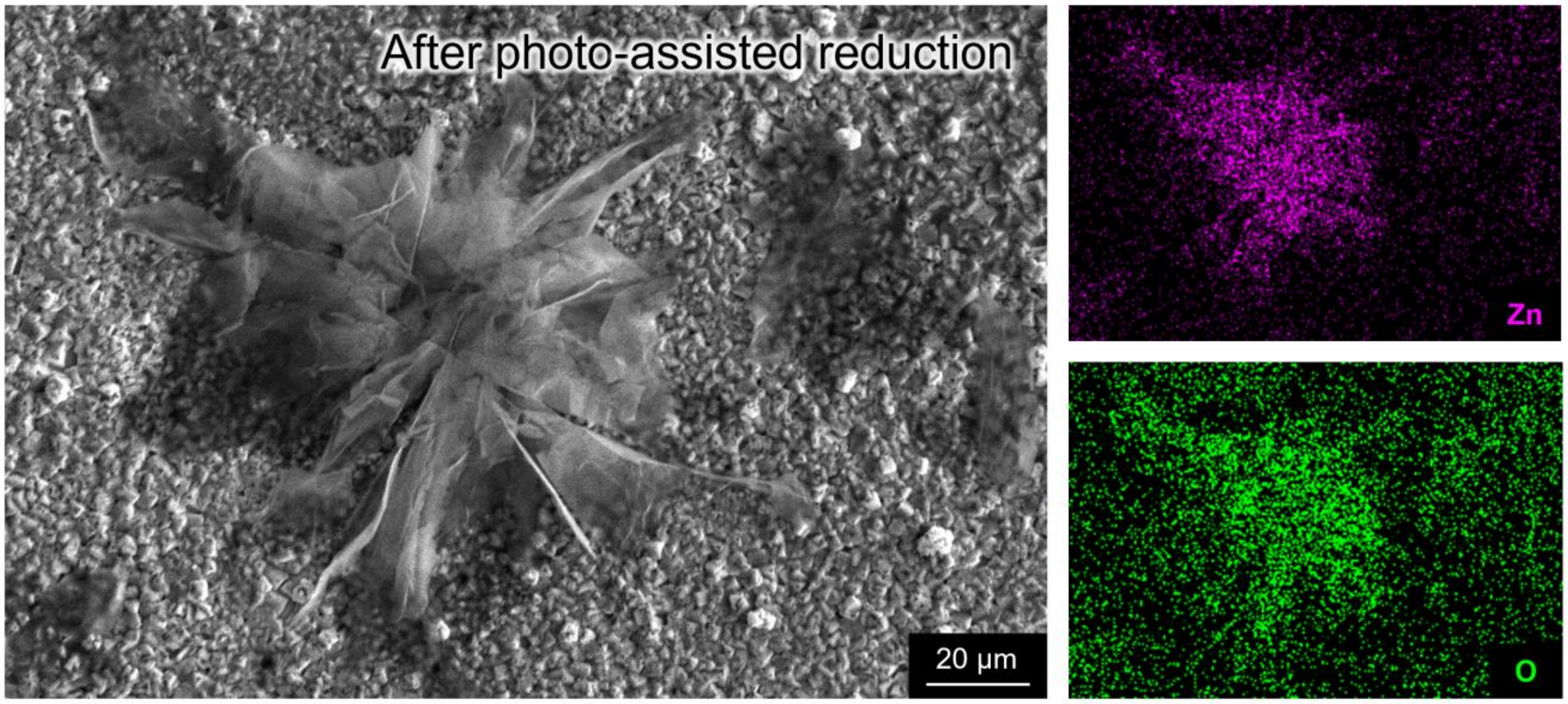

Fig. S22 | EDS mapping after the photo-assisted reduction of Cu_2O. Elemental mapping by EDS demonstrated that zinc oxide is deposited on the surface of the Cu_2O photoelectrode after the photo-assisted reduction process. Such deposits were absent in the control sample, which was merely soaked in the electrolyte. The non-uniform deposition suggests the existence of hot spots for light-driven reactions, likely arising from the rough morphology of the Cu_2O film. The less crystalline nature of these deposits is consistent with the absence of ZnO diffraction peaks in the XRD profiles.

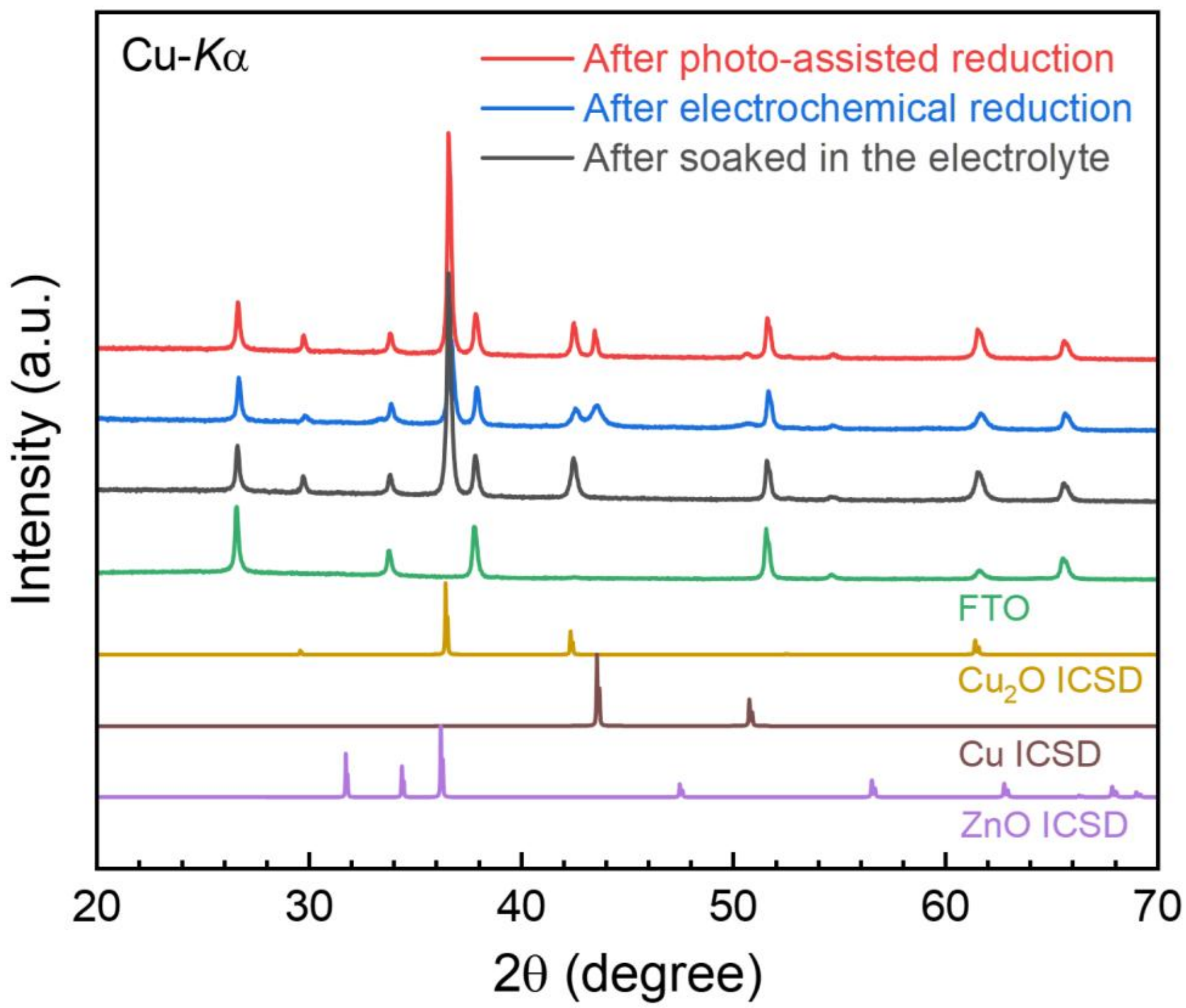

Fig. S23 | XRD analysis after the photo-assisted and electrochemical reduction on Cu_2O. Structural changes among the samples subjected to chronoamperometric photo-assisted reduction, galvanostatic electrochemical reaction, and a control sample that merely soaked in the electrolyte, were investigated by XRD. The profiles of the photo-assisted and electrochemically reduced samples were identical, both exhibiting diffraction peaks from metallic Cu. The control sample remained virtually unchanged from its pristine state, supporting its stability in the electrolyte.

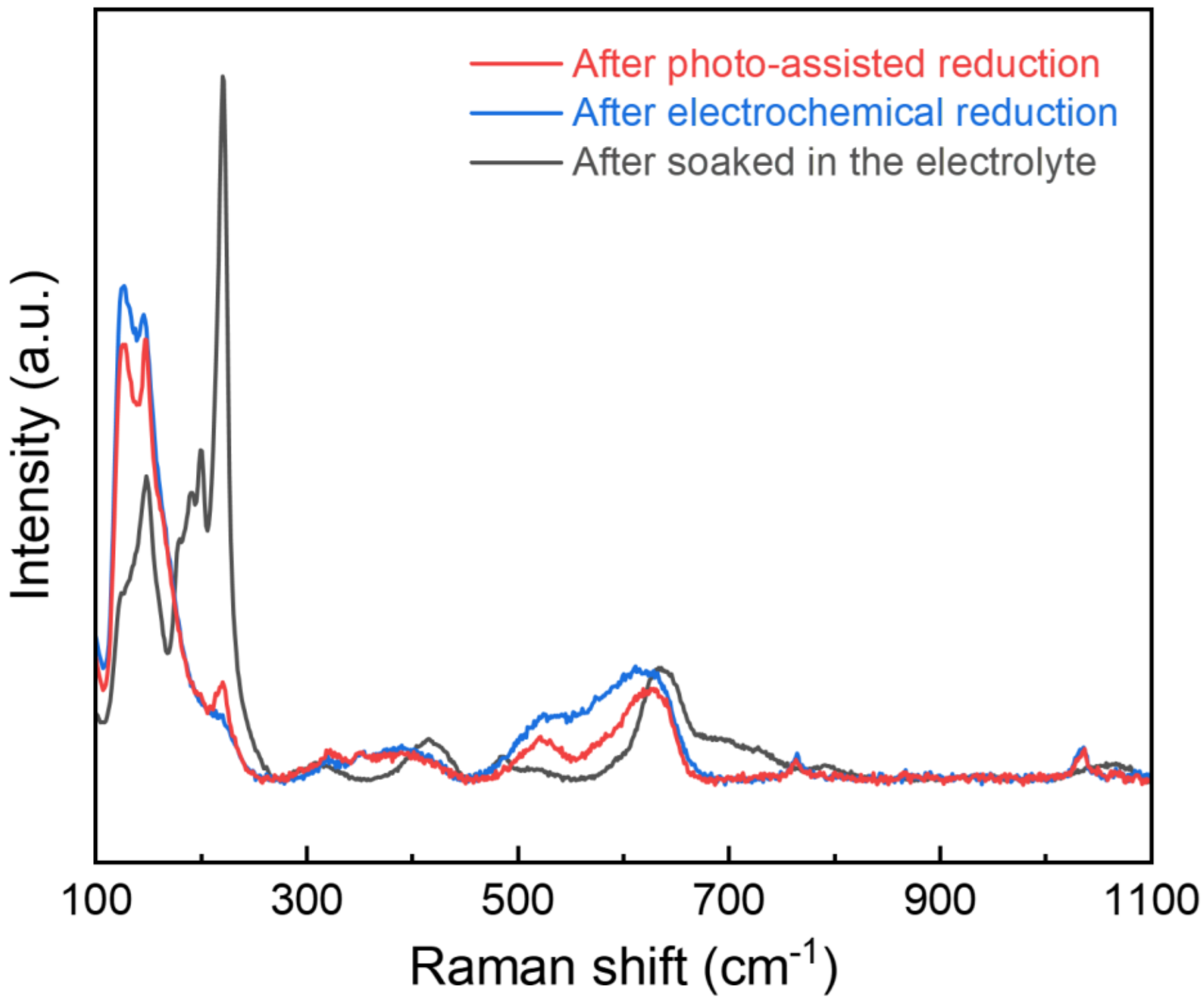

Fig. S24 | Raman spectroscopy after the photo-assisted and electrochemical reduction on Cu_2O. The same samples used for XRD analysis were further characterized by Raman spectroscopy. Consistent with the XRD results, the Raman spectra of the photo-assisted and electrochemically reduced samples were almost identical, reinforcing the conclusion that similar reactions occurred on these Cu_2O electrodes. The profile of the control sample was consistent with previous literature on Cu_2O [10].

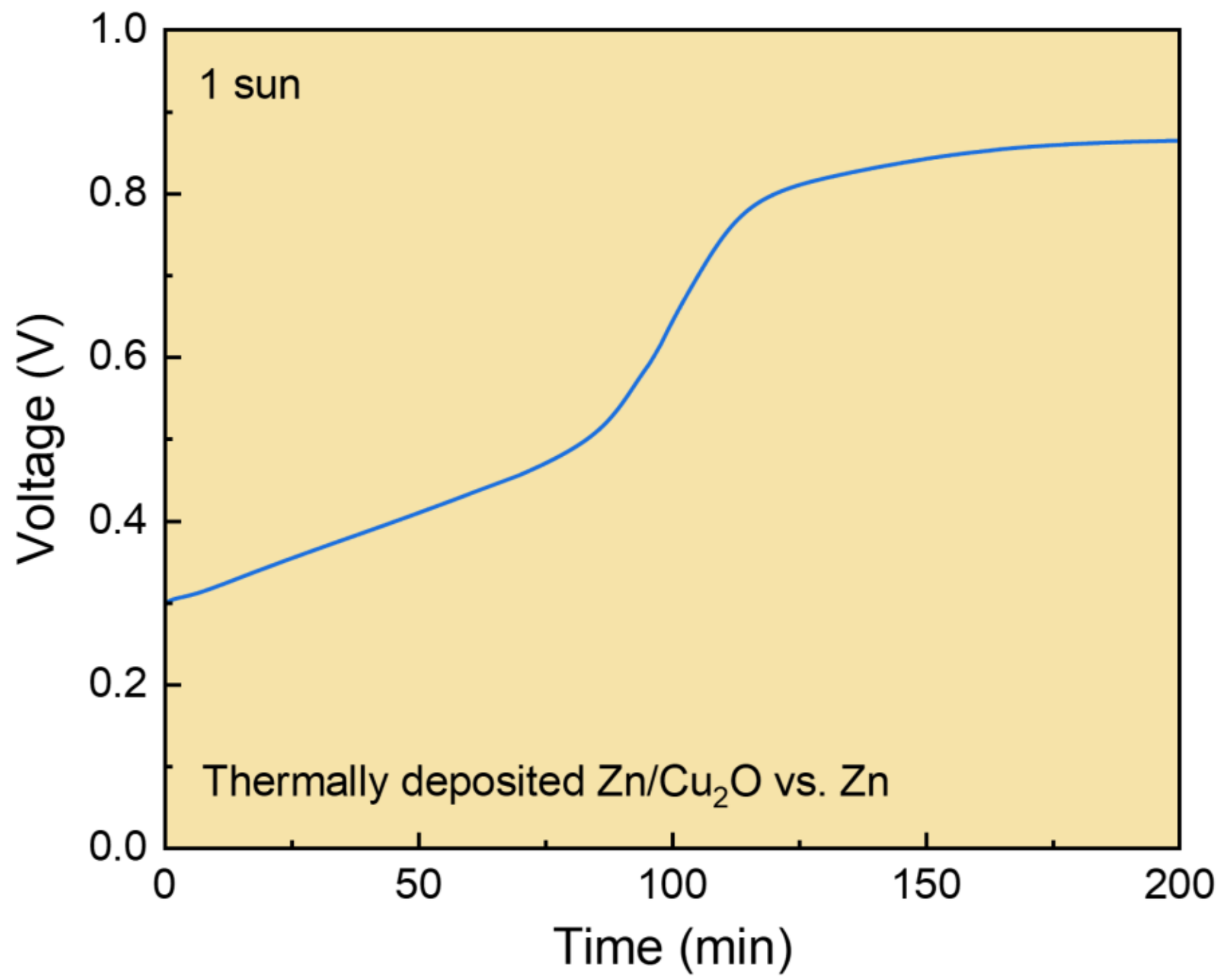

Fig. S25 | Light OCV of the thermally deposited Zn/Cu_2O electrode. Metallic Zn was thermally deposited on the Cu_2O film electrode to construct a cell with Zn-metal anode. The OCV gradually increased under 1-sun illumination, suggesting a spontaneous reaction between Cu_2O and Zn. The changes in color and morphology observed after the light OCV also supported the conversion reaction.

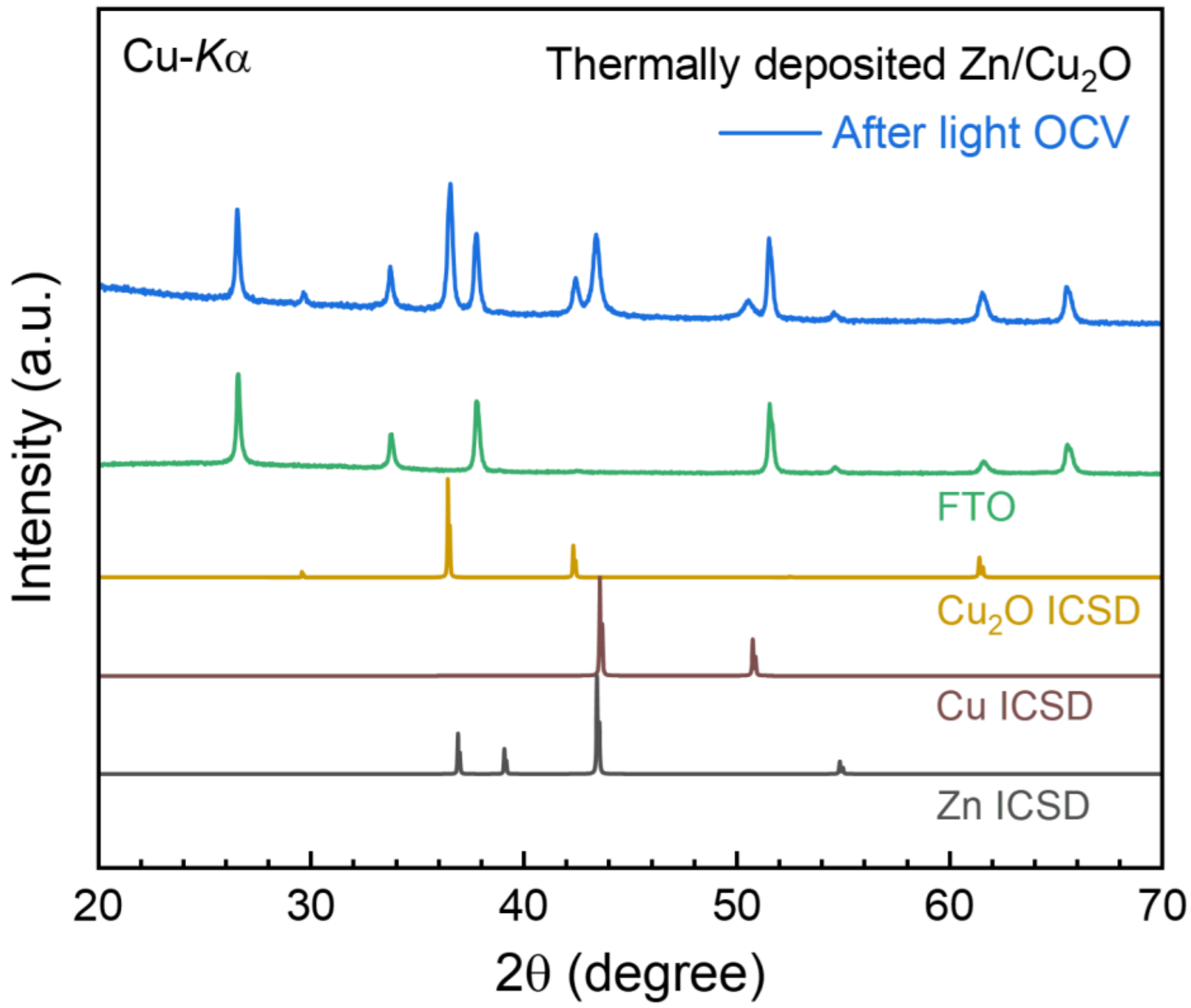

Fig. S26 | XRD analysis of the thermally deposited Zn/Cu_2O electrode after the light OCV measurement. Following the aforementioned light OCV measurement, XRD analysis was conducted to investigate the structural changes. The formation of metallic Cu was confirmed, which is consistent with the results of electrochemically prepared samples discussed previously.

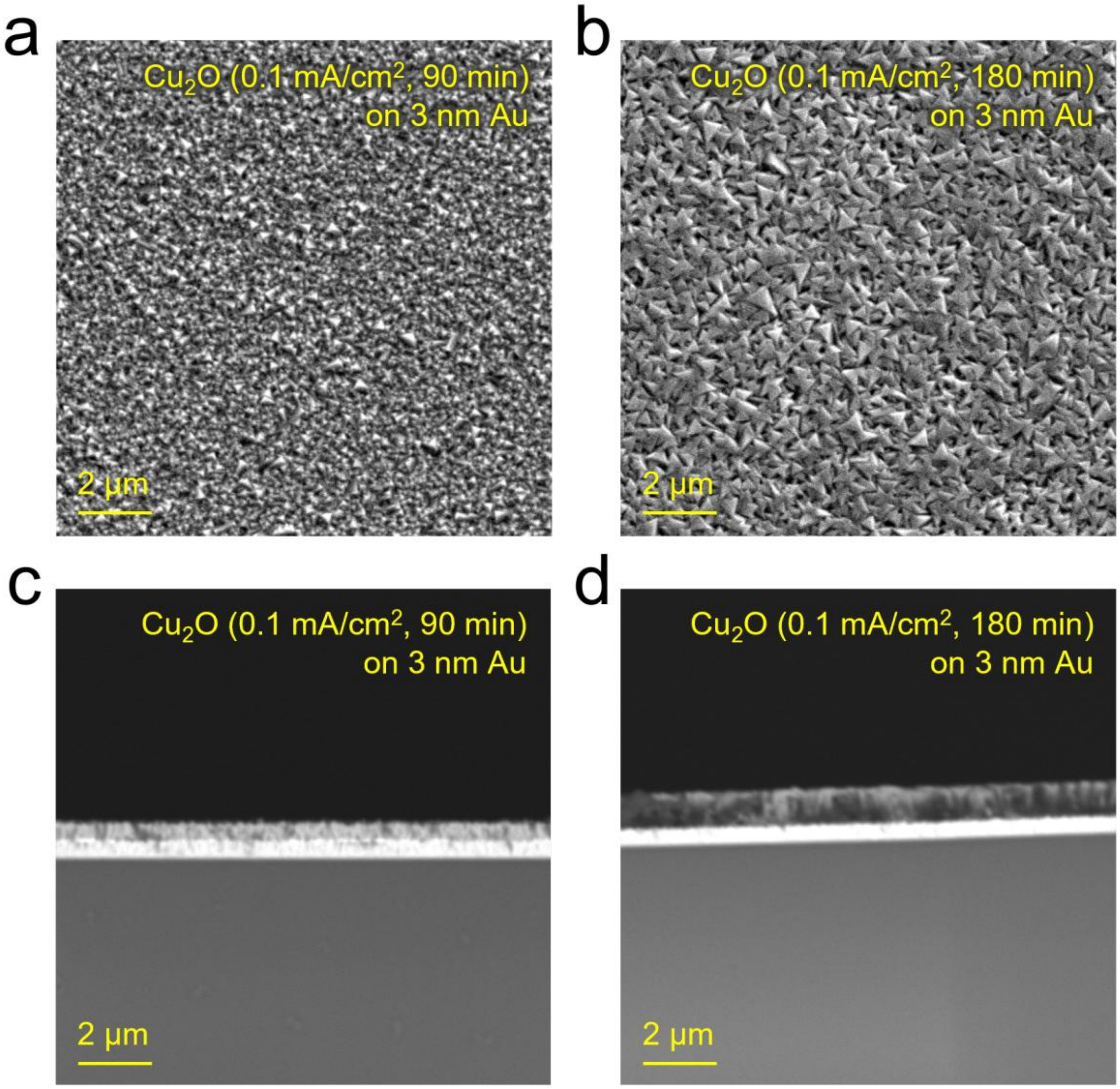

Fig. S27 | SEM images of the Cu_2O films electrodeposited on thin Au. The morphology and thickness of Cu_2O films electrodeposited on thin Au/FTO substrates were characterized by SEM. The current density was set at 0.10 mA/cm^2, following the previous literature [2]. (a,b) The crystal size significantly decreased upon introducing the Au layer [9], and the triangular facets indicate a [111] orientation [11], which was further confirmed by XRD. (c,d) The thickness was approximately 0.5 and 1.0 μm for the films electrodeposited for 90 and 180 min, respectively. Note that the brightest layer with a thickness of ~0.5 μm in the cross-sectional figures is FTO; the Au layer is invisible at this resolution.

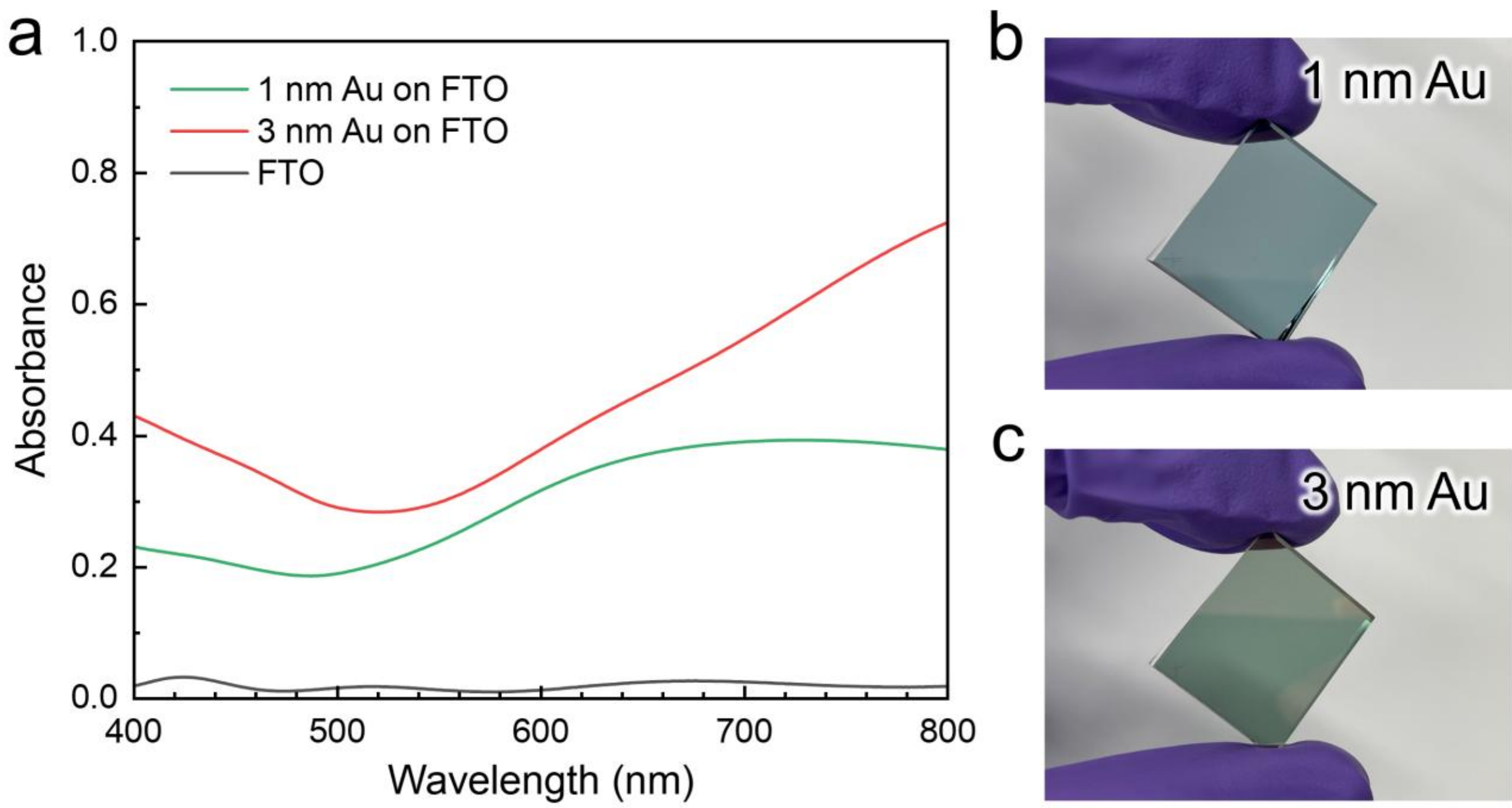

Fig. S28 | Transparency of the thermally deposited thin Au films. The optical properties of thermally deposited Au films were characterized by (a) UV–vis spectroscopy, where the nominal film thickness was controlled at (b) 1 nm and (c) 3 nm using a thermal evaporator. The absorbance of the 3-nm sample was approximately 40% in the 400–600 nm region, which is consistent with a previous report [9]. Despite the higher transparency of the 1-nm Au film, the 3-nm film was selected for Cu_2O electrodeposition due to its superior physical stability.

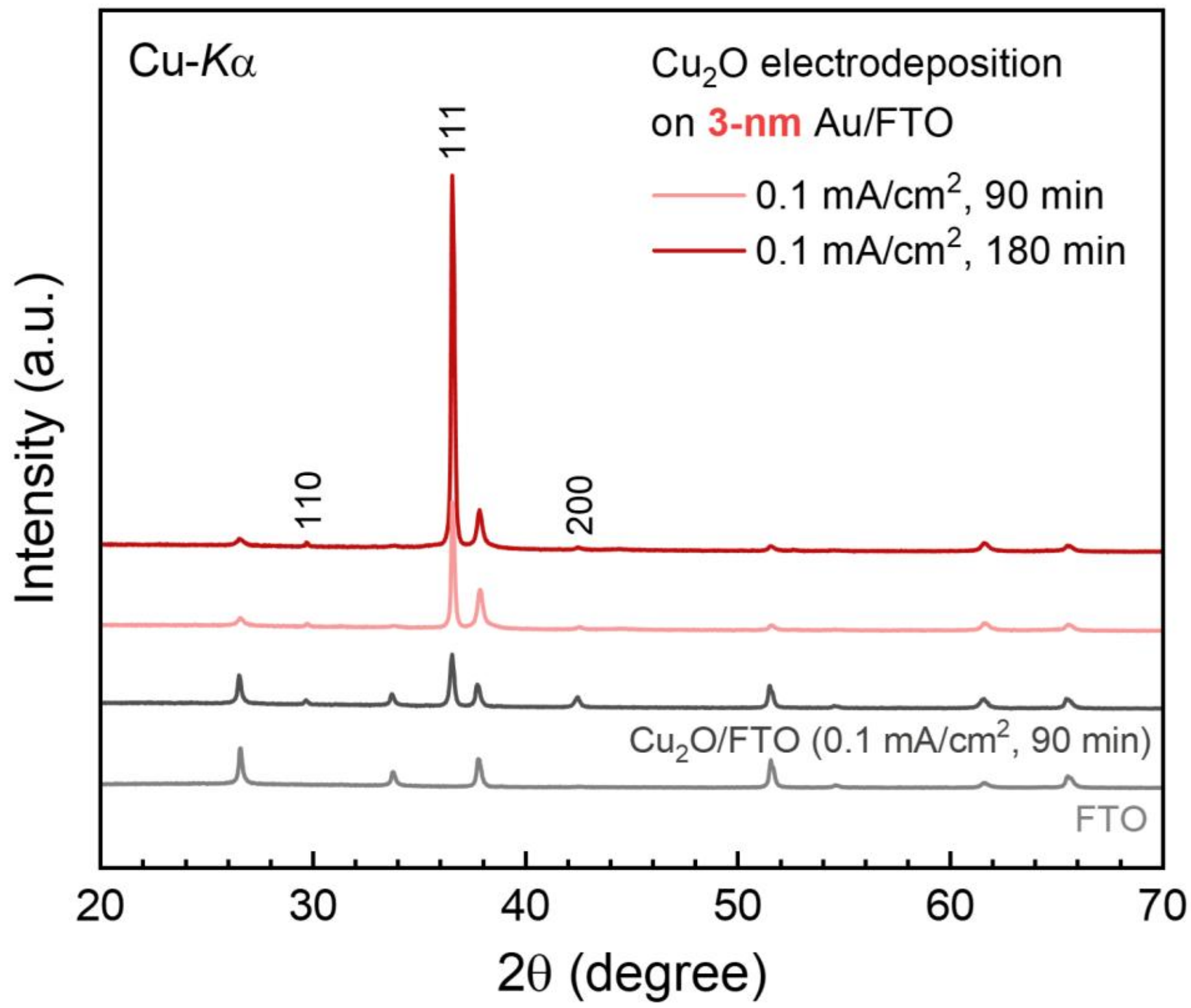

Fig. S29 | XRD profiles of Cu_2O films electrodeposited on thin Au. The structural properties of Cu_2O films electrodeposited on thin Au were characterized by XRD. Compared to the films electrodeposited on bare FTO substrates, the introduction of the Au film enhanced the [111] orientation of Cu_2O [11]. No impurity phases were observed in the XRD profiles, indicating the successful electrodeposition of single-phase Cu_2O.

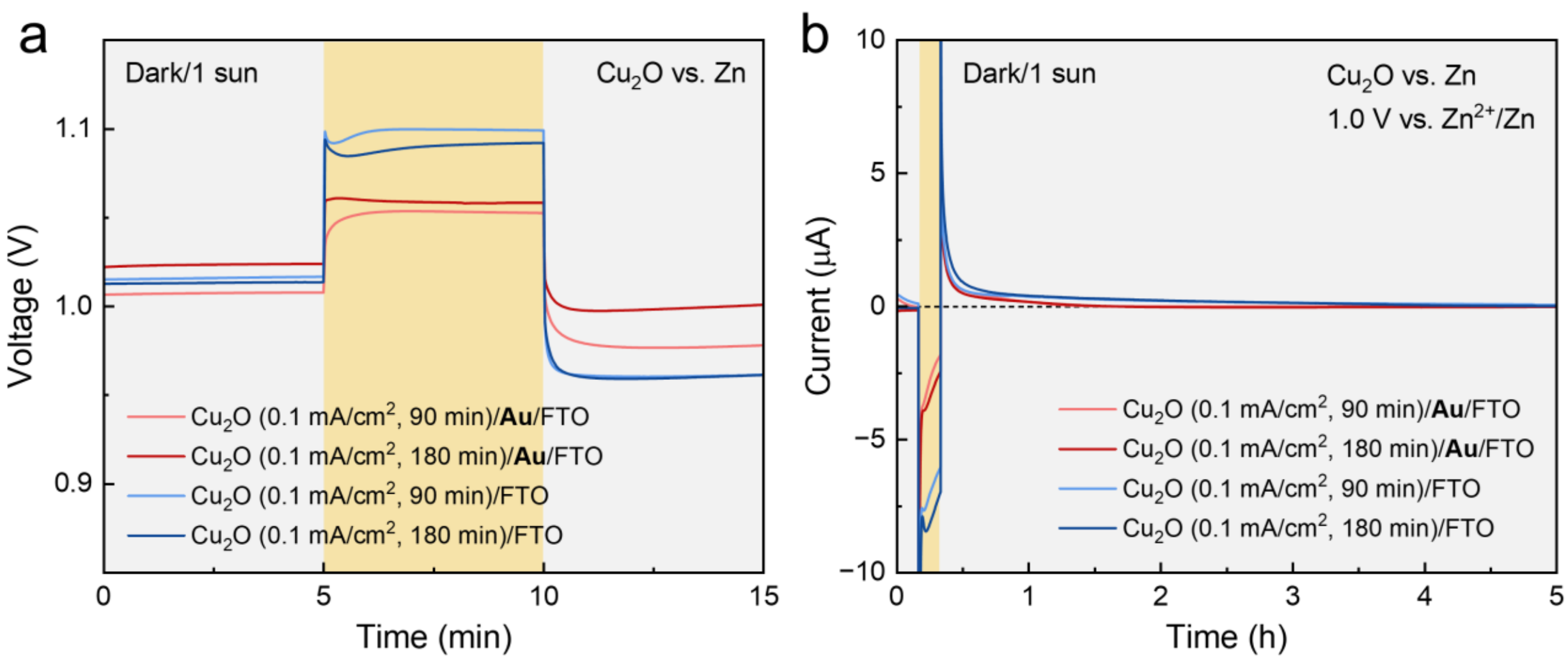

Fig. S30 | Photoresponse of the Cu_2O/Au electrodes. The photoactivities of Cu_2O/Au electrodes were compared with those without thin Au films using (a) OCV and (b) CA measurements under chopped light illumination. Although the Cu_2O/Au films were compact and well-oriented, the photoresponse was not enhanced; this is likely because the rougher morphology of Cu_2O on bare FTO was more beneficial for light trapping and increasing the active surface area.

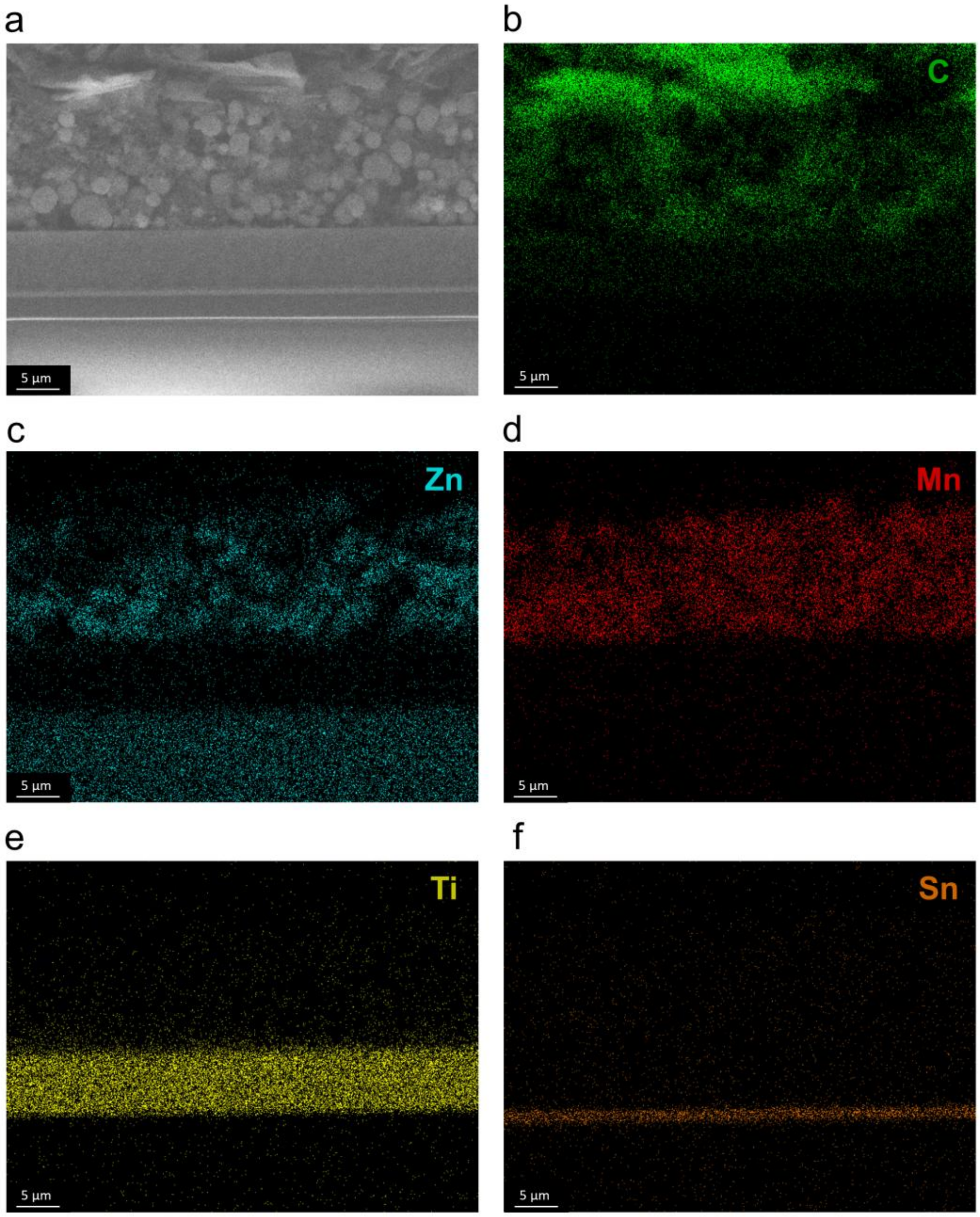

Fig. S31 | Cross-sectional SEM/EDS mapping of the layer-by-layer photoelectrode. (a) Cross-sectional SEM image and corresponding EDS elemental maps of (b) C, (c) Zn, (d) Mn, (e) Ti, and (f) Sn show distinct layered structures without obvious intermixing.

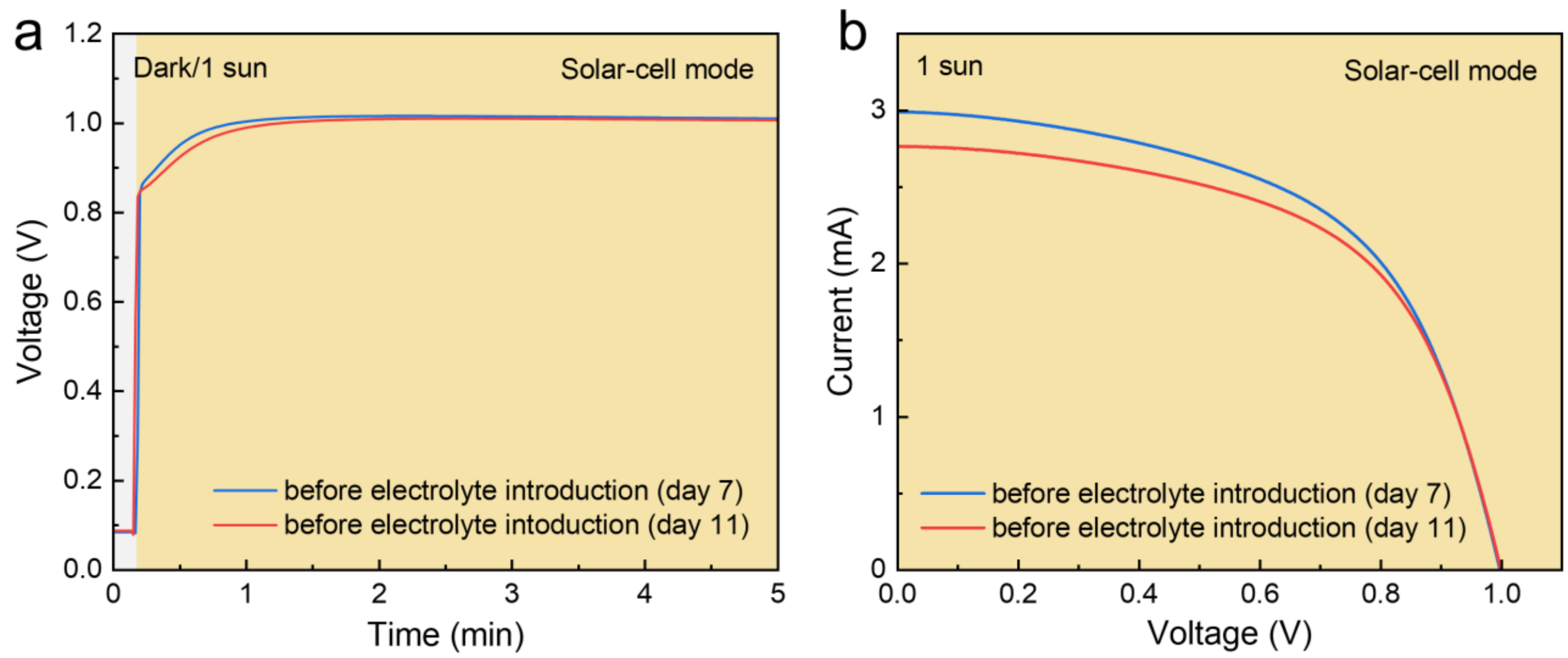

Fig. S32 | Photovoltage of the layer-by-layer photoelectrode prior to electrolyte introduction. The stability of the layer-by-layer photoelectrode before electrolyte introduction was evaluated using (a) OCV and (b) *I*–*V* curves. The photovoltage remained stable at approximately 1.0 V over 11 days; however, it decreased to ~0.7 V upon electrolyte injection, as shown in the manuscript.

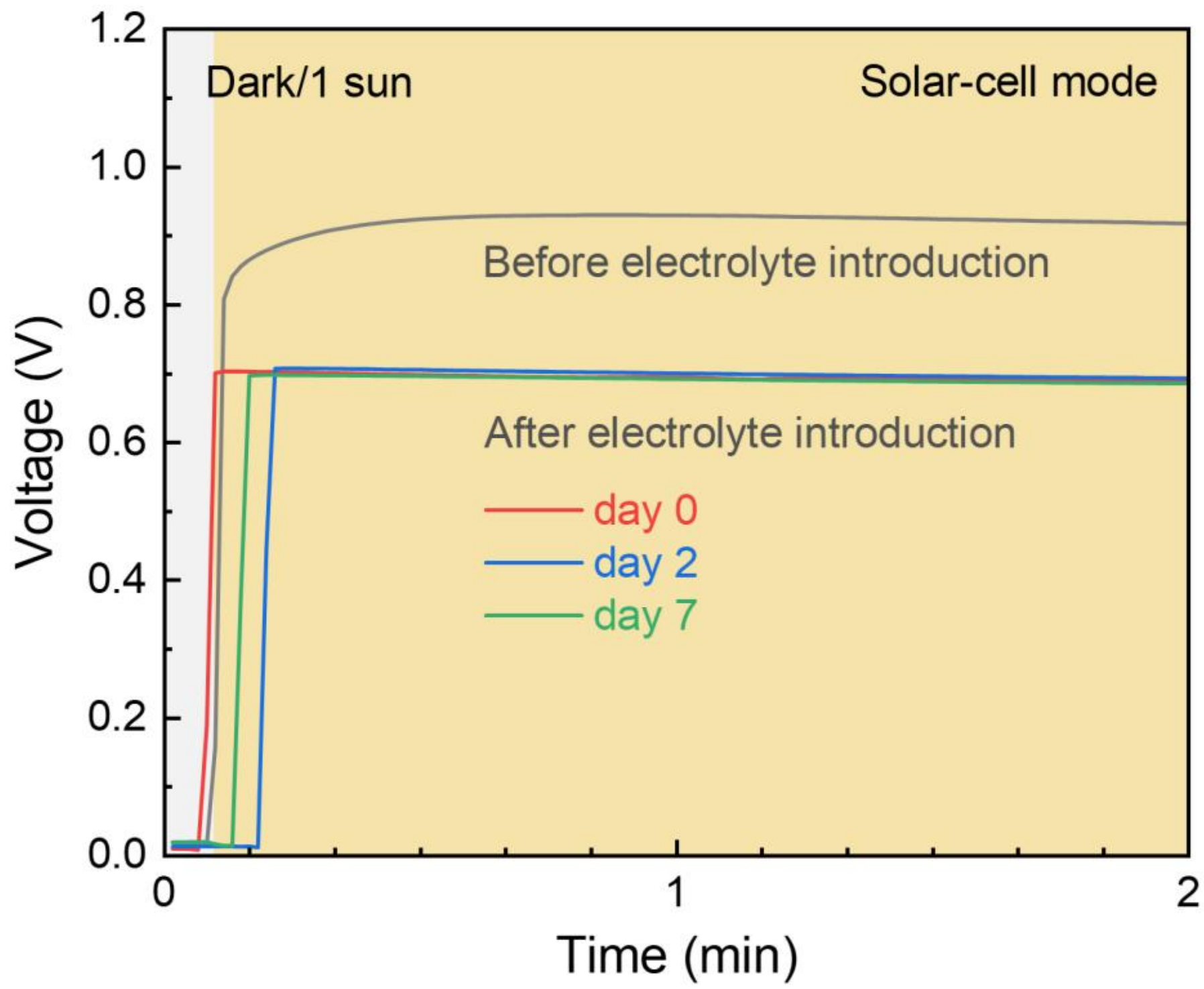

Fig. S33 | Photovoltage of the layer-by-layer photoelectrode following electrolyte introduction. The stability of the photovoltage after electrolyte injection was monitored over one week. The photovoltage under 1-sun illumination remained at approximately 0.7 V with a stable profile, suggesting that the significant photovoltage change observed upon electrolyte introduction was not due to a degradation process.

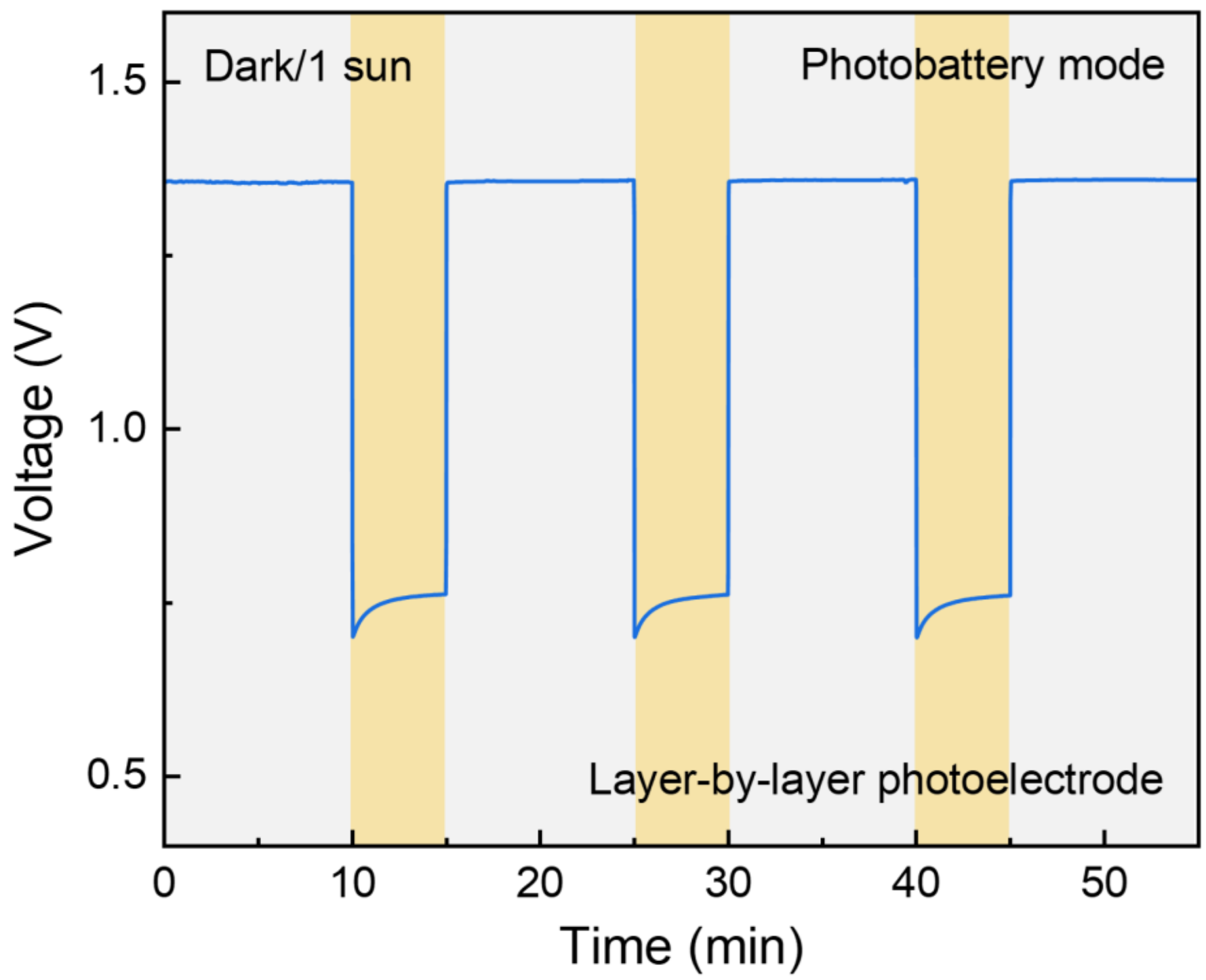

Fig. S34 | Photovoltage of the layer-by-layer photoelectrode in photobattery mode. OCV response of the layer-by-layer photoelectrode after electrolyte introduction was measured under chopped light illumination in photobattery mode, which is the same configuration as the conventional two-electrode system.

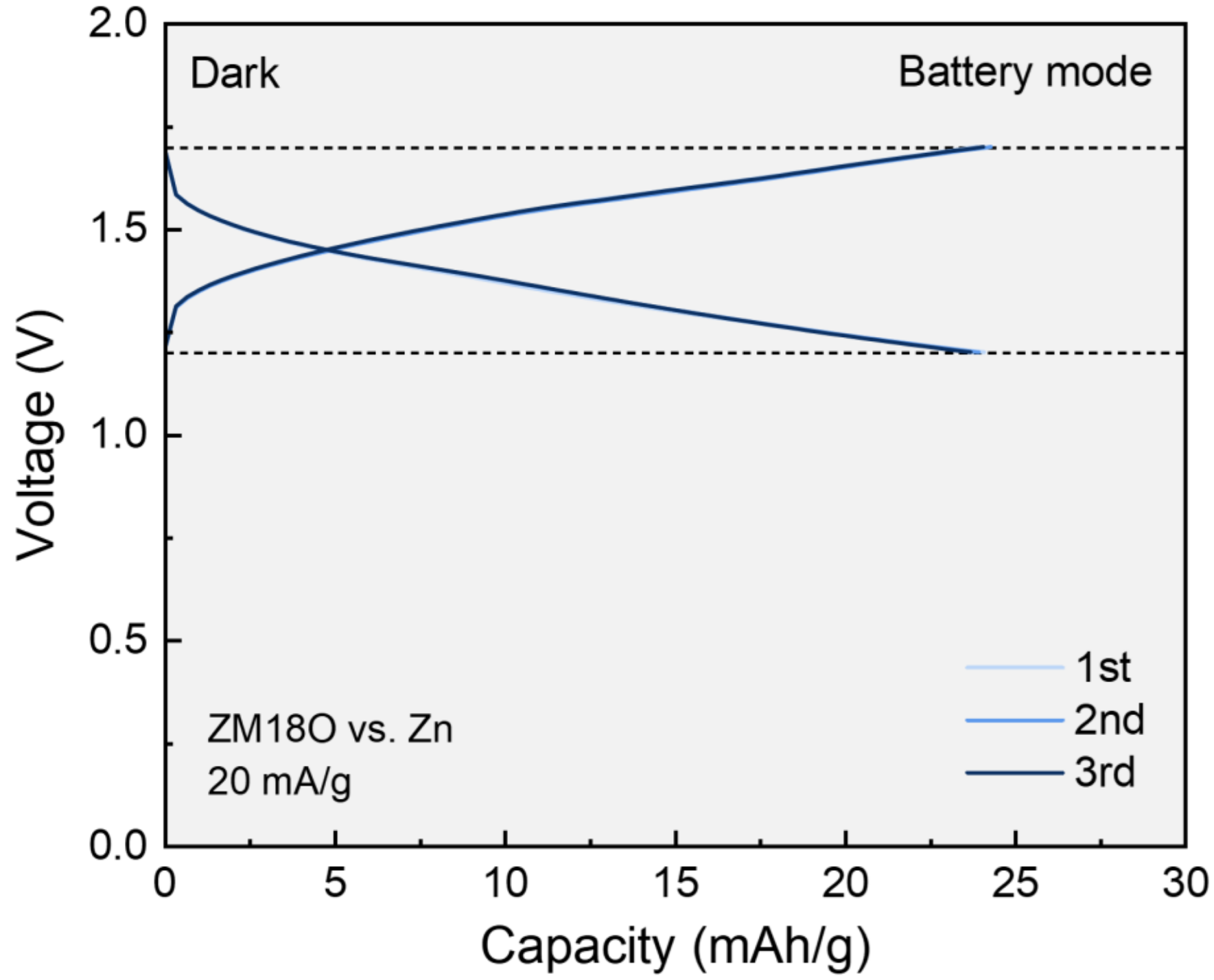

Fig. S35 | Pre-cycling of the ZMO cathode in battery mode. After assembling the layer-by-layer photobattery device, the cathode material was galvanostatically cycled in dark prior to further characterization. Stable cycling performance was observed within the first 3 cycles; however, the obtained capacity was limited to approximately 25 mAh/g. This is likely attributed to the constrained potential window employed for cycling and the increased internal resistance compared to standard coin cells.

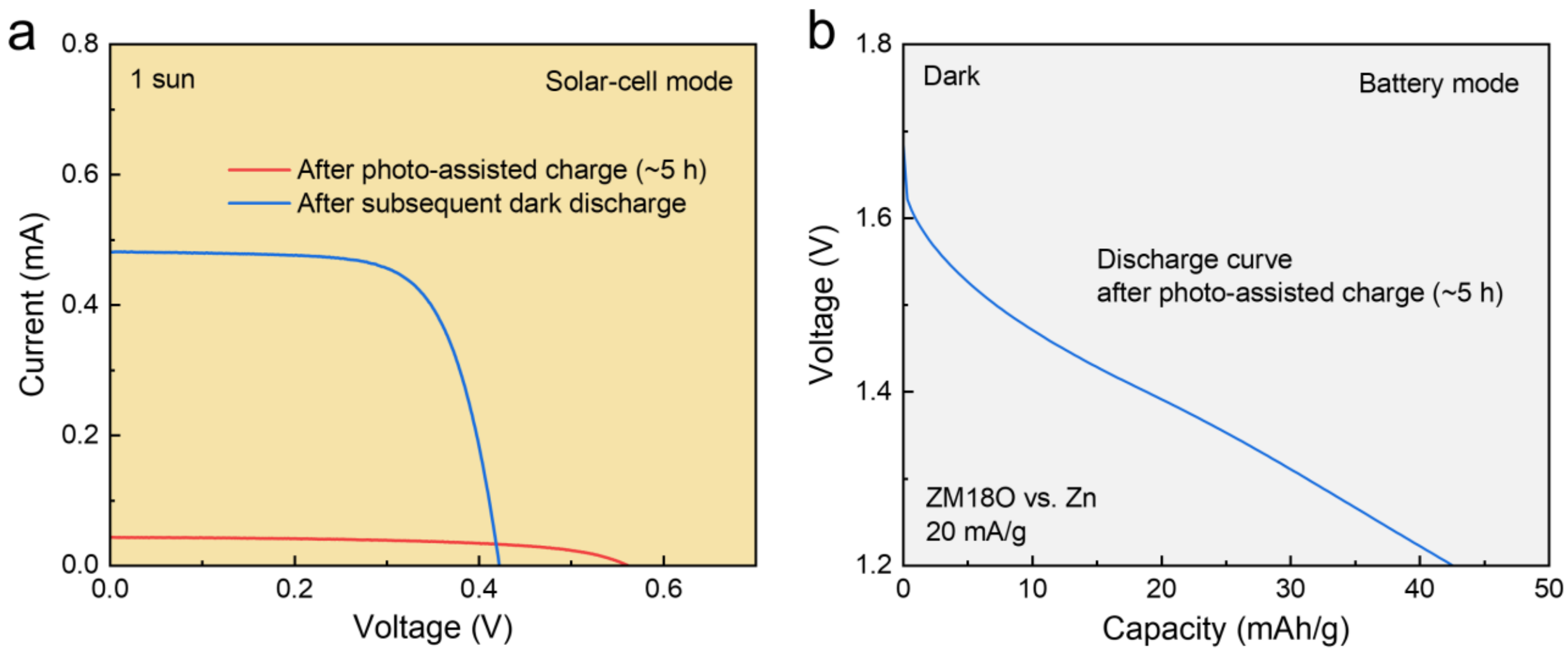

Fig. S36 | Solar-cell performance following photo-assisted charging and dark discharging. (a) *I–V* curves were obtained after the photo-assisted charging (~5 h) and (b) subsequent dark discharging processes. While the photocurrent significantly decreased to less than 0.1 mA after the photo-assisted charging, it recovered to around 0.5 mA after the dark discharging. It should be noted that the gradual decrease in overall device performance, compared to initial states, is attributed to device degradation during the extended operation.

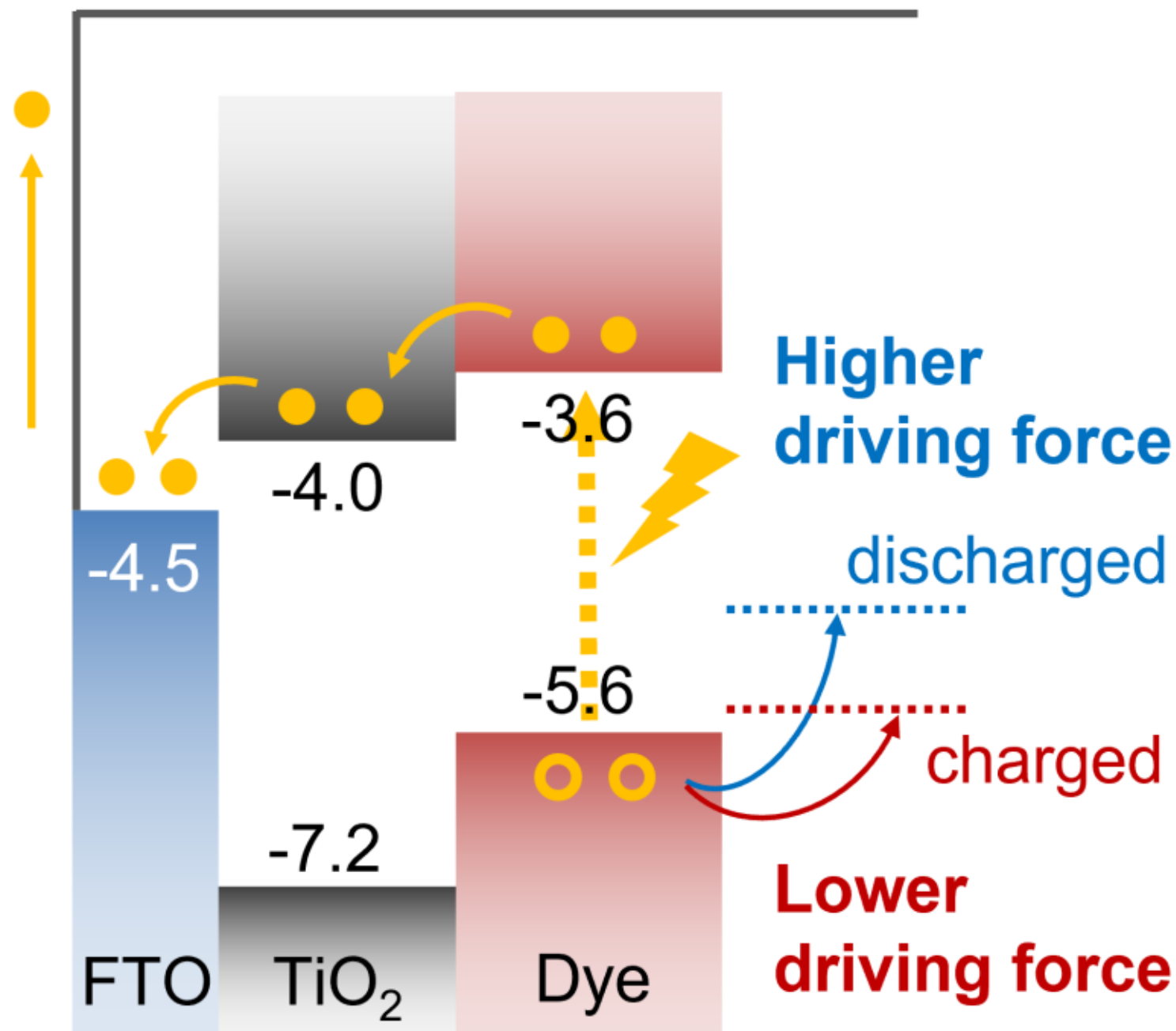

Fig. S37 | Schematic energy level diagram showing the photo-assisted charging driving force. The energetic driving force for hole transfer from the dye through the HTL to the cathode is higher in the discharged state and diminishes as the cathode potential shifts upward in the charged state. The numbers in the figure indicate the electron energy levels relative to the vacuum level.

Note S1 | Calculation of solar-to-chemical energy conversion efficiency.

The overall solar-to-chemical energy conversion efficiency ($\eta_{overall}$) of the photobattery was calculated based on the framework established in previous literature [6]:

$$\eta_{overall} = \frac{E_{output}}{E_{input\ light}} = \frac{\int (V_{dark\ CC} - V_{holding})dC}{I_{input\ light}\ \times\ A_{active\ area}\ \times\ t_{photo}}$$

where $V_{holding}$ represents the constant potential bias applied during photo-assisted charging (1.2 V vs. Zn^{2+}/Zn), which is subtracted from the discharge potential ($V_{dark\ CC}$) to isolate the net output energy contributed strictly by solar illumination.

Based on the photo-assisted charging and dark discharging profiles presented in Fig. 4e:

- Incident light energy ($E_{input\ light}$): $100\ \text{mW/cm}^2\ \times 1\ \text{cm}^2\ \times\ 3600\ \text{s}\ = 360\ \text{J}$
- Net stored discharge energy above 1.2 V (E_{output}): $\sim 0.015\ \text{mWh}\ =\ 0.054\ \text{J}$

Consequently, the overall solar-to-chemical energy conversion efficiency is calculated as:

$$\eta_{overall} = \frac{0.054\ \text{J}}{360\ \text{J}} \times\ 100\ =\ 0.015\%$$

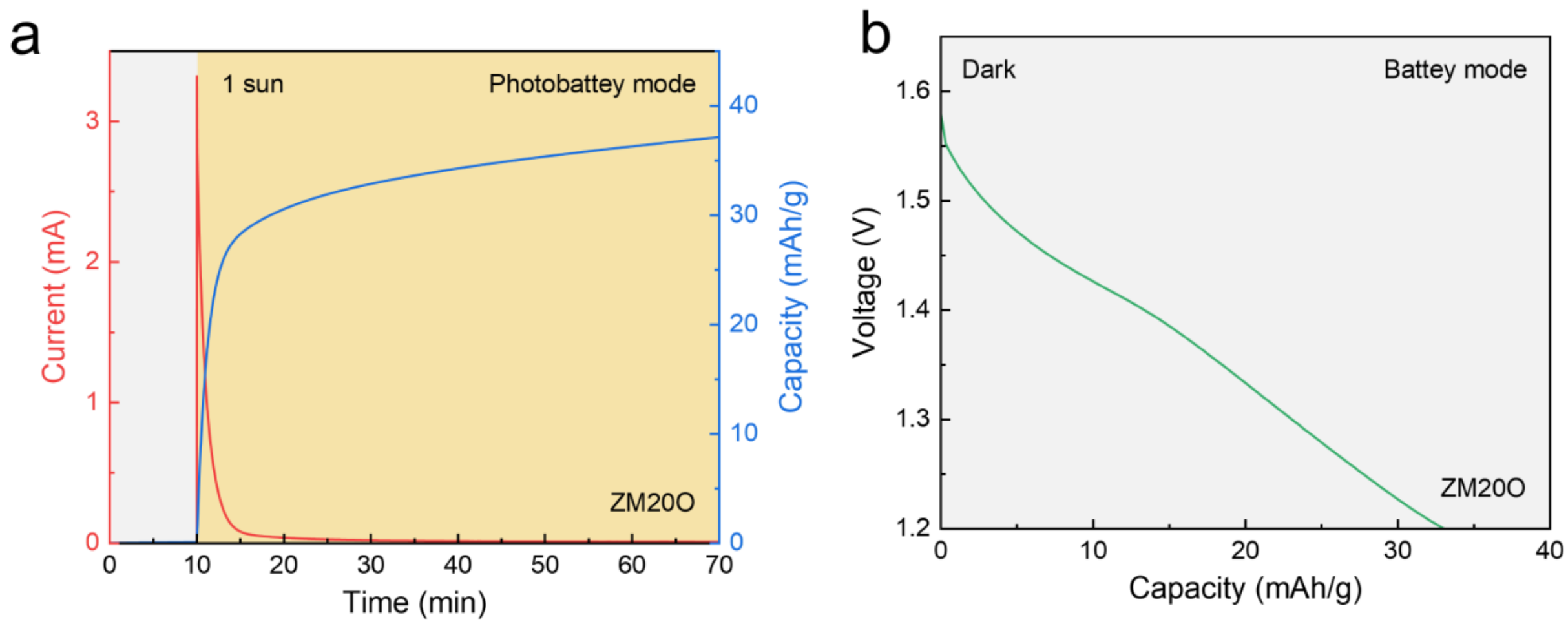

Fig. S38 | Photo-assisted charge/discharge of the layer-by-layer photoelectrode with ZM20O. A layer-by-layer photoelectrode was fabricated using ZM20O as the cathode, and its performance was evaluated. (a) Photo-assisted charge and (b) subsequent dark discharge behavior was basically similar to that in the case of ZM18O, while the discharge capacity was slightly higher in ZM18O, possibly due to the enhanced diffusion kinetics.

Table S7 | Sequential experimental protocols for the layer-by-layer photobattery devices. Four devices were fabricated with ZM20O or ZM18O cathodes, but they short-circuited during the dark battery process before or after the photo-assisted charge/discharge steps, probably due to the dendritic growth of the Zn anode.

	Device 1 (ZM20O)	Device 2 (ZM20O)	Device 3 (ZM18O)	Device 4 (ZM18O)
Step 1	Dark battery cycle	Dark battery cycle	Dark battery cycle	Dark battery cycle
Step 2	Photo-assisted charge	**(Short-circuited)**	Photo-assisted charge	**(Short-circuited)**
Step 3	Dark discharge		Dark discharge	
Step 4	Dark battery cycle		Dark battery cycle	
Step 5	**(Short-circuited)**		Photo-assisted charge	
Step 6			Dark discharge	
Step 7			Dark battery cycle	
Step 8			**(Short-circuited)**	

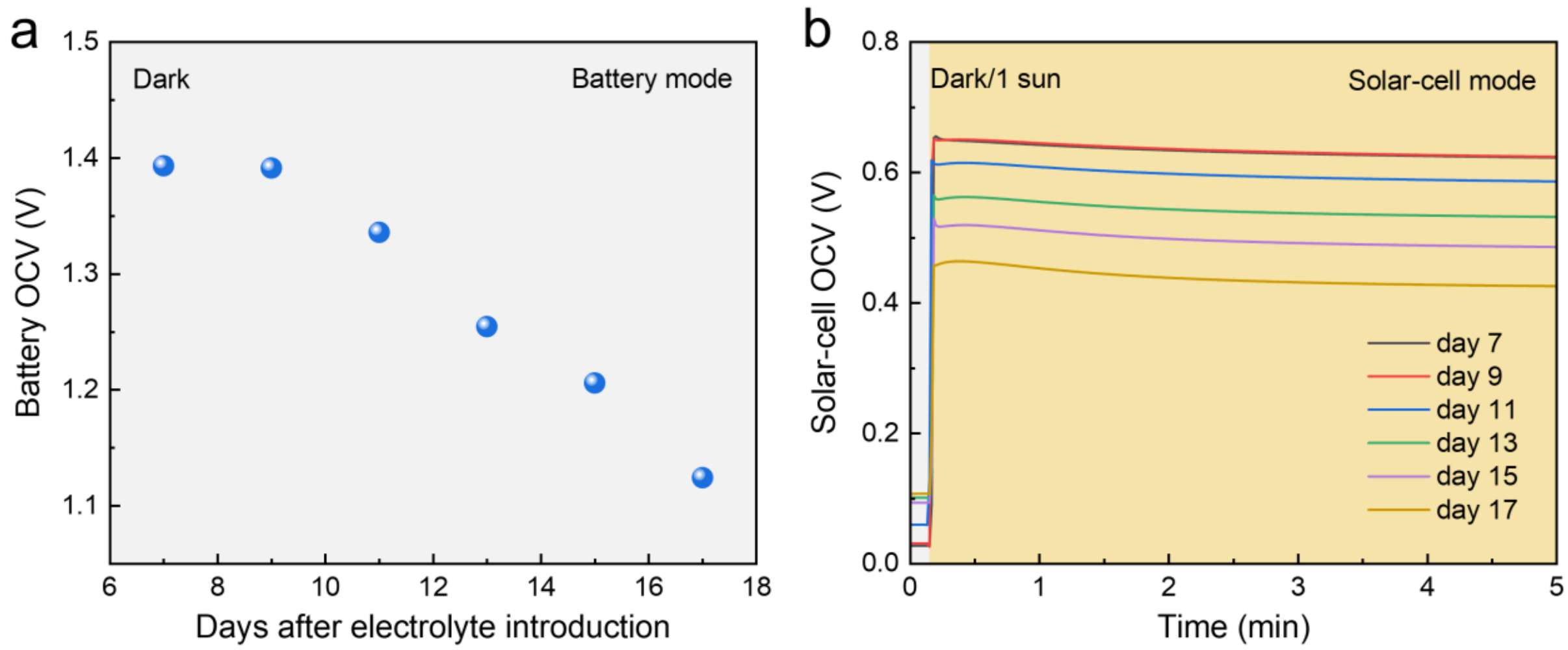

Fig. S39 | Self-discharge behavior and its impact on solar-cell performance. (a) A near-linear decrease in the battery OCV was observed from day 9 to day 17, following a stable period until day 9. (b) The solar-cell OCV under 1-sun illumination exhibited a corresponding decline during the same period (from day 9 to day 17). These results demonstrate a strong correlation between the solar-cell performance and the battery state-of-charge.

References

(1) Yagi, S.; Ichikawa, Y.; Yamada, I.; Doi, T.; Ichitsubo, T.; Matsubara, E. Synthesis of binary magnesium–transition metal oxides via inverse coprecipitation. *Japanese Journal of Applied Physics* **2013**, *52* (2R), 025501.

(2) Paracchino, A.; Laporte, V.; Sivula, K.; Grätzel, M.; Thimsen, E. Highly active oxide photocathode for photoelectrochemical water reduction. *Nature Materials* **2011**, *10* (6), 456–461.

(3) Izumi, F.; Momma, K. Three-dimensional visualization in powder diffraction. *Solid State Phenomena* **2007**, *130*, 15–20.

(4) Momma, K.; Izumi, F. VESTA 3 for three-dimensional visualization of crystal, volumetric and morphology data. *Applied Crystallography* **2011**, *44* (6), 1272–1276.

(5) Tan, L.; Kim, B.-M.; Pujari, A.; He, Z.; Boruah, B. D.; De Volder, M. Photothermal enhancement of Prussian blue cathodes for Li-ion batteries. *Nano Letters* **2024**, *24* (30), 9147–9154.

(6) Pujari, A.; Shimokawa, K.; De Volder, M. Photobatteries: Prospects and Fundamental Limitations. *Joule* **2025**, *9* (3), 101869.

(7) Shinagawa, T.; Ida, Y.; Mizuno, K.; Watase, S.; Watanabe, M.; Inaba, M.; Tasaka, A.; Izaki, M. Controllable growth orientation of Ag_2O and Cu_2O films by electrocrystallization from aqueous solutions. *Crystal Growth & Design* **2013**, *13* (1), 52–58.

(8) Makuła, P.; Pacia, M.; Macyk, W. How to correctly determine the band gap energy of modified semiconductor photocatalysts based on UV–Vis spectra. *The Journal of Physical Chemistry Letters* **2018**, *9* (23), 6814–6817.

(9) Dias, P.; Schreier, M.; Tilley, S. D.; Luo, J.; Azevedo, J.; Andrade, L.; Bi, D.; Hagfeldt, A.; Mendes, A.; Grätzel, M. Transparent cuprous oxide photocathode enabling a stacked tandem cell for unbiased water splitting. *Advanced Energy Materials* **2015**, *5* (24), 1501537.

(10) He, J.; Jiang, Y.; Peng, J.; Li, C.; Yan, B.; Wang, X. Fast synthesis of hierarchical cuprous oxide for nonenzymatic glucose biosensors with enhanced sensitivity. *Journal of Materials Science* **2016**, *51* (21), 9696–9704.

(11) Pan, L.; Dai, L.; Burton, O. J.; Chen, L.; Andrei, V.; Zhang, Y.; Ren, D.; Cheng, J.; Wu, L.; Frohna, K. High carrier mobility along the [111] orientation in Cu_2O photoelectrodes. *Nature* **2024**, *628* (8009), 765–770.
